# Large expert-curated database for benchmarking document similarity detection in biomedical literature search

**DOI:** 10.1093/database/baz085

**Published:** 2019-10-29

**Authors:** Peter Brown, RELISH Consortium, Aik-Choon Tan, Mohamed A El-Esawi, Thomas Liehr, Oliver Blanck, Douglas P Gladue, Gabriel M F Almeida, Tomislav Cernava, Carlos O Sorzano, Andy W K Yeung, Michael S Engel, Arun Richard Chandrasekaran, Thilo Muth, Martin S Staege, Swapna V Daulatabad, Darius Widera, Junpeng Zhang, Adrian Meule, Ken Honjo, Olivier Pourret, Cong-Cong Yin, Zhongheng Zhang, Marco Cascella, Willy A Flegel, Carl S Goodyear, Mark J van Raaij, Zuzanna Bukowy-Bieryllo, Luca G Campana, Nicholas A Kurniawan, David Lalaouna, Felix J Hüttner, Brooke A Ammerman, Felix Ehret, Paul A Cobine, Ene-Choo Tan, Hyemin Han, Wenfeng Xia, Christopher McCrum, Ruud P M Dings, Francesco Marinello, Henrik Nilsson, Brett Nixon, Konstantinos Voskarides, Long Yang, Vincent D Costa, Johan Bengtsson-Palme, William Bradshaw, Dominik G Grimm, Nitin Kumar, Elvis Martis, Daniel Prieto, Sandeep C Sabnis, Said E D R Amer, Alan W C Liew, Paul Perco, Farid Rahimi, Giuseppe Riva, Chongxing Zhang, Hari P Devkota, Koichi Ogami, Zarrin Basharat, Walter Fierz, Robert Siebers, Kok-Hian Tan, Karen A Boehme, Peter Brenneisen, James A L Brown, Brian P Dalrymple, David J Harvey, Grace Ng, Sebastiaan Werten, Mark Bleackley, Zhanwu Dai, Raman Dhariwal, Yael Gelfer, Marcus D Hartmann, Pawel Miotla, Radu Tamaian, Pragashnie Govender, Oliver J Gurney-Champion, Joonas H Kauppila, Xiaolei Zhang, Natalia Echeverría, Santhilal Subhash, Hannes Sallmon, Marco Tofani, Taeok Bae, Oliver Bosch, Páraic O Cuív, Antoine Danchin, Barthelemy Diouf, Tuomas Eerola, Evangelos Evangelou, Fabian V Filipp, Hannes Klump, Lukasz Kurgan, Simon S Smith, Olivier Terrier, Neil Tuttle, David B Ascher, Sarath C Janga, Leon N Schulte, Daniel Becker, Christopher Browngardt, Stephen J Bush, Guillaume Gaullier, Kazuki Ide, Clement Meseko, Gijsbert D A Werner, Jan Zaucha, Abd A Al-Farha, Noah F Greenwald, Segun I Popoola, Md Shaifur Rahman, Jialin Xu, Sunny Y Yang, Noboru Hiroi, Ozgul M Alper, Chris I Baker, Michael Bitzer, George Chacko, Birgit Debrabant, Ray Dixon, Evelyne Forano, Matthew Gilliham, Sarah Kelly, Karl-Heinz Klempnauer, Brett A Lidbury, Michael Z Lin, Iseult Lynch, Wujun Ma, Edward W Maibach, Diane E Mather, Kutty S Nandakumar, Robert S Ohgami, Piero Parchi, Patrizio Tressoldi, Yu Xue, Charles Armitage, Pierre Barraud, Stella Chatzitheochari, Luis P Coelho, Jiajie Diao, Andrew C Doxey, Angélique Gobet, Pingzhao Hu, Stefan Kaiser, Kate M Mitchell, Mohamed F Salama, Ivan G Shabalin, Haijun Song, Dejan Stevanovic, Ali Yadollahpour, Erliang Zeng, Katharina Zinke, C G Alimba, Tariku J Beyene, Zehong Cao, Sherwin S Chan, Michael Gatchell, Andreas Kleppe, Marcin Piotrowski, Gonzalo Torga, Adugna A Woldesemayat, Mehmet I Cosacak, Scott Haston, Stephanie A Ross, Richard Williams, Alvin Wong, Matthew K Abramowitz, Andem Effiong, Senhong Lee, Muhammad Bilal Abid, Cyrus Agarabi, Cedric Alaux, Dirk R Albrecht, Gerald J Atkins, Charles R Beck, A M J J Bonvin, Emer Bourke, Thomas Brand, Ralf J Braun, James A Bull, Pedro Cardoso, Dee Carter, Robin M Delahay, Bernard Ducommun, Pascal H G Duijf, Trevor Epp, Eeva-Liisa Eskelinen, Mazyar Fallah, Debora B Farber, Jose Fernandez-Triana, Frank Feyerabend, Tullio Florio, Michael Friebe, Saori Furuta, Mads Gabrielsen, Jens Gruber, Malgorzata Grybos, Qian Han, Michael Heinrich, Heikki Helanterä, Michael Huber, Albert Jeltsch, Fan Jiang, Claire Josse, Giuseppe Jurman, Haruyuki Kamiya, Kim de Keersmaecker, Erik Kristiansson, Frank-Erik de Leeuw, Jiuyong Li, Shide Liang, Jose A Lopez-Escamez, Francisco J Lopez-Ruiz, Kevin J Marchbank, Rolf Marschalek, Carmen S Martín, Adriana E Miele, Xavier Montagutelli, Esteban Morcillo, Rosario Nicoletti, Monika Niehof, Ronan O’Toole, Toshihiko Ohtomo, Henrik Oster, Jose-Alberto Palma, Russell Paterson, Mark Peifer, Maribel Portilla, M C Portillo, Antonia L Pritchard, Stefan Pusch, Gajendra P S Raghava, Nicola J Roberts, Kehinde Ross, Birgitt Schuele, Kjell Sergeant, Jun Shen, Alessandro Stella, Olga Sukocheva, Vladimir N Uversky, Sven Vanneste, Martin H Villet, Miguel Viveiros, Julia A Vorholt, Christof Weinstock, Masayuki Yamato, Ioannis Zabetakis, Xin Zhao, Andreas Ziegler, Wan M Aizat, Lauren Atlas, Kristina M Bridges, Sayan Chakraborty, Mieke Deschodt, Helena S Domingues, Shabnam S Esfahlani, Sebastian Falk, J L Guisado, Nolan C Kane, Gray Kueberuwa, Colleen L Lau, Dai Liang, Enwu Liu, Andreas M Luu, Chuang Ma, Lisong Ma, Robert Moyer, Adam D Norris, Suresh Panthee, Jerod R Parsons, Yousong Peng, Inês Mendes Pinto, Cristina R Reschke, Elina Sillanpää, Christopher J Stewart, Florian Uhle, Hui Yang, Kai Zhou, Shu Zhu, Mohamed Ashry, Niels Bergsland, Maximilian Berthold, Chang-Er Chen, Vito Colella, Maarten Cuypers, Evan A Eskew, Xiao Fan, Maksymilian Gajda, Rayner Gonzálezlez-Prendes, Amie Goodin, Emily B Graham, Ewout J N Groen, Alba Gutiérrez-Sacristán, Mohamad Habes, Enrico Heffler, Daniel B Higginbottom, Thijs Janzen, Jayakumar Jayaraman, Lindsay A Jibb, Stefan Jongen, Timothy Kinyanjui, Rositsa G Koleva-Kolarova, Zhixiu Li, Yu-Peng Liu, Bjarte A Lund, Alexandre A Lussier, Liping Ma, Pablo Mier, Matthew D Moore, Katja Nagler, Mark W Orme, James A Pearson, Anilkumar S Prajapati, Yu Saito, Simon E Tröder, Florence Uchendu, Niklas Verloh, Denitza D Voutchkova, Ahmed Abu-Zaid, Joaira Bakkach, Philipp Baumert, Marcos Dono, Jack Hanson, Sandrine Herbelet, Emma Hobbs, Ameya Kulkarni, Narendra Kumar, Siqi Liu, Nikolai D Loft, Tristan Reddan, Thomas Senghore, Howard Vindin, Haotian Xu, Ross Bannon, Branson Chen, Johnny T K Cheung, Jeffrey Cooper, Ashwini K Esnakula, Karine A Feghali, Emilia Ghelardi, Agostino Gnasso, Jeffrey Horbar, Hei M Lai, Jian Li, Lan Ma, Ruiyan Ma, Zihang Pan, Marco A Peres, Raymond Pranata, Esmond Seow, Matthew Sydes, Ines Testoni, Anna L Westermair, Yongliang Yang, Masoud Afnan, Joan Albiol, Lucia G Albuquerque, Eisuke Amiya, Rogerio M Amorim, Qianli An, Stig U Andersen, John D Aplin, Christos Argyropoulos, Yan W Asmann, Abdulaziz M Assaeed, Atanas G Atanasov, David A Atchison, Simon V Avery, Paul Avillach, Peter D Baade, Lars Backman, Christophe Badie, Alfonso Baldi, Elizabeth Ball, Olivier Bardot, Adrian G Barnett, Mathias Basner, Jyotsna Batra, O M Bazanova, Andrew Beale, Travis Beddoe, Melanie L Bell, Eugene Berezikov, Sue Berners-Price, Peter Bernhardt, Edward Berry, Theolis B Bessa, Craig Billington, John Birch, Randy D Blakely, Mark A T Blaskovich, Robert Blum, Marleen Boelaert, Dimitrios Bogdanos, Carles Bosch, Thierry Bourgoin, Daniel Bouvard, Laura M Boykin, Graeme Bradley, Daniel Braun, Jeremy Brownlie, Albert Brühl, Austin Burt, Lisa M Butler, Siddappa N Byrareddy, Hugh J Byrne, Stephanie Cabantous, Sara Calatayud, Eva Candal, Kimberly Carlson, Sònia Casillas, Valter Castelvetro, Patrick T Caswell, Giacomo Cavalli, Vaclav Cerovsky, Monica Chagoyen, Chang-Shi Chen, Dong F Chen, Hao Chen, Hui Chen, Jui-Tung Chen, Yinglong Chen, Changxiu Cheng, Jianlin Cheng, Mai Chinapaw, Christos Chinopoulos, William C S Cho, Lillian Chong, Debashish Chowdhury, Andre Chwalibog, A Ciresi, Shamshad Cockcroft, Ana Conesa, Penny A Cook, David N Cooper, Olivier Coqueret, Enoka M Corea, Elisio Costa, Carol Coupland, Stephanie Y Crawford, Aparecido D Cruz, Huijuan Cui, Qiang Cui, David C Culver, Amedeo D’Angiulli, Tanya E S Dahms, France Daigle, Raymond Dalgleish, Håvard E Danielsen, Sébastien Darras, Sean M Davidson, David A Day, Volkan Degirmenci, Luc Demaison, Koenraad Devriendt, Jiandong Ding, Yunus Dogan, X C Dong, Claudio F Donner, Walter Dressick, Christian A Drevon, Huiling Duan, Christian Ducho, Nicolas Dumaz, Bilikere S Dwarakanath, Mark H Ebell, Steffen Eisenhardt, Naser Elkum, Nadja Engel, Timothy B Erickson, Michael Fairhead, Marty J Faville, Marlena S Fejzo, Fernanda Festa, Antonio Feteira, Patrick Flood-Page, John Forsayeth, Simon A Fox, Steven J Franks, Francesca D Frentiu, Mikko J Frilander, Xinmiao Fu, Satoshi Fujita, Ian Galea, Luca Galluzzi, Federica Gani, Arvind P Ganpule, Antonio García-Alix, Kristene Gedye, Maurizio Giordano, Cecilia Giunta, Paul A Gleeson, Cyrille Goarant, Haipeng Gong, Diop Gora, Michael J Gough, Ravinder Goyal, Kathryn E Graham, Ana Grande-Pérez, Patricia M Graves, Harm Greidanus, Darren Grice, Christoph Grunau, Yosephine Gumulya, Yabin Guo, Vsevolod V Gurevich, Oleg Gusev, Elke Hacker, Steffen R Hage, Guy Hagen, Steven Hahn, Dagmar M Haller, Sven Hammerschmidt, Jianwei Han, Renzhi Han, Martin Handfield, Hapuarachchige C Hapuarachchi, Timm Harder, Jennifer E Hardingham, Michelle Heck, Marcel Heers, Khe F Hew, Yohei Higuchi, Cynthia St Hilaire, Rachel Hilton, Enisa Hodzic, Andrew Hone, Yuichi Hongoh, Guoku Hu, Heinz P Huber, Luis E Hueso, Judith Huirne, Lisa Hurt, Helena Idborg, Kazuho Ikeo, Evan Ingley, Philip M Jakeman, Arne Jensen, Hong Jia, Husen Jia, Shuqin Jia, Jianping Jiang, Xingyu Jiang, Yi Jin, Daehyun Jo, Andrew M Johnson, Marie Johnston, Karen R Jonscher, Philippe G Jorens, Jens O L Jorgensen, Johan W Joubert, Sin-Ho Jung, Antonio M Junior, Thomas Kahan, Sunjeev K Kamboj, Yong-Kook Kang, Yannis Karamanos, Natasha A Karp, Ryan Kelly, Ralph Kenna, Jonathan Kennedy, Birgit Kersten, Roy A Khalaf, Javaria M Khalid, T Khatlani, Tarig Khider, Gregor S Kijanka, Sarah R B King, Tomasz Kluz, Paul Knox, Tatsuya Kobayashi, Karl-Wilhelm Koch, Maija R J Kohonen-Corish, Xiangpeng Kong, Deborah Konkle-Parker, Kalevi M Korpela, Leondios G Kostrikis, Peter Kraiczy, Harald Kratz, Günter Krause, Paul H Krebsbach, Søren R Kristensen, Prerna Kumari, Akira Kunimatsu, Hatice Kurdak, Young D Kwon, Carl Lachat, Malgorzata Lagisz, Brenda Laky, Jan Lammerding, Matthias Lange, Mar Larrosa, Andrew L Laslett, Elizabeth E LeClair, Kyung-Woo Lee, Ming-Yih Lee, Moon-Soo Lee, Genyuan Li, Jiansheng Li, Klaus Lieb, Yau Y Lim, Merry L Lindsey, Paul-Dag Line, Dengcai Liu, Fengbin Liu, Haiyan Liu, Hongde Liu, Vett K Lloyd, Te-Wen Lo, Emanuela Locci, Josef Loidl, Johan Lorenzen, Stefan Lorkowski, Nigel H Lovell, Hua Lu, Wei Lu, Zhiyong Lu, Gustavo S Luengo, Lars-Gunnar Lundh, Philippe A Lysy, Angela Mabb, Heather G Mack, David A Mackey, S R Mahdavi, Pamela Maher, Toby Maher, Sankar N Maity, Brigitte Malgrange, Charalampos Mamoulakis, Arduino A Mangoni, Thomas Manke, Antony S R Manstead, Athanasios Mantalaris, Jan Marsal, Hanns-Ulrich Marschall, Francis L Martin, Jose Martinez-Raga, Encarnacion Martinez-Salas, Daniel Mathieu, Yoichi Matsui, Elie Maza, James E McCutcheon, Gareth J McKay, Brian McMillan, Nigel McMillan, Catherine Meads, Loreta Medina, B Alex Merrick, Dennis W Metzger, Frederic A Meunier, Martin Michaelis, Olivier Micheau, Hisaaki Mihara, Eric M Mintz, Takuo Mizukami, Yann Moalic, D P Mohapatra, Antonia Monteiro, Matthieu Montes, John V Moran, Sergey Y Morozov, Matthew Mort, Noriyuki Murai, Denis J Murphy, Susan K Murphy, Shauna A Murray, Shinji Naganawa, Srinivas Nammi, Grigorios Nasios, Roman M Natoli, Frederique Nguyen, Christine Nicol, Filip van Nieuwerburgh, Erlend B Nilsen, Clarissa J Nobile, Margaret O’Mahony, Sophie Ohlsson, Oluremi Olatunbosun, Per Olofsson, Alberto Ortiz, Kostya Ostrikov, Siegmar Otto, Tiago F Outeiro, Songying Ouyang, Sabrina Paganoni, Andrew Page, Christoph Palm, Yin Paradies, Michael H Parsons, Nick Parsons, Pigny Pascal, Elisabeth Paul, Michelle Peckham, Nicoletta Pedemonte, Michael A Pellizzon, M Petrelli, Alexander Pichugin, Carlos J C Pinto, John N Plevris, Piero Pollesello, Martin Polz, Giovanna Ponti, Piero Porcelli, Martin Prince, Gwendolyn P Quinn, Terence J Quinn, Satu Ramula, Juri Rappsilber, Florian Rehfeldt, Jan H Reiling, Claire Remacle, Mohsen Rezaei, Eric W Riddick, Uwe Ritter, Neil W Roach, David D Roberts, Guillermo Robles, Tiago Rodrigues, Cesar Rodriguez, Jo Roislien, Monique J Roobol, J Alexandra Rowe, Andreas Ruepp, Jan van Ruitenbeek, Petra Rust, Sonia Saad, George H Sack, Manuela Santos, Aurore Saudemont, Gianni Sava, Simone Schrading, Alexander Schramm, Martin Schreiber, Sidney Schuler, Joost Schymkowitz, Alexander Sczyrba, Kate L Seib, Han-Ping Shi, Tomohiro Shimada, Jeon-Soo Shin, Colette Shortt, Patricia Silveyra, Debra Skinner, Ian Small, Paul A M Smeets, Po-Wah So, Francisco Solano, Daniel E Sonenshine, Jiangning Song, Tony Southall, John R Speakman, Mandyam V Srinivasan, Laura P Stabile, Andrzej Stasiak, Kathryn J Steadman, Nils Stein, Andrew W Stephens, Douglas I Stewart, Keith Stine, Curt Storlazzi, Nataliya V Stoynova, Wojciech Strzalka, Oscar M Suarez, Taranum Sultana, Anirudha V Sumant, Mathew J Summers, Gang Sun, Paul Tacon, Kozo Tanaka, Haixu Tang, Yoshinori Tanino, Paul Targett-Adams, Mourad Tayebi, Reema Tayyem, Christoph C Tebbe, Evelyn E Telfer, Wolfram Tempel, Julita A Teodorczyk-Injeyan, Gert Thijs, Sally Thorne, Amanda G Thrift, Celine Tiffon, Philip Tinnefeld, Daryono H Tjahjono, Fabrice Tolle, Ervin Toth, Andria L del Tredici, Apostolos Tsapas, Konstantinos Tsirigotis, Ayse Turak, George Tzotzos, Edet E Udo, Toshiaki Utsumi, Subramanian Vaidyanathan, Michel Vaillant, Armand Valsesia, Roosmarijn E Vandenbroucke, Feliciano H Veiga, Marc Vendrell, Peter A Vesk, Paul Vickers, Victor M Victor, Richard Villemur, Marie-Claude Vohl, Christian R Voolstra, Anne Vuillemin, Steven Wakelin, Levi Waldron, Laurence J Walsh, Amanda Y Wang, Fuan Wang, Yun Wang, Yoichi Watanabe, Andreas Weigert, Jet-Chau Wen, Carol Wham, Ethan P White, Jan Wiener, Gottfried Wilharm, Simon Wilkinson, Raffaella Willmann, Coralie Wilson, Brunhilde Wirth, Timothy R Wojan, Mathieu Wolff, Bryan M Wong, Tzu-Wei Wu, Hanno Wuerbel, Xiangshu Xiao, Dong Xu, J W Xu, Jianping Xu, Bin Xue, Suayib Yalcin, Hong Yan, En-Cheng Yang, Shiqi Yang, Wei Yang, Yuzhen Ye, Zhi-Qiang Ye, Jari Yli-Kauhaluoma, Hiroshi Yoneyama, Ying Yu, Guo-Cheng Yuan, Chiou-Hwa Yuh, Manuela Zaccolo, Chen Zeng, Branko Zevnik, Chi Zhang, Li Zhang, Li Zhang, Yingkai Zhang, Yusen Zhang, Zhiyong Zhang, Zhong-Yin Zhang, Yuan Zhao, Min Zhou, Torsten Zuberbier, Carmen M Aanei, Rafi Ahmad, Manar Al-Lawama, Alexandre Alanio, Judith Allardyce, David Alonso-Caneiro, John M Atack, Dirk Baier, Abhisheka Bansal, Yannick Benezeth, Colette Berbesque, Frederik Berrevoet, Peter H W Biedermann, Erik Bijleveld, Florian Bittner, Fabian Blombach, Wouter van den Bos, Shellie A Boudreau, Adam D Bramoweth, Oliver Braubach, Yufeng Cai, Matthew Campbell, Zanxia Cao, Thibault Catry, Xin Chen, Shuiqin Cheng, Hee-Jung Chung, Miguel A Chávez-Fumagalli, Aaron Conway, Bruno M Costa, Normand Cyr, Lorraine T Dean, Martin S Denzel, S V Dlamini, Kevin J Dudley, Maeva Dufies, Thorsten Ecke, Denitsa Eckweiler, Elisenda Eixarch, Hosny El-Adawy, Julius V Emmrich, Alex J Eustace, Christine M Falter-Wagner, Johannes Fuss, Jianzhao Gao, Martin R Gill, Liz Gloyn, Robert Goggs, Usha Govinden, Garrett Greene, Victor Greiff, D S Grundle, Patrick Grüneberg, Nicksy Gumede, Gbaguidi Haore, Pille Harrison, Xavier Hoenner, Diego Hojsgaard, Hikaru Hori, Maria P Ikonomopoulou, Patrick Jeurissen, Daniel M Johnson, Dhiraj Kabra, Koji Kamagata, Chandan Karmakar, Olga Kasian, Linda K Kaye, Murad M Khan, Yong-Min Kim, J K Kish, Sebastian Kobold, Gary Kohanbash, Gregor Kohls, Jan-Michael Kugler, Gyanendra Kumar, Jon Lacy-Colson, Asam Latif, Volker M Lauschke, Bingling Li, Chinten J Lim, Fang Liu, Xiaodong Liu, Jin-Jian Lu, Qiang Lu, Poornima Mahavadi, Ugo Marzocchi, Christine A McGarrigle, Tom van Meerten, Rogier Min, Iain Moal, Massimiliano Molari, Lucas Molleman, Saiful R Mondal, Thea van de Mortel, W N Moss, Othonas A Moultos, Maheswari Mukherjee, Kazuhiko Nakayama, Edward Narayan, Navaratnarajah, Philipp-Alexander Neumann, Jiyun Nie, Yingjiu Nie, Frank Niemeyer, Fiona Nolan, Ogueri Nwaiwu, Wendy H Oldenmenger, Emmanuel Olumayede, Jianhong Ou, Menuka Pallebage-Gamarallage, Simon P Pearce, Tuula Pelkonen, Maria C Pelleri, Joana L Pereira, Mpho Pheko, Karina A Pinto, Allison Piovesan, Michael Pluess, Illya M Podolsky, Julie Prescott, Dongchen Qi, Xingshun Qi, Vaia D Raikou, Andreas Ranft, Johanna Rhodes, Jean-Yves Rotge, Anna D Rowe, Manish Saggar, Robert A Schuon, Shaouli Shahid, Vahid Shalchyan, Prasad Shirvalkar, Oleg Shiryayev, Jugpreet Singh, Michael J Smout, António Soares, Chunjiao Song, Kshitij Srivastava, Rupesh K Srivastava, Jim Sun, Attila Szabo, Wiktor Szymanski, Chan N P Tai, Hisashi Takeuchi, S Tanadini-Lang, Fei Tang, Wanyin Tao, G Theron, Chang F Tian, Yu-Shi Tian, Lisa M Tuttle, Anna Valenti, Pierre Verlot, Mirella Walker, Jun Wang, Danielle Welter, Matthew Winslade, Dalei Wu, Yi-Rui Wu, Han Xiao, Beisi Xu, Juan Xu, Ziyue Xu, Dongdong Yang, Mingjun Yang, Patricio Yankilevich, Yuyi You, Chenglong Yu, Jian Zhan, Gong Zhang, Kai Zhang, Tuo Zhang, Yi Zhang, Guoyan Zhao, Jing Zhao, Xiaofan Zhou, Zhenxing Zhu, Penelope A Ajani, Udunna C Anazodo, Saeed A Bagloee, Kasia Bail, Ido Bar, Joe Bathelt, David Benkeser, Meghan L Bernier, Adam M Blanchard, Dominic W Boakye, Vasileios Bonatsos, Michele H Boon, George Bouboulis, Elizabeth Bromfield, Joshua Brown, Kim C M Bul, Kathryn J Burton, Eugene G Butkowski, Grace Carroll, Fengqing Chao, Elisabeth E Charrier, Xiaoyin Chen, Yu-Chih Chen, Chenguang, Jane R Choi, Tore Christoffersen, João C Comel, Cyril Cosse, Yanru Cui, Pieter van Dessel, Dhaval, Daria Diodato, Maelle Duffey, Avik Dutt, Luis G Egea, Mohammed El-Said, Martin Faye, Beatriz Fernandez-Fernandez, Kieran G Foley, Luria L Founou, Fan Fu, Rabea A Gadelkareem, Evgeny Galimov, Gulcan Garip, Alison Gemmill, Quentin Gouil, James Grey, Zoya Gridneva, Michel J Grothe, Théophile Grébert, Fabricio Guerrero, Léo Guignard, Marco J Haenssgen, David Hasler, Joan Y Holgate, Ancheng Huang, Amanda M Hulse-Kemp, Claire Jean-Quartier, Sang-Min Jeon, Yangyang Jia, Catherine Jutzeler, Panagiotis Kalatzis, Masud Karim, Kathrin Karsay, Anne Keitel, Andreas Kempe, Jeremy R Keown, Chin M Khoo, Nyil Khwaja, Rogier A Kievit, Aleksandra Kosanic, Dimitrios A Koutoukidis, Paul Kramer, Dilip Kumar, Nükhet Kırağ, Giuseppe Lanza, Thuc D Le, Jung W Leem, Daniel Leightley, Andreia Leite, Lukas Lercher, Ying Li, Renly Lim, Luiz R A Lima, Li Lin, Tong Ling, Yuchen Liu, Zhonghua Liu, Yao Lu, Fok M Lum, Hang Luo, Jatin Machhi, Angus Macleod, Isaac Macwan, Hanumantha R Madala, Nima Madani, Nicola de Maio, Kalina Makowiecki, Daniel J Mallinson, Ruta Margelyte, Caracausi Maria, Y Markonis, Luca Marsili, Suzanne Mavoa, Lorna McWilliams, Moa Megersa, Caetano S M Mendes, Julia Menichetti, Rebecca Mercieca-Bebber, Jack J Miller, David-Paul M Minde, Alexander Minges, Eleanor Mishra, Virendra R Mishra, Carly Moores, Nicola Morrice, Alexander E Moskalensky, Nicolò Navarin, Edessa Negera, Philippe Nolet, Ana Nordberg, Rickard Nordén, Jessica P Nowicki, Nelly Olova, Paweł Olszewski, Robert Onzima, Chih-Long Pan, Charny Park, Dong Ik Park, Seyoung Park, Chandrashekhar D Patil, Sansoa A Pedro, Samuel R Perry, Jessica Peter, Brent M Peterson, Andrea Pezzuolo, Ilya Pozdnyakov, Siyu Qian, Lei Qin, Ali Rafe, Ishier Raote, Ali Raza, Henrike Rebl, Osama Refai, Tim Regan, Tambi Richa, Mark F Richardson, K R Robinson, Luca Rossoni, Romain Rouet, Soroush Safaei, Pierre H H Schneeberger, Daniela Schwotzer, Agata Sebastian, Jennifer Selinski, Stefanie Seltmann, Feng Sha, Nir Shalev, Jin-Long Shang, Josef Singer, Mandeep Singh, Taylor Smith, Emma Solomon-Moore, Lijuan Song, Samuele Soraggi, Ryan Stanley, Nico Steckhan, Frederic Strobl, Lorenzo Subissi, Irwan Supriyanto, Chinmay R Surve, Tomo Suzuki, Caitlin Syme, Karl Sörelius, Young Tang, Marwa Tantawy, Sumudu Tennakoon, Serafino Teseo, Christine Toelzer, Nikola Tomov, Miguel Tovar, Linh Tran, Sushil Tripathi, Anil M Tuladhar, Azubuike C Ukubuiwe, Carolina O L Ung, Kaspar Valgepea, Hamid Vatanparast, Arnau Vidal, Fang Wang, Qing Wang, Ricky Watari, Rebecca Webster, Ruth Webster, Junnian Wei, David Wibowo, Tanja S H Wingenbach, Rose M Xavier, Shumin Xiao, Peng Xiong, Shicai Xu, Shilin Xu, Ruifeng Yao, Wen Yao, Qinan Yin, Yongbo Yu, Masayoshi Zaitsu, Zian Zeineb, Xiao-Yong Zhan, Jilei Zhang, Rongqiang Zhang, Wei Zhang, Xianglilan Zhang, Shan Zheng, Bailing Zhou, Xiaoyan Zhou, Haroon Ahmad, Sayo A Akinwumi, Gregory F Albery, Ahmed Alhowimel, Junaid Ali, Mansour Alshehri, Mohammed Alsuhaibani, Andrey Anikin, Samuel O Azubuike, Anders Bach-Mortensen, Lior Baltiansky, Martin Bartas, Kiflemariam Y Belachew, Vivek Bhardwaj, Karin Binder, Nicholas S Bland, Michael Boah, Benjamin Bullen, Giovanna E Calabrò, Tiffany J Callahan, Bing Cao, Kelsey Chalmers, Wei Chang, Zhengping Che, Andrew T Y Chen, Haimin Chen, Huaming Chen, Youning Chen, Zhao Chen, YoungRok Choi, Mohiuddin A K Chowdhury, Martin R Christensen, Robert S C Cooke, Marzia Cottini, Natalie V Covington, Catriona Cunningham, Julien Delarocque, Lucie Devos, Aurup R Dhar, Ke-Feng Ding, Kexian Dong, Zheng Dong, Niklas Dreyer, Chelsea Ekstrand, Tanguy Fardet, Berhanu E Feleke, Thomas Feurer, Angela Freitas, Tian Gao, N G Asefa, Francesco Giganti, Piotr Grabowski, José R Guerra-Mora, Chengying Guo, Xinyi Guo, Himanshu Gupta, Shuonan He, Marloes Heijne, Stephanie Heinemann, Alexander Hogrebe, Zhengping Huang, Sophinese Iskander-Rizk, Lavanya M Iyer, Yasmin Jahan, Ameh S James, Emmanuel Joel, Bastian Joffroy, Clara Jégousse, George Kambondo, Priyanka Karnati, Cihan Kaya, An Ke, Daniel Kelly, Rob Kickert, Peter E Kidibule, Jennifer P Kieselmann, Hyeon J Kim, Takeshi Kitazawa, Aniek Lamberts, You Li, Huakang Liang, Sabrina N Linn, Thomas Litfin, Wang Liusuo, Vasiliki Lygirou, Ajay K Mahato, Zhi-Ming Mai, Rupert W Major, Samira Mali, Panagiotis Mallis, Wenzhi Mao, Wenzhi Mao, Katie Marvin-Dowle, Leanda D Mason, Ben Merideth, Maria J Merino-Plaza, Britt Merlaen, Rossella Messina, Anand K Mishra, Junaid Muhammad, Conrad Musinguzi, Afroditi Nanou, Amreen Naqash, Joe T Nguyen, Thi T H Nguyen, Duan Ni, Nida, Shirli Notcovich, Barnabas Ohst, Quinn R Ollivier, Daniël F Osses, Xiangda Peng, Arnoud Plantinga, Michael Pulia, Muhammad Rafiq, Ayush Raman, Delphine Raucher-Chéné, Rafał Rawski, Asit Ray, Lubna A Razak, Kevin Rudolf, Peter Rusch, Margaux L Sadoine, Axel Schmidt, Roey Schurr, Stephen Searles, Saurab Sharma, Barry Sheehan, Chunhu Shi, Belal Shohayeb, Andrew Sommerlad, Jan Strehlow, Xianbao Sun, Raghav Sundar, Ghazaleh Taherzadeh, Nur D M Tahir, Jun Tang, Jean Testa, Zhiqi Tian, Qian Tingting, Geert P Verheijen, Casey Vickstrom, Teng Wang, Xiaomin Wang, Zhenxing Wang, Pan Wei, Alex Wilson, Wyart, Abdul-Amir Yassine, Abbas Yousefzadeh, Asma Zare, Zhen Zeng, Chengrong Zhang, Haowen Zhang, Linxing Zhang, Tongchuan Zhang, Weijia Zhang, Zhe Zhang, Jianyu Zhou, Dongjie Zhu, Vincenzo Adamo, Adebolajo A Adeyemo, Maria Aggelidou, Adi M Al-Owaifeer, Arwa Z Al-Riyami, Saeed K Alzghari, Vibeke Andersen, Kathryn Angus, Muhammad Asaduzzaman, Hadi Asady, Dai Ato, Xiaoyong Bai, Rebecca L Baines, Maghan Ballantyne, Bo Ban, Jill Beck, Walid Ben-Nafa, Emma Black, Antoine Blancher, Ron Blankstein, Neil Bodagh, Paulo A V Borges, Anastasia Brooks, Josue Brox-Ponce, Arturo Brunetti, Colin D Canham, Piero Carninci, Richard Carvajal, Shun C Chang, Jie Chao, Pranab Chatterjee, He Chen, Yi-Chun Chen, Adnan K Chhatriwalla, Ibrahim Chikowe, Trees-Juen Chuang, Rosane G Collevatti, Diego A Valera-Cornejo, Ana Cuenda, Myriam Dao, Delphine Dauga, Zaian Deng, Kiran Devkota, Lisa V Doan, Yaser H A Elewa, Dongsheng Fan, Mohammed Faruk, Shi Feifei, Trevor S Ferguson, Francesco Fleres, Emma J Foster, C Stephen Foster, Tzvi Furer, Yibo Gao, Enid J Garcia-Rivera, Adi Gazdar, Ronald B George, Sayantan Ghosh, Elena Gianchecchi, Joshua M Gleason, Allan Hackshaw, Adam Hall, Richard Hall, Paul Harper, William E Hogg, Guangqun Huang, Kylie E Hunter, Adriaan P IJzerman, Carlos Jesus, Gao Jian, James S Lewis Jr, Souha S Kanj, Harsheen Kaur, Shona Kelly, Fayez Kheir, V S Kichatova, Musa Kiyani, Reinhild Klein, Tom Kovesi, Jennifer L Kraschnewski, Addanki P Kumar, Dmitry Labutin, Alejandro Lazo-Langner, Guy Leclercq, Maoteng Li, Qingchun Li, Tangliang Li, Yongzhe Li, Wei-Ting Liao, Zheng-yin Liao, Jessica Lin, J Lizer, Giambattista Lobreglio, Cher Lowies, Cheng Lu, Haroon Majeed, Adam Martin, Luis Martinez-Sobrido, Edwin Meresh, Marianne Middelveen, Alireza Mohebbi, Jorge Mota, Zahra Mozaheb, Ley Muyaya, Amar Nandhakumar, Sheryl H X Ng, Monther Obeidat, Deog-Hwan Oh, Mohammed Owais, Pia Pace-Asciak, Ajay Panwar, Caroline Park, Chris Patterson, Felipe Penagos-Tabaree, Paolo T Pianosi, Valentina Pinzi, Clare Pridans, Anna Psaroulaki, Ravi Kumar Pujala, Leonardo Pulido-Arjona, Peng-Fei Qi, Proton Rahman, Nayanjot K Rai, Tienush Rassaf, Julie Refardt, Walter Ricciardi, Olaf Riess, Alexandros Rovas, Frank M Sacks, Sherif Saleh, Christopher Sampson, Axel Schmutz, Robert Sepanski, Neeraj Sharma, Manisha Singh, Paul Spearman, Mehala Subramaniapillai, Ritu Swali, Cher M Tan, Juan I Tellechea, Lisa-Marie Thomas, Xin Tong, Demetrios G Vavvas, Ralf Veys, Veronica Vitriol, Horng-Dar Wang, Jinhui Wang, Jiucun Wang, Jason Waugh, S A Webb, Brendan A Williams, Alan D Workman, Tingxiu Xiang, Li-Xin Xie, Jun Xu, Taosheng Xu, Chongjun Yang, Jihoon G Yoon, Christina M Yuan, Arno Zaritsky, Yao Zhang, Haochen Zhao, Hannah Zuckerman, Ran Lyu, Wayne Pullan, Yaoqi Zhou

**Affiliations:** 1 School of Information and Communication Technology, Griffith University, Gold Coast, QLD 4222, Australia; 2 Institute for Glycomics, Griffith University, Gold Coast, QLD 4222, Australia; 3 See collaborators section

## Abstract

Document recommendation systems for locating relevant literature have mostly relied on methods developed a decade ago. This is largely due to the lack of a large offline gold-standard benchmark of relevant documents that cover a variety of research fields such that newly developed literature search techniques can be compared, improved and translated into practice. To overcome this bottleneck, we have established the **RE**levant **LI**terature **S**earc**H** consortium consisting of more than 1500 scientists from 84 countries, who have collectively annotated the relevance of over 180 000 PubMed-listed articles with regard to their respective seed (input) article/s. The majority of annotations were contributed by highly experienced, original authors of the seed articles. The collected data cover 76% of all unique PubMed Medical Subject Headings descriptors. No systematic biases were observed across different experience levels, research fields or time spent on annotations. More importantly, annotations of the same document pairs contributed by different scientists were highly concordant. We further show that the three representative baseline methods used to generate recommended articles for evaluation (Okapi Best Matching 25, Term Frequency–Inverse Document Frequency and PubMed Related Articles) had similar overall performances. Additionally, we found that these methods each tend to produce distinct collections of recommended articles, suggesting that a hybrid method may be required to completely capture all relevant articles. The established database server located at https://relishdb.ict.griffith.edu.au is freely available for the downloading of annotation data and the blind testing of new methods. We expect that this benchmark will be useful for stimulating the development of new powerful techniques for title and title/abstract-based search engines for relevant articles in biomedical research.

When drafting a manuscript such as this article, a survey of relevant literature and related articles from the same sub-field is required. Traditionally, such a survey is conducted by combining searches based on specific keywords and references found in bibliographies (i.e. references citing, or cited in, the selected articles). This process requires multiple attempts of different keyword combinations and manual examination of numerous citations. A long list of both relevant and irrelevant articles is often encountered requiring manual curation after further reading, yet not all of the important relevant articles are necessarily included after this long and tedious process. The task of finding truly relevant articles becomes increasingly time-consuming and challenging as the number of scientific articles published every year expands at a rapid pace. For example, the PubMed (http://www.ncbi.nlm.nih.gov/pubmed) database operated by the US National Center for Biotechnology Information at the US National Library of Medicine (NLM), has seen a sustained annual growth rate of nearly 5% for the past 4 years; from 25.2 million records in 2015 (57), 26.4 million records in 2016 (58), 27.5 million records in 2017 (59) and is now approaching 29 million records in 2018 as of the latest report (66). The overall success of this classic approach depends on finding optimal keyword combinations as it is key for ensuring the precision and completeness (i.e. sensitivity) of the search results.

The aforementioned scenario is an illustration of an ‘informational’ search goal (8, 64), essentially an exploratory request for discovering the most relevant resources to ‘find out’ about a particular topic. For purely ‘navigational’ queries that seek out a single ‘correct’ result (8, 64), the classical keyword based approach is generally sufficient. However, for a more complete survey, the keyword input given by the user is susceptible to a number of rigidities including misspellings, ambiguity and desired scope. To address these challenges, traditional search engine operators implement various workarounds including query spelling correction, query suggestions, query reformulation and query expansion. A recent NLM study describing ‘under-the-hood’ improvements to PubMed demonstrate the integration of such mitigation techniques (25). As this problem remains unsolved, alternative approaches may be employed.

One way to avoid keywords in searches is to use a seed article. A seed article can provide the title, the abstract and even its full text, containing significantly more information than a combination of a few pre-selected keywords. Aside from these content-based features, seed articles may also provide additional metadata including the list of authors, publication venue and date, citation information and potentially manually curated categorical information such as assigned Medical Subject Headings (MeSH) descriptors (52). Most importantly, all of these features are extracted and processed automatically without requiring any user input or intervention. Thus, for an article-based search, the search engine will accept as input either the selection of an existing document from its database or a user uploaded document in its entirety, in order to generate the list of results.

Article-based relevance searches, often referred to as research paper recommender systems, is an active area of research with more than 100 different approaches published since 1998 (7). Representative examples of commonly used methods used for calculating the similarity between text documents are Okapi Best Matching 25 (BM25) (39, 40), Term Frequency–Inverse Document Frequency (TF-IDF) (65) and PubMed-Related Articles (PMRA) (49). These and other decade-old methods remain the backbone behind real-world recommender systems such as PubMed (http://www.ncbi.nlm.nih.gov/pubmed), ResearchGate (http://www.researchgate.net) and CiteULike (http://www.citeulike.org). Numerous newly developed methods are not yet translated into practice because it is unclear whether they have improved over existing methods (7).

Improvements in paper recommender systems can be measured by ‘user studies’, ‘online evaluations’ or ‘offline evaluations’. User studies aim to quantify user experience by interacting directly with explicit testers of the system; online evaluations are a more passive approach wherein user activity is silently monitored and recorded for assessment, and offline evaluations are akin to traditional benchmarking processes where algorithmic performance measures are evaluated on appropriate datasets. The problem with user studies and online evaluations is that considerable active participation is required to infer any meaningful conclusions (7). Accordingly, a large-scale user study would require costly recruitment and management of a large number of participants, whereas for online evaluations it is challenging to attract large numbers of active users to a deployed system except for a few systems with large established user bases. Moreover, the data used for evaluating such systems are often not made publicly available. In addition, online evaluations collect implicit measures of satisfaction such as click-through rates; however, non-clicked items are not necessarily an indicator of negative samples.

Offline evaluations, on the other hand, require a gold-standard benchmark for comparing and contrasting the performances of different systems. Previous work has focused on building gold standards of query-to-document relevance in biomedical literature. Queries are generally defined as some topic comprised as a natural language statement, a question or a set of keywords conveying a specific information need. Representative examples of existing gold-standard query-to-document resources include the OSHUMED (32) collection, the first TREC Genomics track (33, 73) (the only applicible biomedical-focused track within TREC), the BioASQ (72) competition and the bioCADDIE (13) dataset. Although these datasets are suitable for benchmarking traditional information retrieval, they do not consider the similarity between document pairs as a whole. Re-purposing query-to-document datasets to the document-to-document similarity problem has been attempted; for example, adaptation of the 2004/2005 TREC Genomics data (34, 35) has been evaluated by previous studies (11, 49, 74). However, a pair of documents may both be related to a certain topic but only loosely related to each other as complete units.

Constructing a gold standard dataset of sufficient size for offline evaluations is not a trivial endeavor. In the context of query-to-document relevance, a recent study concluded that ‘there is unfortunately no existing dataset that meets the need for a machine-learning-based retrieval system for PubMed, and it is not possible to manually curate a large-scale relevance dataset’ (24). We propose here that relevance labels between document pairs in the document-to-document context can be ‘crowd-sourced’. This ideology has a number of successful applications; for example, computational resources are crowd-sourced by the BIOINC (1) distributed computing system for the Folding@Home (47) and Rosetta@Home (28) projects, and human problem solving resources are crowd-sourced for ‘distributed thinking systems’ as in the Foldit (14, 28) project.

Here, we have established the **RE**levant **LI**terature **S**earc**H** (RELISH) consortium of 1500+ scientists from 84 countries around the world. The consortium annotated over 180,000 document–document pairs indexed by PubMed, nearly 400 times larger than the single other human annotated data collection we could find with just 460 annotations by 90 authors (50). Analysis of the collected data indicates diversity and consistency of annotations across different levels of research experience as well as its usefulness in benchmarking different document–document comparison methods. Futhermore, these data could be utilized in future work to train deep machine learning models that may substantially increase relevancy of recommendations compared to existing methods.

## Methods

The overall procedure for establishing the RELISH database was as follows. First, we established the article-based PubMed search engine (APSE; https://pubmed.ict.griffith.edu.au) for recommending articles (‘candidate-articles’) potentially relevant to an input article (‘seed-article’) given by, or assigned to, a user. The APSE allowed users to assess and annotate recommended candidate articles regarding their degree of relevance to a respective seed article and facilitated submission of these annotations. Then, we established the RELISH consortium of scientific authors (‘participants’) and invited them to the APSE to evaluate 60 candidate articles for one or more seed articles that they are interested in or, preferably, they have authored. Finally, annotations submitted by participants were compiled and organized into the RELISH database. A participant’s ‘contribution’ was defined as the submission of annotations for all 60 candidate articles with respect to a seed article.

The remainder of this section explains database construction, including the APSE’s corpus data and candidate article recommendation methods, participant recruitment and annotation procedures and performance evaluation metrics.

### APSE: document corpus

The seed article corpus used by the APSE server was extracted from the biomedical literature database hosted by PubMed. The corpus database was constructed using the 2017 baseline downloaded from PubMed’s public FTP server (ftp://ftp.ncbi.nlm.nih.gov/pubmed), followed by incremental synchronization with daily update releases. Only articles with available title and abstract metadata were included in the corpus, no other selection criteria were imposed. As of 3 July 2018, this collection contained 18,345,070 articles, from which participants selected seed articles.

The candidate article corpus, from which the APSE server generated recommendations that were presented to participants, was a subset of the seed article corpus. This subset included only recent articles that were published within the past decade. The primary justification for this was to increase participant motivation with potential discovery of recent work within their field, considering the decline of participation willingness with increasing publication age as speculated previously (77). As of 3 July 2018, this candidate article subset contained 8,730,584 articles.

All raw article texts were pre-processed into usable indexing elements (tokens) by the following pipeline. We first applied Lucene’s ‘UAX29URLEmailTokenizer’, which follows Unicode Text Segmentation rules (16) to divide the text (while also tagging detected URLs or email addresses). Next, possessives (trailing apostrophe + s) were removed from each token, and any single character, numeric, URL or email tokens were removed. Stop-word tokens were filtered according to a list of 132 official MEDLINE stop words retrieved from a previous source (6). Finally, tokens were stemmed to their root form using Lucene’s ‘PorterStemFilter’.

### APSE: recommendation system

The APSE recommendation system is built on three baseline methods: PMRA (49), BM25 (39, 40) and TF-IDF (65). PMRA is the technique used by the ‘Similar Articles’ function on the official PubMed site. Here we used the Entrez E-utilities (43) ELink to retrieve related articles for given seed articles. BM25 is a representative probabilistic relevance technique, implemented here by Lucene’s ‘BM25Similarity’ class using default parameters of $k1 = 1.2$ and $b = 0.75$. TF-IDF is a representative vector space model technique, implemented here by Lucene’s ‘ClassicSimilarity’ class.

For the individual method evaluations presented in this study, the original method-specific list of results were used (in this case the same candidate article may be shared by multiple methods). Each method’s list of candidate articles was sorted by descending score. To generate the list of recommendations presented to the user for annotation, we took the individual method-specific result lists and combined them into a unified list. We set a maximum of 60 articles per seed article so as to include at least the top 20 highest-scoring non-redundant candidate articles per method.

The unified list assembly procedure is as follows: in a round-robin fashion between all methods, consider the current top-scoring article from a method. If the article is not already in the unified list, add it. Otherwise, disregard this article and continue with the next best-scoring article until an addition to the unified list has been made. Essentially, this procedure is performing a three-way merge while ensuring an equal selection of non-redundant recommended articles from each method. This unified list of recommendations was not presented in the order of predicted similarity. Instead, before presentation to users, it was shuffled randomly to reduce the possibility of a systematic bias in annotation.

### RELISH: participant recruitment

To achieve the goal of a large dataset facilitating future deep machine learning-based method development, we established the RELISH consortium of biomedical researchers who have annotated studies potentially related to one or more seed articles that they had authored or were interested in. To maximize the quality and efficiency of annotation, we encouraged participants to use articles that they had authored as the seed articles. All participants contributed voluntarily. Only submissions with annotations completed for all 60 recommended candidate articles per seed article were accepted.

Our recruitment strategy involved two major phases: the first was internal referrals, personal invitations, social media posts and a correspondence letter (9); the second was directly contacting authors of papers published in 2018. For this secondary phase, we built a contact list of the corresponding authors with 2018 papers indexed in PubMed Central (63) and created a personalized email including the top three candidate articles (the highest-scoring non-redundant document from each method) relating to their article along with an invitation to join our consortium.

A total of 129,190 unique authors were contacted regarding 72,764 unique PubMed articles. Of these articles, 54,071 (74.3%) had contact information for a single author only (assumed to be the corresponding author), 7452 (10.2%) had contact information for two authors (assumed to be first and corresponding authors), while the remainder had contact information for more than two authors. In any case, all available contacts per article were invited, as to encourage multiple annotators of the same article for consistency analysis.

Finally, of the 1570 total unique participants, we had 342 (21.8%) participants join by the initial recruitment phase. During the secondary recruitment stage, 1228 (78.2%) authors accepted our invitation yielding an overall response rate of 0.95%, thus the majority of contributions resulted from this phase.

### RELISH: annotation procedure

**Figure 1:**
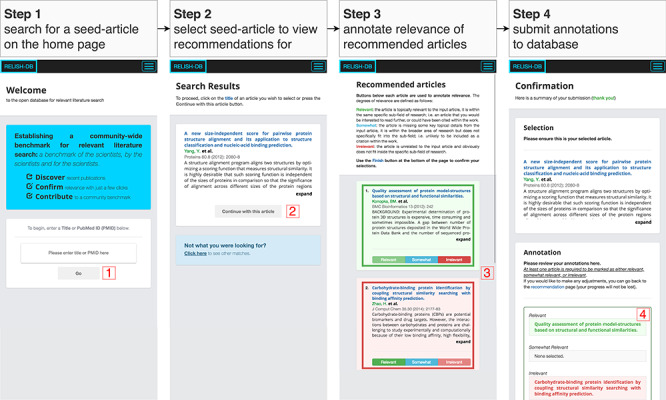
An overview of the four stages comprising the annotation procedure. In step 1, the title or PubMedID of a desired seed article is searched; in step 2, a seed article is selected; in step 3, the result list is presented to the participant for annotation; and in step 4, all annotations are reviewed before being submitted to the database.

An overview of the annotation procedure is shown in Figure 1. Each participant was asked to annotate the degree of relevance (‘relevant’, ‘somewhat-relevant’ or ‘irrelevant’) between a seed article and recommended candidate articles. We gave the following definitions for the degrees of relevance as a guide for label assignment:

1. **Relevant**: an article topically relevant to the seed article; within the same specific sub-field of research, i.e. an article that would be interesting to read further or could have been cited within the original work.
2. **Somewhat-relevant**: an article missing some key topical details of the seed article; within the broader area of research but does not specifically fit into the sub-field, i.e. unlikely to be considered for a citation within the original work.
3. **Irrelevant**: an article unrelated to the seed article and obviously does not fit inside the specific sub-field of research.

Although definitions of ‘relevance’ are subjective, we aimed to make the choice as simple as possible by using this three-point system of document pair ‘closeness’, as opposed to a higher number of relevance degrees, like the 5-point or 10-point scales (44). Additionally, this three-point scale befits collapse to binary classification (a two-point ‘positive’ or ‘negative’ scale); the ‘relevant’ and ‘irrelevant’ classes intuitively map to the ‘positive’ and ‘negative’ classes, respectively, leaving only the ‘somewhat-relevant’ class to be mapped to either the ‘positive’ or ‘negative’ class depending on required evaluation strictness.

Before submitting annotations, contributors were asked to provide their level of experience from the following options: PhD student, years of experience after PhD studies (less than 5, between 5 to 10 or more than 10) and other (unspecified, potentially comparable to PhD experience with degrees like MD or PharmD).

### Evaluation metrics

The expert-annotated document pairs provided the opportunity to evaluate the performance of the three baseline methods for the first time. To simplify the evaluation, we collapsed our three-state label classes to fit a two-state classification in which, unless stated otherwise, ‘relevant’ seed candidate article pairs were assigned to the ‘positive’ class, while ‘somewhat-relevant’ or ‘irrelevant’ article pairs were assigned to the ‘negative’ class. Using this stratification, we assessed method’s performances using both binary classification and information-retrieval metrics.

For binary classification metrics, we used Matthews correlation coefficient (MCC) and area under the [receiver operating characteristic (ROC)] curve (AUC). Metrics used for information-retrieval are precision of top-N ranked results (P@N) and mean reciprocal rank (MRR). MCC and AUC emphasize good discrimination between ‘positive’ and ‘negative’ classes, whereas P@N and MRR metrics emphasize highly ranked ‘positive’ class results.

MCC (54) is the elective metric of the machine learning community for summarizing classification performance; although it is not restricted to binary classification in particular, for the binary case, it coincides with the square root of the chi-squared statistics (42). Using a variable similarity score threshold parameter, contingency tables measuring separation between positive and negative classes are generated, from which the MCC is determined. Here, we have reported the maximum value. A balanced measure is given even when the distribution of ‘positive’ and ‘negative’ class instances are unbalanced (5), where perfect classifications are given a MCC value of 1 and random classifications are given a MCC value of 0.

AUC (29) is determined by a ROC, a plot of sensitivity as a function of specificity at varying threshold parameter values. The area under this ROC curve estimates the probability that a classifier would rank a randomly chosen positive instance higher than a randomly chosen negative instance (23), where perfect classifications are given an AUC value of 1 and random classifications are given an AUC value of 0.5.

P@N (15) is the proportion of results assigned to the ‘positive’ class within a fixed N of top ranked results. Here, we have reported N values of 5, 10 and 20; 5 and 10 were chosen as a measure of ‘first-page’ results initially seen by users, and 20 was used as an indication of the total ‘positive’ class proportion per method (as there only 20 results considered per seed article for individual method evaluation). However, P@N is limited by weighting equally each result within the top-N.

MRR (62) measures average closeness of the first ‘positive’ class result to the top of its result list over a sample of queries $Q$. Here, not all positions are weighted equally, highly ranked ‘positive’ class results are rewarded more than lower-ranked ones. It is determined by averaging the reciprocal rank (RR), i.e. $1/rank_i$, where $rank_i$ is the position of the first encountered ‘positive’ class result in a list of putative matches, for each query in $Q$. An MRR value of 1 indicates that for each query, the highest ranked result belonged to the ‘positive’ class.

### Infrastructure

The database search infrastructure was powered by Elasticsearch [https://github.com/elastic/elasticsearch; using Apache Lucene (https://github.com/apache/lucene-solr)]. The web interface was designed using React (https://github.com/facebook/react), web services were built using Apache (https://github.com/apache/httpd) and Python (https://github.com/channelcat/sanic) and data were organized using MongoDB (https://github.com/mongodb/mongo).

## Results

Here we introduce RELISH-DB, the database that resulted from the annotation work of the RELISH consortium. All annotations submitted by participants before 27 July 2018 were consolidated into the initial revision of RELISH-DB.

The remainder of this section presents statistics of database content and consortium participation, and the results for annotation consistency analyses and method performance evaluations.

### Annotations were contributed by a diverse group of highly experienced original authors

**Figure 2:**
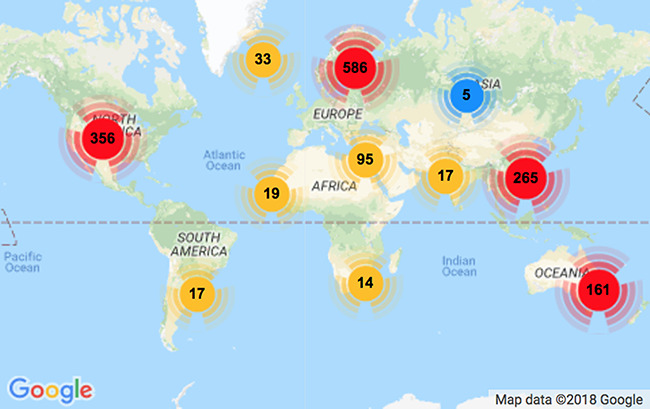
Distribution of participant geolocation. There are 1570 registered participants from 84 unique countries. The majority of contributors are from Europe, North America, China and Australia.

Table 1 shows simple statistics of RELISH-DB content according to participants’ research experience. Annotations were received from a total of 1643 unique scientists around the world, of whom 1570 are registered participants (affiliated consortium members) and 73 contributed anonymously. Figure 2 shows that the contributors to this project originated from diverse geographic locations, including 84 unique countries with the largest clusters located in Europe (586), North America (356), China (265) and Oceania (161). Altogether, the participants annotated 3017 seed articles or 181,020 (3017$\times $60) labelled document pairs. The average number of submissions per participant was 1.9 (all anonymous submissions were considered as individuals). The majority of participants (77%) submitted annotations for just one seed article, 11% for two, 3% for three and 9% for four or more, respectively. A few dedicated participants annotated more than 50 seed articles. The majority of contributions (63%) were made by experienced researchers with 5 or more years’ experience post-PhD. More significantly, the majority of seed articles (91%) were evaluated by one of the original authors according to name matches. These statistics suggest high-quality annotations within RELISH-DB.

**Table 1:** Distribution of database content by participant experience level. Registered and anonymous columns show the number of participants at respective experience levels. Contributions is the total number of seed articles evaluated by participants at respective experience levels.

	Registered	Anonymous	Contributions
PhD (>10 years)	652 (41.5%)	34 (46.6%)	1391 (46.1%)
PhD (5–10 years)	249 (15.9%)	7 (9.6%)	512 (17.0%)
PhD (<5 years)	283 (18.0%)	14 (19.2%)	538 (17.8%)
PhD (student)	191 (12.2%)	11 (15.1%)	282 (9.3%)
Other	195 (12.4%)	7 (9.6%)	294 (9.7%)
Total	**1570**	**73**	**3017**

Note: contribution total includes both registered and anonymous submissions.

### Annotations covered diverse research areas

**Figure 3:**
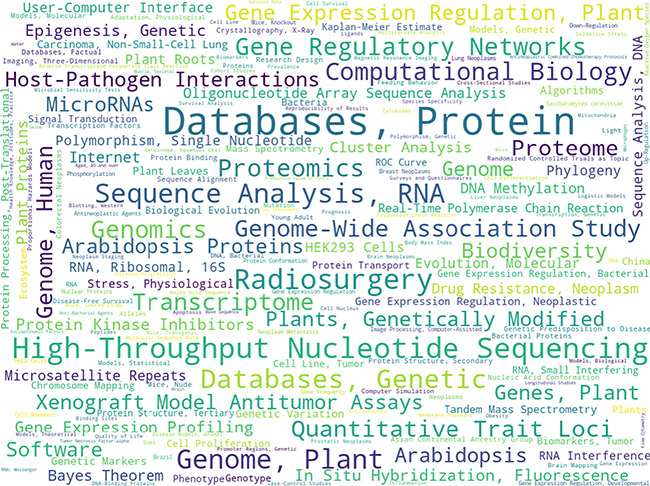
Word cloud of the 250 most frequent MeSH qualifiers from all seed articles normalized by frequencies in the whole PubMed library. In total, unique qualifiers present in the seed article collection (i.e. each seed article with its recommended candidate articles) cover 76% of all unique MeSH qualifiers.

Figure 3 shows a word-cloud distribution of MeSH descriptor frequencies for all annotated documents, normalized by baseline frequencies in all PubMed documents. There seems to be a slight over-representation of publications on high-throughput ‘Omics’ technologies (e.g. genomics, genome-wide association study, nucleotide sequencing, proteomics). It is possible that researchers in the field of bioinformatics/computational biology were particularly eager to participate in the study of annotating documents as they are probably the ones seeing the most benefit in having a large annotated dataset of high quality. It may also be due to the first participant recruitment phase via internal referrals and personal invitations were somewhat enriched with scientists working in the bioinformatics/computational biology areas. On the other hand, it could simply reflect the general trend in biomedical research of using more and more ‘Omics’ technologies. Nevertheless, the diversity of annotated content was illustrated by a 76% coverage of all unique MeSH descriptors in the official PubMed library collection by all documents within RELISH-DB (every seed article plus every candidate article per seed article). Such a diversity in research fields is important for benchmarking literature-searching tools applicable to all biomedical research fields.

### Partial relevance was the most popular annotation

**Figure 4:**
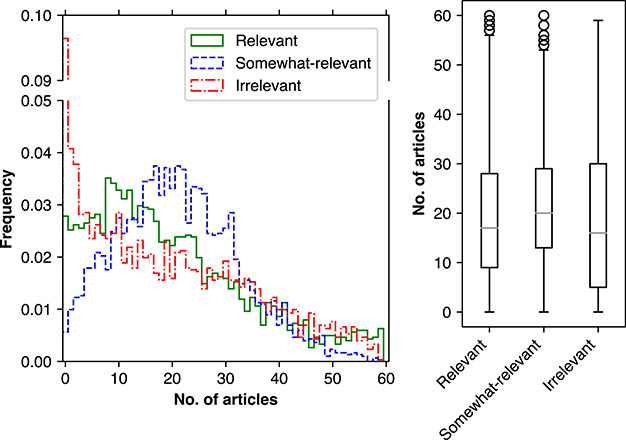
Left: frequency of seed articles (y-axis) containing $n$ annotated candidate articles of respective labels (x-axis). Right: distribution of annotated candidate articles across all seed articles. Boxes represent the quartiles; middle lines are the median values.

The distribution of the three possible relevance labels (relevant, somewhat-relevant and irrelevant) across all contributions per seed article is shown in Figure 4. The chart on the left indicates the frequency of seed articles with $n$ candidate articles in respective relevance labels. It should be noted here that the frequency refers to the proportion of all 3017 seed articles, and the sum of frequencies in each column is not 1. For example, considering the column at zero number of articles, it should be interpreted that for relevant, 2.7% or 84 seed articles have no candidate articles marked as relevant. Summing 2.7%, 0.5% and 9.7% for respective relevance labels means that 12.9% or 392 seed articles in total have no candidate articles in one of the relevance labels. The peaks for relevant, somewhat-relevant and irrelevant annotations was at 8 (106 articles, 3.5%), 17 (113 articles, 3.7%) and 0 (291 articles, 9.7%), respectively. These distribution peaks suggest that many articles fell into the grey area of partial relevance. Additionally, the preference for partial relevance can be observed from the box plot on the right side of Figure 4 where the median number of somewhat-relevant candidate articles per seed article is 20, compared to 17 for relevant and 16 for irrelevant. Furthermore, when considering the average and standard deviation of candidate articles per seed article in each relevance label, somewhat-relevant had the highest average and the lowest deviation of 21 ± 11, compared to 19 ± 14 for relevant and 19 ± 15 for irrelevant. The overall dominance of relevant and somewhat-relevant annotations suggests the reasonable performance of the three baseline methods in filtering out obviously irrelevant articles.

### Consistency according to method performance

Our definition of relevance, partial relevance and irrelevance is subject to different interpretations by different individuals. The perceived efficacy of document recommendation systems depends on the expected agreement between the subjective opinions of different individuals. To examine if such agreement exists, we compared the performance of the three baseline methods on annotations made by scientists of different experience levels, annotations of the same articles by different individuals, annotations across different research fields and durations of time spent annotating.

To carry out these comparisons, seed articles for the benchmark datasets were selected from RELISH-DB according to the following conditions. First, we removed 44 seed articles that PMRA provided fewer than 20 article recommendations for; this was to ensure a fair comparison with the other baseline methods that will always have 20 recommendations per seed article. Next, 186 seed articles without any positive or negative samples were removed as assessment of discrimination is impossible when all candidate articles belong to either the positive or negative class. Finally, we set aside 154 duplicate annotations of the same seed article by different participants (‘D154’), and 400 randomly selected seed articles as an independent test set (‘T400’), to be analyzed separately.

This led to a total of 2233 seed articles (‘ALL2233’) available for evaluation. From this set we derived an ‘NR’ (non-redundant) set, comprised of a single seed article from each unique participant to avoid potential bias against specific annotators. In this dataset, for participants with multiple seed article contributions, a seed article was selected at random. This ‘NR’ set had 1220 seed articles (‘NR1220’) in total, less than the total number of unique participants due to aforementioned exclusions.

Here, we have collectively defined candidate articles marked as ‘somewhat-relevant’ or ‘irrelevant’ as negative samples, with those marked as ‘relevant’ defined as positive samples. This was to challenge the three baseline methods and allow the measurement of their performance according to binary classification. For all statistical testing we used the Wilcoxon signed-rank test (75) implemented by the SciPy (38) library.

### Consistency between all and non-redundant sets

Table 2 compares the performance of the three baseline methods within the ‘ALL2233’ and ‘NR1220’ sets. The table shows that the three methods all had quite similar performances, with TF-IDF having the slightest edge in all metrics except precision @ 20. Moreover, the method performance trend was shared between the ‘ALL2233’ and ‘NR1220’ sets, suggesting that participants with multiple seed article annotations have not detectably biased the overall result. This is likely due to the majority of all unique participants submitting annotations for a single seed article only. Another interesting observation is that PMRA produced the most candidate articles marked as relevant (indicated by average precision @ 20), but seemed to have difficulty ranking these extra positive samples towards the top of the result list.

**Table 2:** Overall evaluation set performance results for the PMRA, BM25 and TF-IDF methods. ‘ALL’ includes all seed articles from all participants, and ‘NR’ includes only one seed article from each participant, corresponding to the ‘ALL2233’ and ‘NR1220’ sets, respectively. Performance was measured using MCC, AUC (ROC), MRR and P@N, given as (mean ± stdev).

		#	MCC	AUC	MRR	P@5	P@10	P@20
ALL	PMRA	2233	0.494 ± 0.23	0.675 ± 0.19	0.850 ± 0.28	0.614 ± 0.31	0.538 ± 0.29	**0.464 ± 0.27**
	BM25		0.509 ± 0.22	0.687 ± 0.18	0.859 ± 0.28	0.622 ± 0.30	0.536 ± 0.28	0.457 ± 0.27
	TFIDF		**0.510 ± 0.22**	**0.688 ± 0.18**	**0.873 ± 0.27**	**0.634 ± 0.30**	**0.541 ± 0.28**	0.463 ± 0.27
NR	PMRA	1220	0.479 ± 0.22	0.664 ± 0.19	0.868 ± 0.27	0.639 ± 0.29	0.565 ± 0.28	**0.492 ± 0.27**
	BM25		0.498 ± 0.22	0.675 ± 0.18	0.878 ± 0.26	0.642 ± 0.29	0.559 ± 0.27	0.481 ± 0.26
	TFIDF		**0.511 ± 0.22**	**0.685 ± 0.18**	**0.893 ± 0.25**	**0.662 ± 0.29**	**0.566 ± 0.28**	0.488 ± 0.27

The bold values indicate maximal metric value between the three baseline methods.

**Table 3:** Wilcoxon signed-rank test *P*-values between the PMRA, BM25 and TF-IDF methods for the overall evaluation set. ‘ALL’ includes all seed articles from all participants, and ‘NR’ includes only one seed article from each participant, corresponding to the ‘ALL2233’ and ‘NR1220’ sets, respectively. Metrics include MCC, AUC (ROC), MRR and P@N.

				MCC	AUC	MRR	P@5	P@10	P@20
ALL	PMRA	vs	BM25	**0.00700**	**0.02137**	0.15841	0.07316	0.65928	0.07093
	PMRA	vs	TFIDF	**0.00727**	**0.01686**	**0.00074**	**0.00012**	0.18130	0.71967
	BM25	vs	TFIDF	0.84588	0.83891	**0.01023**	**0.00912**	**0.04617**	**0.01502**
NR	PMRA	vs	BM25	**0.01985**	0.11524	0.23883	0.41124	0.54669	**0.04575**
	PMRA	vs	TFIDF	**0.00015**	**0.00263**	**0.01043**	**0.00121**	0.33197	0.81799
	BM25	vs	TFIDF	0.08311	0.08005	0.06478	**0.00134**	0.05845	0.05030

The bold values indicate *P*-values <0.05.

Statistical *P*-values from Wilcoxon signed-rank tests are given in Table 3. In both the ‘ALL2233’ and ‘NR1220’ sets, TF-IDF was significantly higher than PMRA with *P*-values less than 0.05 for MCC, AUC, MRR and P@5. For the ‘ALL2233’ set, BM25 was also significantly higher than PMRA in terms of MCC and AUC; however, in the ‘NR1220’ only MCC is significant. In all cases there was no significant difference in either MCC or AUC between BM25 and TF-IDF. However TF-IDF did significantly outperform BM25 in terms of P@5. These results suggest that out of the three baseline methods, TF-IDF was most effective at ranking relevant candidate articles highly.

### Consistency between author and non-author annotators

As previously mentioned, according to author name matches, the majority (91%) of our annotated seed articles were provided by one of the respective authors. Here we investigate whether method performance is affected considering annotations given by non-authors. These non-author annotators may have more general knowledge and not be biased by a close-up view of their given field like an author could be, potentially providing a more objective assessment.

A total of 181 seed articles were identified to have been annotated by a non-author (anonymous annotations were not included), submitted by 73 unique annotators. These articles were split into respective ‘ALL’ and ‘NR’ subsets accordingly. Table 4 shows method performances, which we are comparing to results presented in Table 2. A differing trend in method preference can be observed within the ‘ALL’ set; however, measured performance and deviation of performance between methods is comparable. Within the ‘NR’ set, both method performance and preference is almost identical, although according to the precision measures the methods seem to have fared slightly better. Overall, these results suggest that there is no significant difference between annotations contributed by annotators who authored the seed article versus external annotators who did not author the seed article.

**Table 4:** Non-author annotator evaluation set performance results for the PMRA, BM25 and TF-IDF methods. ‘ALL’ includes all seed articles in the set, and ‘NR’ includes only one seed article from each unique annotator in the set. Performance was measured using MCC, AUC (ROC), MRR and P@N, given as (mean ± stdev).

		#	MCC	AUC	MRR	P@5	P@10	P@20
ALL	PMRA	181	0.477 ± 0.20	**0.670 ± 0.16**	**0.878 ± 0.25**	**0.676 ± 0.27**	**0.599 ± 0.25**	0.530 ± 0.25
	BM25		**0.482 ± 0.22**	0.670 ± 0.18	0.857 ± 0.28	0.663 ± 0.30	0.593 ± 0.29	0.524 ± 0.27
	TFIDF		0.468 ± 0.22	0.655 ± 0.19	0.876 ± 0.26	0.676 ± 0.30	0.598 ± 0.29	**0.539 ± 0.29**
NR	PMRA	73	0.469 ± 0.19	0.666 ± 0.17	0.872 ± 0.26	0.671 ± 0.26	0.595 ± 0.24	0.516 ± 0.25
	BM25		0.495 ± 0.21	0.675 ± 0.18	0.884 ± 0.27	0.707 ± 0.30	0.619 ± 0.29	0.540 ± 0.27
	TFIDF		**0.501 ± 0.21**	**0.684 ± 0.18**	**0.902 ± 0.24**	**0.712 ± 0.30**	**0.632 ± 0.29**	**0.553 ± 0.29**

The bold values indicate maximal metric value between the three baseline methods.

**Table 5:** Detailed performance results of the ‘NR1220’ evaluation set broken down by participant experience levels for the PMRA,BM25 and TF-IDF methods. Metrics include MCC, AUC (ROC), MRR and P@N, given as (mean ± stdev).

		#	MCC	AUC	MRR	P@5	P@10	P@20
PhD (>10 years)	PMRA	473	0.483 ± 0.22	0.663 ± 0.19	0.891 ± 0.26	0.649 ± 0.30	0.575 ± 0.28	**0.505 ± 0.28**
	BM25		**0.509 ± 0.23**	**0.684 ± 0.19**	0.885 ± 0.25	0.657 ± 0.29	0.578 ± 0.28	0.501 ± 0.27
	TFIDF		0.505 ± 0.23	0.684 ± 0.18	**0.894 ± 0.24**	**0.671 ± 0.29**	**0.580 ± 0.28**	0.503 ± 0.28
PhD (5–10 years)	PMRA	189	0.493 ± 0.23	0.670 ± 0.20	0.855 ± 0.27	0.637 ± 0.29	**0.574 ± 0.28**	**0.499 ± 0.26**
	BM25		0.495 ± 0.22	0.670 ± 0.19	0.862 ± 0.27	0.640 ± 0.29	0.561 ± 0.27	0.484 ± 0.25
	TFIDF		**0.513 ± 0.23**	**0.687 ± 0.19**	**0.880 ± 0.24**	**0.661 ± 0.27**	0.565 ± 0.28	0.488 ± 0.27
PhD (<5 years)	PMRA	192	0.473 ± 0.23	0.666 ± 0.19	0.860 ± 0.28	0.655 ± 0.30	0.579 ± 0.29	0.506 ± 0.27
	BM25		0.491 ± 0.21	0.677 ± 0.17	0.899 ± 0.24	0.662 ± 0.29	0.578 ± 0.27	0.506 ± 0.26
	TFIDF		**0.519 ± 0.22**	**0.683 ± 0.19**	**0.924 ± 0.22**	**0.694 ± 0.29**	**0.585 ± 0.27**	**0.515 ± 0.27**
PhD (student)	PMRA	188	0.479 ± 0.22	0.669 ± 0.17	0.857 ± 0.26	0.620 ± 0.28	0.545 ± 0.26	**0.470 ± 0.26**
	BM25		0.485 ± 0.19	0.659 ± 0.17	**0.894 ± 0.24**	0.629 ± 0.29	0.535 ± 0.27	0.452 ± 0.25
	TFIDF		**0.504 ± 0.21**	**0.683 ± 0.18**	0.887 ± 0.26	**0.644 ± 0.29**	**0.552 ± 0.27**	0.468 ± 0.25
Other	PMRA	178	0.464 ± 0.21	0.651 ± 0.18	0.842 ± 0.28	0.616 ± 0.28	**0.532 ± 0.27**	**0.459 ± 0.25**
	BM25		0.496 ± 0.23	0.671 ± 0.19	0.841 ± 0.29	0.597 ± 0.29	0.510 ± 0.27	0.433 ± 0.25
	TFIDF		**0.523 ± 0.20**	**0.693 ± 0.18**	**0.874 ± 0.26**	**0.627 ± 0.28**	0.525 ± 0.27	0.439 ± 0.25

The bold values indicate maximal metric value between the three baseline methods.

### Consistency among different experience levels

Table 5 shows method performance on the ‘NR1220’ set broken down by annotator experience level. Similarly to the overall result discussed in the previous section, all of the baseline methods are again very close in performance, with TF-IDF performing the best within each experience group in terms of MCC, AUC, MRR and P@5 (except for MCC and AUC in the ‘PhD-[>10y]’ group, and MRR in the ‘PhD-[student]’ group, where BM25 is best). Consistently with the overall result, it can also be seen here that PMRA produced the most candidate articles marked as relevant by having the highest P@20 for each experience level except for the ‘PhD-[<5y]’ group.

**Figure 5:**
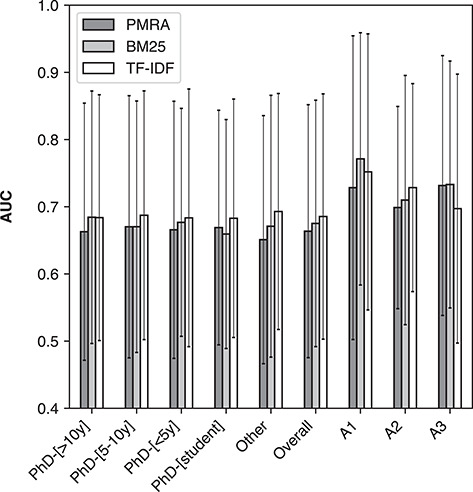
Method performance in terms of average AUC (ROC) for PMRA, BM25 and TF-IDF on the ‘NR1220’ set across experience levels within annotators and across three individual annotators (A1, A2 and A3). Error bars represent standard deviation.

Figure 5 shows average AUC values given by PMRA, BM25 and TF-IDF across annotators in different levels of research experience (also shown are individual annotator groups: A1, A2 and A3; see the following section). Here the error bars indicate standard deviation. No significant difference was observed between AUC values of the three methods among different levels of experience. This is echoed by *P*-values of the distribution comparison using Wilcoxon signed-rank tests presented in Table 6; unlike the overall result, there are not many statistically significant differences in performance between methods within the experience groups.

**Table 6:** Wilcoxon signed-rank test *P*-values between the PMRA, BM25 and TF-IDF methods for the ‘NR1220’ evaluation set broken down by participant experience level. Metrics include MCC, AUC (ROC), MRR and P@N.

				MCC	AUC	MRR	P@5	P@10	P@20
PhD (>10 years)	PMRA	vs	BM25	0.09947	0.08577	0.73131	0.26733	0.49185	0.80459
	PMRA	vs	TFIDF	0.14961	0.08633	0.80955	**0.04125**	0.41442	0.95105
	BM25	vs	TFIDF	0.91288	0.91259	0.46470	0.15842	0.59832	0.31508
PhD (5–10 years)	PMRA	vs	BM25	0.91613	0.96776	0.75115	0.57120	0.62401	0.26477
	PMRA	vs	TFIDF	0.67782	0.75515	0.35483	0.22400	0.93127	0.42470
	BM25	vs	TFIDF	0.63916	0.49584	0.34198	0.24906	0.52570	0.82280
PhD (<5 years)	PMRA	vs	BM25	0.44472	0.78528	0.07519	0.78676	0.97772	0.71899
	PMRA	vs	TFIDF	**0.01568**	0.11687	**0.01056**	**0.03357**	0.66985	0.31110
	BM25	vs	TFIDF	0.16426	0.66017	0.17176	**0.00921**	0.88387	0.48275
PhD (student)	PMRA	vs	BM25	0.30311	0.87772	0.07136	0.35153	0.31363	0.14597
	PMRA	vs	TFIDF	0.10552	0.26117	0.22179	0.21592	0.47528	0.99855
	BM25	vs	TFIDF	0.48173	0.08897	0.66555	0.85557	0.11509	0.05989
Other	PMRA	vs	BM25	0.12823	0.21695	0.98643	0.15593	0.30027	**0.01592**
	PMRA	vs	TFIDF	**0.00165**	0.05235	0.23290	0.73095	0.92710	0.30452
	BM25	vs	TFIDF	**0.03934**	0.10300	0.13172	**0.03291**	0.13125	0.60436

The bold values indicate *P*-values <0.05.

Finally, although the overall average was nearly identical among annotators with different experience levels, fluctuations around the average (standard deviations around 0.2) were quite substantial. This large fluctuation, however, may not have been caused by individuals’ subjective opinions. This is explored further with the assessment of single participants who annotated many seed articles in the next section.

### Consistency within individual annotators

As some dedicated contributors had annotated a large number of seed articles, we were able to assess method performance within these groups. Table 7 presents the results for the three largest groups, ‘A1’, ‘A2’ and ‘A3’ that had 92, 55 and 49 seed articles, respectively. In fact, these groups appeared to also exhibit a similar level of fluctuation as observed in the results for both the overall and divided by experience sets. Thus, it is hypothesized that the observed fluctuation could have been a result of a small number of candidate article samples (20) per seed article per method for calculating the various measures or it could be attributed to true differences in the ability to rank candidate articles, i.e. that the three methods rank candidate articles better for some seed articles than others.

**Table 7:** Performance results of the three largest single-annotator sets for the PMRA, BM25 and TF-IDF methods. Metrics include MCC, AUC (ROC), MRR and P@N, given as (mean ± stdev).

		#	MCC	AUC	MRR	P@5	P@10	P@20
A1	BM25	92	**0.610 ± 0.25**	**0.771 ± 0.19**	**0.740 ± 0.34**	0.335 ± 0.19	**0.226 ± 0.13**	0.150 ± 0.09
	PMRA		0.580 ± 0.26	0.728 ± 0.23	0.726 ± 0.35	0.313 ± 0.19	0.212 ± 0.11	0.149 ± 0.08
	TFIDF		0.601 ± 0.27	0.752 ± 0.21	0.735 ± 0.35	**0.337 ± 0.23**	0.220 ± 0.13	**0.155 ± 0.09**
A2	BM25	55	0.538 ± 0.23	0.710 ± 0.19	0.882 ± 0.24	0.611 ± 0.29	**0.498 ± 0.29**	**0.408 ± 0.27**
	PMRA		0.547 ± 0.17	0.699 ± 0.15	0.892 ± 0.24	0.553 ± 0.28	0.420 ± 0.24	0.342 ± 0.22
	TFIDF		**0.564 ± 0.20**	**0.729 ± 0.15**	**0.900 ± 0.25**	**0.615 ± 0.30**	0.480 ± 0.28	0.377 ± 0.26
A3	BM25	49	0.550 ± 0.25	**0.733 ± 0.18**	**0.966 ± 0.14**	0.780 ± 0.22	0.698 ± 0.26	**0.627 ± 0.28**
	PMRA		**0.561 ± 0.25**	0.732 ± 0.19	0.944 ± 0.17	**0.788 ± 0.24**	**0.718 ± 0.24**	0.614 ± 0.28
	TFIDF		0.504 ± 0.25	0.697 ± 0.20	0.922 ± 0.20	0.710 ± 0.26	0.659 ± 0.29	0.605 ± 0.31

The bold values indicate maximal metric value between the three baseline methods.

**Figure 6:**
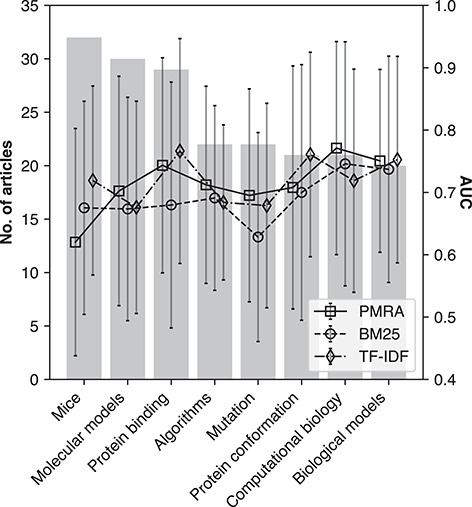
Method performance in terms of AUC (ROC) for PMRA, BM25 and TF-IDF across eight different research areas: Mice (‘D051379’), Molecular models (‘D008958’), Protein binding (‘D011485’), Algorithms (‘D000465’), Mutation (‘D009154’), Protein conformation (‘D011487’), Computational biology (‘D019295’) and Biological models (‘D008954’). Bars correspond to number of seed articles in the topic area, whereas lines indicate method performance according to AUC values. Error bars represent standard deviation.

Additionally, as method performance is again quite close overall, some noise appeared to exist regarding the preferred method within these individual annotator groups. It could be argued that ‘A1’ prefers BM25, ‘A2’ prefers TF-IDF and ‘A3’ prefers PMRA. Furthermore, precision-based metrics for ‘A1’ demonstrated fluctuation of ‘difficulty’, compared to the ‘A2’ and ‘A3’ groups, with regard to the number of ‘positive’ annotations per seed article. For example, a P@20 of around 0.1 means that on average each method was trying to rank 2 relevant articles higher than 18 irrelevant articles that must have also had large content-based similarity of some form allowing their initial detection by the baseline methods. In contrast, ‘A2’ and ‘A3’ were relatively easier, especially considering the MRR value close to 1, meaning that on average the top result was actually relevant.

### Consistency among different research fields

Here, we assessed performance consistency across different research topics, defined by MeSH descriptors associated with each seed article (if any). Articles that shared a MeSH descriptor were clustered accordingly. The eight largest clusters were evaluated, Figure 6 shows method AUC performance within these clusters (lines and error bars of standard deviation corresponding to right y-axis), and the number of seed articles within each cluster (bars corresponding to left y-axis). The identified topics include Mice (‘D051379’), Molecular Models (‘D008958’), Protein Binding (‘D011485’), Algorithms (‘D000465’), Mutation (‘D009154’), Protein Conformation (‘D011487’), Computational Biology (‘D019295’) and Biological Models (‘D008954’). Each topic was represented fairly equally, where the range of seed articles per topic was between 20 and 30. No significant performance deviation was apparent among the research topics.

### Consistency of annotation for articles labelled by multiple annotators

A more direct assessment of consistency is to examine the same document pairs annotated by different contributors, using the ‘D154’ set. These contributions covered a total of 74 unique articles. However, 32 articles were removed due to duplicate submitted annotations from a single participant (i.e. the same participant contributed annotations more than once for the same seed article). Subsequently excluded were two articles that were annotated by three participants. In total, 40 unique articles remained in this consistency set (80 of 154 contributions; each article here was annotated by exactly two unique participants).

**Figure 7:**
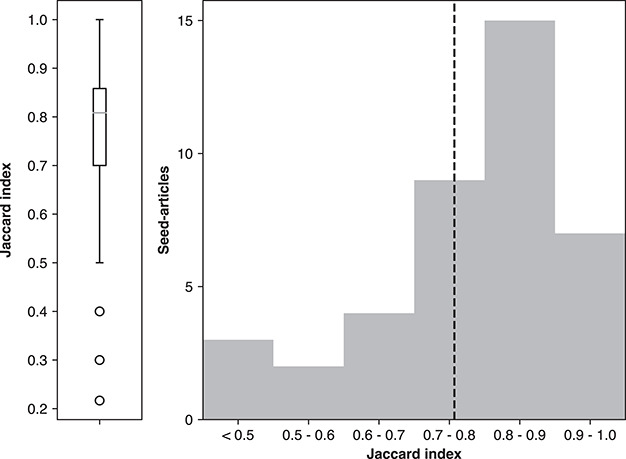
Agreement between different annotators for the same seed article using Jaccard Index shown as average (left) and distribution (right). The box on the left represents quartiles with the median as the middle line. The dashed line on the right is the average agreement value.

We measured the agreement by first collapsing ternary class annotations into their binary class equivalents. Next the Jaccard index (48) was calculated, defined as the intersection size of the same positive annotations plus the intersection size of the same negative annotations divided by the union size of all annotations. The Jaccard index distribution can be seen in Figure 7, where the overall trend was found to indicate strong consistency, having only 3 out of the 40 cases below 50% agreement. The average annotation consistency was 75%. In one of the low-agreement cases, we contacted the original contributors and found that one annotator defined relevance by ‘AND’ (satisfaction of all topic keywords), whereas the other by ‘OR’ (as long as it contained one essential topic keyword). Nevertheless, there was high consistency observed among the majority of dual-annotated document pairs.

### Consistency regarding duration of time spent for annotations

The duration of time a participant spent annotating seed articles was an additional aspect we tested for consistency. This duration was estimated from the time of loading the first page of results to the time of submission. As this time was estimated and is likely to contain a small degree of error due to noise in the logs, we restricted the duration of time spent to between 2 and 120 min.

**Figure 8:**
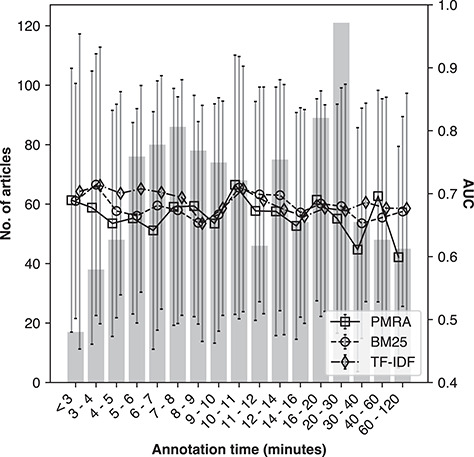
Method performance as a function of annotation time spent for the ‘NR1220’ evaluation set; the x-axis is binned time-spent in minutes; the vertical bars corresponding to the left y-axis is the number of seed articles per bin; the lines corresponding to the right y-axis show average AUC (ROC) per bin for the PMRA, BM25 and TF-IDF methods, respectively.

Figure 8 demonstrates method performance as a function of time spent on annotations for the ‘NR1220’ set. Bin sizes (bars corresponding to left y-axis) and method performances (lines and error bars of standard deviation with square, circle and diamond markers for PMRA, BM25 and TF-IDF, respectively corresponding to right y-axis) are indicated. As the majority of annotations were submitted within 17 min (75%), to equalize bin size as much as possible, bin intervals had to be varied across the x-axis (they are not uniformly sized). There was no obvious association observed between time spent on annotations and the resultant method performance, indicating that there was no detectable systematic bias underlying time spent for annotations.

### Consistency among thresholds for binary classification

Distinguishing positives from negatives requires a threshold for the similarity score calculated by PMRA, BM25 or TF-IDF. An ideal method should have the same threshold for different seed articles regardless of research field and abstract/title length. This would be beneficial to real-world recommender systems by enabling a dynamic number of articles deemed as relevant to be presented to the user rather than the traditional approach of delivering a fixed number of recommendations.

**Figure 9:**
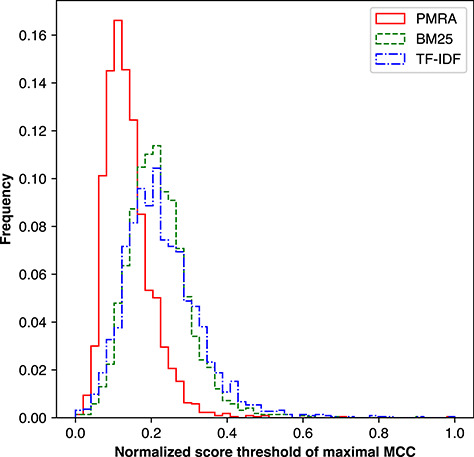
Distribution of score thresholds yielding the highest MCC value per seed article for the PMRA, BM25 and TF-IDF methods.

We analyzed the distribution of method score thresholds in Figure 9. Raw method scores were first normalized using the min-max method and then divided into 50 equally sized bins. All of the methods’ threshold distributions were found to be relatively centralized around their respective peaks; however, PMRA clearly compared well to other methods and had the most stable cut-off values with a standard deviation of 0.06, compared to 0.08 and 0.09 for BM25 and TF-IDF, respectively. This finding suggests the possibility of setting a unified relevance cut-off.

### Differences between the three methods

**Figure 10:**
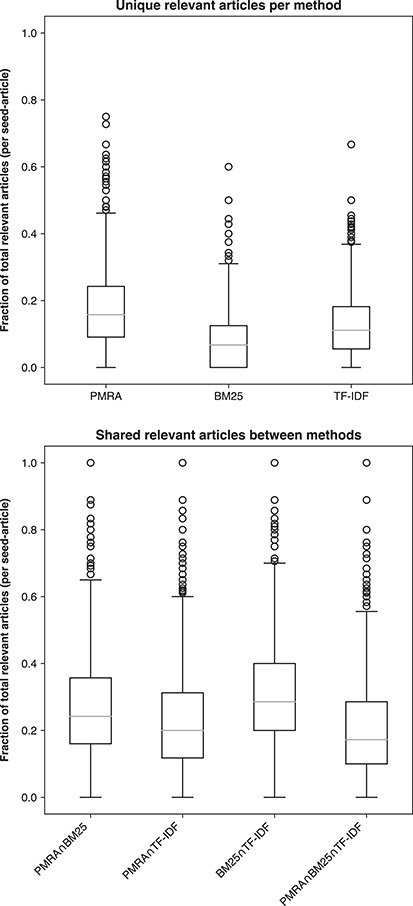
Consistency and difference among three methods: PMRA, BM25 and TF-IDF; upper—unique relevant recommendations given by each method; lower—overlap of relevant recommendations between methods. Boxes represent the quartiles; middle lines are the median.

Figure 10 examines true relevant articles uniquely contributed by each method (PMRA, BM25 or TF-IDF in the top panel) and the overlaps among them. In most cases PMRA output the largest proportion of relevant articles (avg. 17%) that were not given by either BM25 or TF-IDF (additionally highlighted by the average P@20 for PMRA). In contrast, BM25 had the lowest contribution of unique relevant recommendations (avg. 8%). Meanwhile, the overlap among all methods was low with the highest overlap between BM25 and TF-IDF at 32% on average. Thus, clearly none of these baseline methods could provide complete coverage of all relevant articles. Relatively few recommendations were shared by the three methods, indicating that is likely possible to establish a well-performing hybrid method that would aggregate outputs of multiple methods.

## Data Records

A complete dump of collected annotation data as at 27 July 2018 was deposited to a figshare repository. The dataset is released without copyright, available at https://figshare.com/projects/RELISH-DB/60095, under the CC0 license. All data records were stripped of personally identifiable information and then converted to JSON format (41). Record fields include a unique identifier (‘uid’), PubMedID of the seed article (‘pmi’), annotator experience level (‘experience’), whether the annotator was an anonymous or registered user (‘is_anonymous’) and annotator response (‘response’) containing the lists of candidate article PubMedIDs corresponding to the assigned degree of relevance (i.e. one of ‘relevant’, ‘partial’ (somewhat-relevant) or ‘irrelevant’).

## Usage Notes

To make RELISH collections useful for future method developers, we have established a data server at https://relishdb.ict.griffith.edu.au. This data server has three modules for data annotation, data retrieval and method testing.

**Figure 11:**
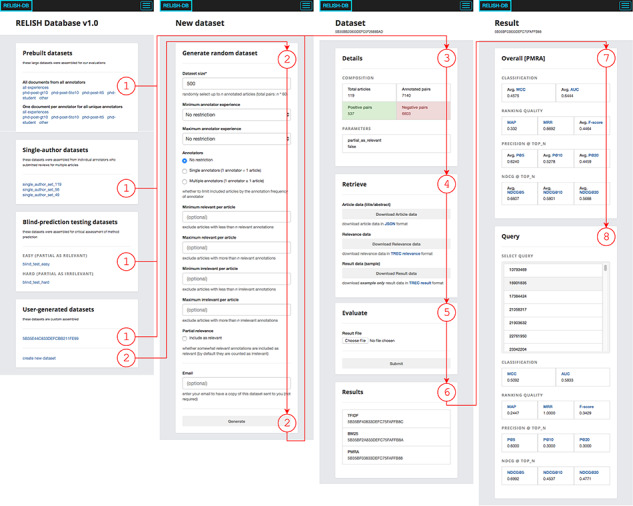
An overview of the database retrieval and evaluation process: (1) use existing datasets—both pre-built and user-generated; (2) create custom datasets—allows for user-defined dataset construction by tuning dataset generation parameters; (3) dataset details—shows the size, number of positive and negative pairs and any custom parameters used to generate; (4) dataset article data—allows user download of (a) raw article metadata (id, title and abstract) (b) annotation data and (c) sample result file for evaluation function; (5) result file upload—allows input of result files for automatic performance assessment; (6) uploaded evaluations—results of uploaded result file evaluations for the respective dataset will be presented here; (7) overall evaluation view—provides a performance summary of the dataset as a whole; (8) detailed evaluation view—performance details are broken down on a per-query basis.

**Data annotation:** data annotation functions exactly as the APSE server. As shown in Figure 1, users can search for publications related to any article of interest, with the option to voluntarily annotate article relevance. New submissions will be automatically saved for inclusion in the next database version.
**Data retrieval:** we have made available several pre-built datasets (the evaluation sets within this work) on our data server. These include the complete versions of the ‘ALL2233’ and ‘NR1220’ sets, as well as copies of these sets broken down by annotator experience level. In addition, the three single-annotator sets (‘A1’, ‘A2’ and ‘A3’) are also available. Furthermore, we also provided an option to allow users to generate a dataset according to various pre-defined parameters (dataset size, experience level, single seed article or multiple seed article annotators and cut-offs for the number of candidate articles labelled as relevant or irrelevant per seed article).
  As shown in Figure 11, pre-compiled datasets can be inspected by starting at (1). Alternatively, user-defined datasets may be generated by starting at (2). After generating a new dataset, or clicking on an existing one, the dataset view page will be displayed as shown in stage (3). Here, dataset details including size, number of positive and negative pairs, and any custom parameters that were set will be given. For each dataset, we make available the respective subsets of article metadata, annotation data and an example result file in the format the automatic evaluation will accept, as shown in stage (4). The metadata includes the PubMedID, title and abstract for each article involved in the dataset. Because we are using publicly licensed PubMed metadata, we allow for direct download of this metadata in JSON format (41). Relevance data are provided in TREC relevance format (56). Method developers can divide downloaded datasets into training and independent test sets according to their own specific needs.
**Method testing:** to facilitate comparison and avoid overtraining, we set aside a blind-test set for critical assessment of method performance. This selection of 400 randomly selected NR seed articles have their annotation data withheld from the rest of the database. The dataset has two variants (‘BT1’ where partially relevant articles are considered as ‘positive’ samples, whereas in ‘BT2’ partially relevant articles are considered as ‘negative’ samples). Baseline method performances for this set are shown in Table 8. Overall, the results are consistent with what was observed for the complete dataset. We envision that method developers can download pre-built or user-defined datasets for training and independent tests. Subsequently, they can download ‘BT1’ or ‘BT2’ to provide additional tests by submitting the result file in TREC result format (61) as shown in stage (5) of Figure 11. Evaluations will be automatically executed and a list of recorded evaluations for the dataset will be shown in stage (6). After uploading a result file for a new evaluation, or clicking on an existing evaluation in the list, the evaluation results will be displayed: firstly, a summary of metrics over the dataset as a whole is given by stage (7) and secondly, the metrics are broken down into a per seed article query basis for inspection in stage (8).

**Table 8:** Performance results of the blind test set of 400 randomly selected articles for the PMRA, BM25 and TF-IDF methods. ‘BT1’ considered partially relevant articles as ‘positive’, and ‘BT2’ considered partially relevant articles as ‘negative’. Metrics include MCC, AUC (ROC), MRR and P@N, given as (mean ± stdev).

		MCC	AUC	MRR	P@5	P@10	P@20
BT1	BM25	0.489 ± 0.25	0.680 ± 0.20	0.964 ± 0.15	0.857 ± 0.20	0.807 ± 0.20	0.748 ± 0.20
	PMRA	0.497 ± 0.24	0.694 ± 0.19	0.967 ± 0.13	0.871 ± 0.19	**0.828 ± 0.19**	**0.763 ± 0.19**
	TFIDF	**0.512 ± 0.24**	**0.699 ± 0.19**	**0.981 ± 0.10**	**0.875 ± 0.18**	0.821 ± 0.19	0.759 ± 0.19
BT2	BM25	0.495 ± 0.19	0.665 ± 0.17	0.856 ± 0.29	0.582 ± 0.30	0.482 ± 0.25	0.396 ± 0.21
	PMRA	**0.497 ± 0.20**	**0.676 ± 0.17**	0.853 ± 0.28	**0.605 ± 0.29**	**0.506 ± 0.25**	**0.413 ± 0.21**
	TFIDF	0.493 ± 0.19	0.670 ± 0.16	**0.877 ± 0.26**	0.594 ± 0.27	0.492 ± 0.23	0.404 ± 0.21

The bold values indicate maximal metric value between the three baseline methods.

## Motivation and Limitations

Many users, both expert and non-expert, require literature searching for various purposes. Generally, the information need revolves around knowledge expansion of a topic; whether the goal is to find pertinent supporting material to solidify insight or to broaden their vision by locating different aspects, of a particular claim, idea or field of research.

Users most commonly rely on manual keyword searching methods to serve their needs (19, 21, 31, 37, 70). While it is recognized that this type of searching tools are indispensable (19); a major concern, one that the use case of our work attempts to address, is how users can be easily dissatisfied by the results given by this procedure. For example, produced results largely depend on, and may substantially vary, based on how well the user is able to ‘fine-tune’ their queries delivered to the searching system (31). Moreover, the conversion process between an information need to an effective query is often a potential source of difficulty (19, 37). While search engines often offer ‘advanced’ operators for more elaborate queries, the user unfriendliness of these syntaxes diminish their use to a negligible rate (69). Ultimately, users’ needs are met in a ‘hit-or-miss’ basis (2, 36, 69) within this approach.

Here, the use case of our work falls into the ‘recommendation’ sub-field of literature search. Specifically, we focus on pairwise similarity detection between documents. Considering the rate of literature growth and the associated ‘information overload’ for users, remedial approaches like recommender systems have been proposed to tackle the difficulty and time burden of keeping track of the most promising and relevant studies (12, 30). While at the present time recommender systems may be considered an uncommon use case for biomedical literature search systems, such as PubMed, there has been interest shown in the development of additional biomedical searching tools (53), including systems that allow identification of similar publications (18, 20, 26, 60, 76). Moreover, the frequency of their use depends strongly on the accuracy of recommendations. It is the developers of these types of recommendation systems we anticipate to benefit the most from our database.

This work is not the first to propose the use of a seed article (manuscript) as a query instead of keywords (10, 27, 37, 46, 55, 71). As stated in (31), the following scenarios illustrate potential applications of a system built upon this ideology: editors or reviewers wishing to explore a subject matter of which is not their specialty (e.g. to find other potential peer reviewers), researchers simply wishing to find related studies to a paper of interest (e.g. for the purpose of locating citations or venues for their work), researchers who are pursuing a new area of which they have little existing knowledge (e.g. a student following up on a paper given by their supervisor) and researchers wishing to keep up with latest developments in their field based on their previous publications.

Finally, we must stress that the basis of our use case here is a complementary alternative to keyword search, it is not intended as a replacement. An accurate keyword-less recommendation system is sure to benefit both expert and non-expert users, especially in this era of information overload. In addition, this is more than a database exclusively for seed article-based search. It can be used to train keywords or sentence-based search by examining if keywords or the title from one article would automatically lead to the other articles. That is, a method can be trained for title/sentence-based and title/abstract based search on this benchmark.

## Discussion

Our work represents a community-wide effort to establish a database of document relevance that is suitable for machine learning. It should be noted that our APSE system facilitating the annotation process was not intended as a stand-alone search engine. Rather, it was developed and employed exclusively for data collection. Nevertheless, using three different methods appeared to provide a more complete search compared to systems powered by a single technique such as TF-IDF in JANE (67) or PMRA in PubMed (49). This is consistent with comments from many users who found interesting articles previously unknown to them.

More than 1500 scientists around the world participated in the RELISH consortium and annotated the relevance for over 180,000 document pairs that represent diverse research fields, covering 76% of all PubMed MeSH descriptors without clear bias (Figure 3). These annotations are of high quality as more than 90% of the article pairs were annotated by original authors and 63% by scientists with 5 or more years research experience post-PhD. While the majority annotated a single seed article, a few dedicated researchers annotated more than 50 seed articles, and some independently annotated the same seed articles. Together, the resulting dataset provides the largest manually annotated benchmark for detecting document similarity in biomedical literature search.

A number of potential systematic biases were considered during the construction of this database. Potentially affected aspects of our methodology primarily include how the documents presented to annotators were selected and how annotators themselves were selected. We discuss here the biases we were able to identify and explain the rationale behind how they were dealt with.

Inherent selection biases could be argued regarding the baseline methods used to generate the sets of documents for annotation. Firstly, since only the top 20 non-redundant documents from each method were presented to annotators, we have necessarily excluded potentially related articles with lower similarity scores from this pool. We could have taken more results from a single method, but then bias towards that single method would have been introduced. Additionally, we chose to take this number of results from each method to reduce the burden on annotator’s participation time, as we appreciate that volunteered time to contribute is valuable. Secondly, as the baseline methods rely exclusively on exact term matches to generate respective similarity scores, it is possible that semantically related documents are missing from this pool. Although there was no scalable solution present to circumvent this, we feel it should be mentioned here.

Potentially subjective bias is a major concern affecting document relevance manually annotated by such a diverse group of scientists. This could be in terms of a biased consideration of recommended documents according to the annotator’s close-up view of their given field as opposed to the more objective consideration annotators with general knowledge could have. We opted to use authors of their own papers as relevance judges to increase participation rate, the effort required from participants was again considered here. This is because our goal for this benchmark was to capture sub-field similarity such that articles marked as relevant could have been suitable for citation by the originating seed article. This task requires highly specialized experts in respective sub-fields to make that determination. In other words, the more sub-field experience of an annotator, the more accurate annotations will be made. Although non-PhD researchers are underrepresented in their participation of database construction, it will not affect the overall accuracy of annotations as we have demonstrated that there is no bias according to the experience level of annotators and across different fields of researchers. In fact, using expert annotations will ensure the production of relevant articles for all non-PhD researchers, clinicians and the general public in their searches.

If a subjective bias exists, one would expect that the performance of the three baseline methods (PMRA, BM25 and TF-IDF) would have certain systematic trends. We showed that method performances were nearly the same across different annotator experience levels (Figure 5), different research topics (Figure 6) and different durations of time spent annotating (Figure 8) and whether annotators evaluated articles they authored or not (Tables 2 and 4). Although the magnitude of the performance fluctuation was large between different annotators, the same magnitude of fluctuation was observed within individual annotators (Figure 5), suggesting that the source of fluctuation likely was small size of samples (20 per seed article for each method) and/or true differences in difficulty (i.e. some seed articles are easier to find relevant articles for than others). More importantly, examining same document–document pairs annotated by different researchers indicated a high average consistency of 75% (Figure 7). Furthermore, high consistency in the dataset was also suggested by the relatively narrow distribution of thresholds that were used to maximize the MCC for each seed article (Figure 9). This indicated that relevance can be defined across different experience levels or research fields. The results collectively indicate that despite the presence of any subjective bias, a consensus exists regarding what is actually relevant literature for the majority of researchers, despite differences in research areas and experience levels.

High-quality data collected by the RELISH consortium offered an unprecedented opportunity to compare the three baseline methods (PMRA, BM25 and TF-IDF). Somewhat surprisingly, all three methods performed similarly, with TF-IDF presenting a slight edge. Our finding is somewhat different from previous studies (22, 51, 68) where the classic TF-IDF was often considered inferior to methods developed afterwards. This result highlights the importance of having a large benchmark dataset. Moreover, all three methods evaluated here performed only moderately well with an average AUC of around 0.7 and average MCC of around 0.5. In addition, different methods did capture different sets of relevant articles (Figure 10). This further indicates the necessity of large benchmark evaluation datasets, significant room exists for improving literature search with future method development.

Encouraging method development was the principal motivation behind the assembly of this database. A recent survey of research paper recommendation systems revealed the urgent need for a common evaluation framework (4), as current method evaluation results are rarely reproducible (3). This work was inspired by large human-annotated databases like ImageNet (17), where labelled images have promoted method development and helped to revolutionize object detection and image classification (45). Our database should allow novel methods to be developed based on modern deep machine learning techniques to address the major deficiencies of current methods. Additionally, the benchmark datasets as a result of our database will enable objective comparison between old and new methods, increasing the uptake of newer methods and their translation into practice. To assist unbiased evaluation, we have made the RELISH database freely accessible to the academic community (released under the CC0 license). Furthermore, we set aside $400 \times 60$ document–document pairs for blind prediction using the automatic evaluation by our database server at https://relishdb.ict.griffith.edu.au.

We expect that dissemination of this manuscript will attract more scientists to contribute more annotations. A major struggle for us was participant recruitment, the first phase of our recruitment strategy (internal referrals, personal invitations, social media posts and a correspondence letter) ultimately failed to attract enough contributions to establish a meaningful dataset. Because of this failure we reluctantly decided to proceed with the second phase of sending direct emails. Although the response rate was low (around 1%), it was good enough to achieve the sizeable database we have now. Our server will continue to collect annotations. We hope to publish the next version of the database with at least double the number of present annotations.

## Conflict of interest

None declared.

## Author contributions

P.B. participated in the design, carried out the study, implemented the websites and search systems and wrote the manuscript. RELISH consortium annotated the articles. Y.Z. conceived the study, participated in the initial design, assisted in analyzing data and wrote the manuscript. All authors read, contributed to the discussion and approved the manuscript.

### RELISH consortium members

Aik-Choon Tan^3^,

Mohamed A. El-Esawi^4^,

Thomas Liehr^5^,

Oliver Blanck^6^,

Douglas P. Gladue^7^,

Gabriel M. F. Almeida^8^,

Tomislav Cernava^9^,

Carlos O. Sorzano^10^,

Andy W. K. Yeung^11^,

Michael S. Engel^12^,

Arun Richard Chandrasekaran^13^,

Thilo Muth^14^,

Martin S. Staege^15^,

Swapna V. Daulatabad^16^,

Darius Widera^17^,

Junpeng Zhang^18^,

Adrian Meule^19^,

Ken Honjo^20^,

Olivier Pourret^21^,

Cong-Cong Yin^22^,

Zhongheng Zhang^23^,

Marco Cascella^24^,

Willy A. Flegel^25^,

Carl S. Goodyear^26^,

Mark J. van Raaij^10^,

Zuzanna Bukowy-Bieryllo^27^,

Luca G. Campana^28^,

Nicholas A. Kurniawan^29^,

David Lalaouna^30^,

Felix J. Hüttner^31^,

Brooke A. Ammerman^32^,

Felix Ehret^33^,

Paul A. Cobine^34^,

Ene-Choo Tan^35^,

Hyemin Han^36^,

Wenfeng Xia^37^,

Christopher McCrum^38^,

Ruud P. M. Dings^39^,

Francesco Marinello^40^,

Henrik Nilsson^41^,

Brett Nixon^42^,

Konstantinos Voskarides^43^,

Long Yang^44^,

Vincent D. Costa^45^,

Johan Bengtsson-Palme^46^,

William Bradshaw^47^,

Dominik G. Grimm^48^,

Nitin Kumar^49^,

Elvis Martis^50^,

Daniel Prieto^51^,

Sandeep C. Sabnis^52^,

Said E. D. R. Amer^53^,

Alan W. C. Liew^1^,

Paul Perco^54^,

Farid Rahimi^55^,

Giuseppe Riva^56^,

Chongxing Zhang^57^,

Hari P. Devkota^58^,

Koichi Ogami^59^,

Zarrin Basharat^60^,

Walter Fierz^61^,

Robert Siebers^62^,

Kok-Hian Tan^63^,

Karen A. Boehme^64^,

Peter Brenneisen^65^,

James A. L. Brown^66^,

Brian P. Dalrymple^67^,

David J. Harvey^68^,

Grace Ng^69^,

Sebastiaan Werten^70^,

Mark Bleackley^71^,

Zhanwu Dai^72^,

Raman Dhariwal^73^,

Yael Gelfer^74^,

Marcus D. Hartmann^75^,

Pawel Miotla^76^,

Radu Tamaian^77^,

Pragashnie Govender^78^,

Oliver J. Gurney-Champion^79^,

Joonas H. Kauppila^80^,

Xiaolei Zhang^81^,

Natalia Echeverría^82^,

Santhilal Subhash^83^,

Hannes Sallmon^84^,

Marco Tofani^85^,

Taeok Bae^86^,

Oliver Bosch^87^,

Páraic O. Cuív^88^,

Antoine Danchin^89^,

Barthelemy Diouf^90^,

Tuomas Eerola^91^,

Evangelos Evangelou^92^,

Fabian V. Filipp^93^,

Hannes Klump^94^,

Lukasz Kurgan^95^,

Simon S. Smith^96^,

Olivier Terrier^97^,

Neil Tuttle^98^,

David B. Ascher^99^,

Sarath C. Janga^100^,

Leon N. Schulte^101^,

Daniel Becker^102^,

Christopher Browngardt^103^,

Stephen J. Bush^68^,

Guillaume Gaullier^104^,

Kazuki Ide^105^,

Clement Meseko^106^,

Gijsbert D. A. Werner^107^,

Jan Zaucha^108^,

Abd A. Al-Farha^109^,

Noah F. Greenwald^110^,

Segun I. Popoola^111^,

Md Shaifur Rahman^112^,

Jialin Xu^113^,

Sunny Y. Yang^113^,

Noboru Hiroi^114^,

Ozgul M. Alper^115^,

Chris I. Baker^116^,

Michael Bitzer^117^,

George Chacko^118^,

Birgit Debrabant^119^,

Ray Dixon^120^,

Evelyne Forano^72^,

Matthew Gilliham^121^,

Sarah Kelly^122^,

Karl-Heinz Klempnauer^123^,

Brett A. Lidbury^124^,

Michael Z. Lin^125^,

Iseult Lynch^126^,

Wujun Ma^127^,

Edward W. Maibach^128^,

Diane E. Mather^129^,

Kutty S. Nandakumar^130^,

Robert S. Ohgami^131^,

Piero Parchi^132^,

Patrizio Tressoldi^133^,

Yu Xue^134^,

Charles Armitage^135^,

Pierre Barraud^136^,

Stella Chatzitheochari^137^,

Luis P. Coelho^138^,

Jiajie Diao^139^,

Andrew C. Doxey^140^,

Angélique Gobet^141^,

Pingzhao Hu^142^,

Stefan Kaiser^143^,

Kate M. Mitchell^144^,

Mohamed F. Salama^145^,

Ivan G. Shabalin^146^,

Haijun Song^147^,

Dejan Stevanovic^148^,

Ali Yadollahpour^149^,

Erliang Zeng^150^,

Katharina Zinke^151^,

C. G. Alimba^152^,

Tariku J. Beyene^153^,

Zehong Cao^154^,

Sherwin S. Chan^155^,

Michael Gatchell^156^,

Andreas Kleppe^157^,

Marcin Piotrowski^158^,

Gonzalo Torga^159^,

Adugna A. Woldesemayat^160^,

Mehmet I. Cosacak^161^,

Scott Haston^162^,

Stephanie A. Ross^163^,

Richard Williams^164^,

Alvin Wong^165^,

Matthew K. Abramowitz^166^,

Andem Effiong^167^,

Senhong Lee^168^,

Muhammad Bilal Abid^169^,

Cyrus Agarabi^170^,

Cedric Alaux^370^,

Dirk R. Albrecht^171^,

Gerald J. Atkins^172^,

Charles R. Beck^173^,

A. M. J. J. Bonvin^174^,

Emer Bourke^175^,

Thomas Brand^176^,

Ralf J. Braun^177^,

James A. Bull^178^,

Pedro Cardoso^179^,

Dee Carter^180^,

Robin M. Delahay^181^,

Bernard Ducommun^182^,

Pascal H. G. Duijf^183^,

Trevor Epp^184^,

Eeva-Liisa Eskelinen^185^,

Mazyar Fallah^186^,

Debora B. Farber^187^,

Jose Fernandez-Triana^188^,

Frank Feyerabend^189^,

Tullio Florio^190^,

Michael Friebe^191^,

Saori Furuta^192^,

Mads Gabrielsen^193^,

Jens Gruber^194^,

Malgorzata Grybos^195^,

Qian Han^196^,

Michael Heinrich^197^,

Heikki Helanterä^198^,

Michael Huber^199^,

Albert Jeltsch^200^,

Fan Jiang^201^,

Claire Josse^202^,

Giuseppe Jurman^203^,

Haruyuki Kamiya^204^,

Kim de Keersmaecker^205^,

Erik Kristiansson^206^,

Frank-Erik de Leeuw^207^,

Jiuyong Li^208^,

Shide Liang^209^,

Jose A. Lopez-Escamez^210^,

Francisco J. Lopez-Ruiz^211^,

Kevin J. Marchbank^212^,

Rolf Marschalek^213^,

Carmen S. Martín^10^,

Adriana E. Miele^214^,

Xavier Montagutelli^215^,

Esteban Morcillo^216^,

Rosario Nicoletti^217^,

Monika Niehof^218^,

Ronan O’Toole^219^,

Toshihiko Ohtomo^220^,

Henrik Oster^221^,

Jose-Alberto Palma^222^,

Russell Paterson^223^,

Mark Peifer^224^,

Maribel Portilla^7^,

M. C. Portillo^225^,

Antonia L. Pritchard^226^,

Stefan Pusch^227^,

Gajendra P. S. Raghava^228^,

Nicola J. Roberts^229^,

Kehinde Ross^230^,

Birgitt Schuele^231^,

Kjell Sergeant^232^,

Jun Shen^233^,

Alessandro Stella^234^,

Olga Sukocheva^235^,

Vladimir N. Uversky^236^,

Sven Vanneste^237^,

Martin H. Villet^238^,

Miguel Viveiros^239^,

Julia A. Vorholt^240^,

Christof Weinstock^241^,

Masayuki Yamato^242^,

Ioannis Zabetakis^243^,

Xin Zhao^244^,

Andreas Ziegler^245^,

Wan M. Aizat^246^,

Lauren Atlas^247^,

Kristina M. Bridges^248^,

Sayan Chakraborty^249^,

Mieke Deschodt^250^,

Helena S. Domingues^251^,

Shabnam S. Esfahlani^252^,

Sebastian Falk^253^,

J. L. Guisado^254^,

Nolan C. Kane^255^,

Gray Kueberuwa^256^,

Colleen L. Lau^257^,

Dai Liang^258^,

Enwu Liu^259^,

Andreas M. Luu^260^,

Chuang Ma^261^,

Lisong Ma^262^,

Robert Moyer^263^,

Adam D. Norris^264^,

Suresh Panthee^265^,

Jerod R. Parsons^266^,

Yousong Peng^267^,

Inês Mendes Pinto^251^,

Cristina R. Reschke^269^,

Elina Sillanpää^270^,

Christopher J. Stewart^212^,

Florian Uhle^271^,

Hui Yang^272^,

Kai Zhou^273^,

Shu Zhu^274^,

Mohamed Ashry^275^,

Niels Bergsland^276^,

Maximilian Berthold^277^,

Chang-Er Chen^278^,

Vito Colella^279^,

Maarten Cuypers^280^,

Evan A. Eskew^281^,

Xiao Fan^282^,

Maksymilian Gajda^283^,

Rayner Gonzálezlez-Prendes^284^,

Amie Goodin^285^,

Emily B. Graham^286^,

Ewout J. N. Groen^287^,

Alba Gutiérrez-Sacristán^288^,

Mohamad Habes^289^,

Enrico Heffler^290^,

Daniel B. Higginbottom^291^,

Thijs Janzen^292^,

Jayakumar Jayaraman^293^,

Lindsay A. Jibb^294^,

Stefan Jongen^295^,

Timothy Kinyanjui^296^,

Rositsa G. Koleva-Kolarova^297^,

Zhixiu Li^298^,

Yu-Peng Liu^299^,

Bjarte A. Lund^300^,

Alexandre A. Lussier^301^,

Liping Ma^302^,

Pablo Mier^303^,

Matthew D. Moore^304^,

Katja Nagler^305^,

Mark W. Orme^306^,

James A. Pearson^307^,

Anilkumar S. Prajapati^308^,

Yu Saito^309^,

Simon E. Tröder^310^,

Florence Uchendu^311^,

Niklas Verloh^312^,

Denitza D. Voutchkova^313^,

Ahmed Abu-Zaid^314^,

Joaira Bakkach^315^,

Philipp Baumert^316^,

Marcos Dono^317^,

Jack Hanson^318^,

Sandrine Herbelet^319^,

Emma Hobbs^320^,

Ameya Kulkarni^321^,

Narendra Kumar^322^,

Siqi Liu^323^,

Nikolai D. Loft^324^,

Tristan Reddan^325^,

Thomas Senghore^326^,

Howard Vindin^327^,

Haotian Xu^328^,

Ross Bannon^329^,

Branson Chen^330^,

Johnny T. K. Cheung^331^,

Jeffrey Cooper^332^,

Ashwini K. Esnakula^333^,

Karine A. Feghali^334^,

Emilia Ghelardi^335^,

Agostino Gnasso^336^,

Jeffrey Horbar^337^,

Hei M. Lai^89^,

Jian Li^338^,

Lan Ma^339^,

Ruiyan Ma^340^,

Zihang Pan^341^,

Marco A. Peres^342^,

Raymond Pranata^343^,

Esmond Seow^344^,

Matthew Sydes^345^,

Ines Testoni^346^,

Anna L. Westermair^347^,

Yongliang Yang^348^,

Masoud Afnan^349^,

Joan Albiol^350^,

Lucia G. Albuquerque^351^,

Eisuke Amiya^353^,

Rogerio M. Amorim^354^,

Qianli An^355^,

Stig U. Andersen^356^,

John D. Aplin^357^,

Christos Argyropoulos^358^,

Yan W. Asmann^359^,

Abdulaziz M. Assaeed^360^,

Atanas G. Atanasov^361^,

David A. Atchison^362^,

Simon V. Avery^363^,

Paul Avillach^288^,

Peter D. Baade^364^,

Lars Backman^365^,

Christophe Badie^366^,

Alfonso Baldi^367^,

Elizabeth Ball^368^,

Olivier Bardot^369^,

Adrian G. Barnett^371^,

Mathias Basner^372^,

Jyotsna Batra^298^,

O. M. Bazanova^373^,

Andrew Beale^374^,

Travis Beddoe^375^,

Melanie L. Bell^376^,

Eugene Berezikov^377^,

Sue Berners-Price^2^,

Peter Bernhardt^378^,

Edward Berry^379^,

Theolis B. Bessa^380^,

Craig Billington^381^,

John Birch^382^,

Randy D. Blakely^383^,

Mark A. T. Blaskovich^384^,

Robert Blum^385^,

Marleen Boelaert^386^,

Dimitrios Bogdanos^387^,

Carles Bosch^388^,

Thierry Bourgoin^389^,

Daniel Bouvard^390^,

Laura M. Boykin^391^,

Graeme Bradley^392^,

Daniel Braun^393^,

Jeremy Brownlie^394^,

Albert Brühl^395^,

Austin Burt^396^,

Lisa M. Butler^397^,

Siddappa N. Byrareddy^398^,

Hugh J. Byrne^399^,

Stephanie Cabantous^400^,

Sara Calatayud^216^,

Eva Candal^401^,

Kimberly Carlson^402^,

Sònia Casillas^403^,

Valter Castelvetro^404^,

Patrick T. Caswell^405^,

Giacomo Cavalli^406^,

Vaclav Cerovsky^407^,

Monica Chagoyen^408^,

Chang-Shi Chen^409^,

Dong F. Chen^410^,

Hao Chen^411^,

Hui Chen^412^,

Jui-Tung Chen^413^,

Yinglong Chen^414^,

Changxiu Cheng^415^,

Jianlin Cheng^416^,

Mai Chinapaw^417^,

Christos Chinopoulos^418^,

William C. S. Cho^419^,

Lillian Chong^420^,

Debashish Chowdhury^421^,

Andre Chwalibog^422^,

A. Ciresi^423^,

Shamshad Cockcroft^424^,

Ana Conesa^425^,

Penny A. Cook^426^,

David N. Cooper^427^,

Olivier Coqueret^428^,

Enoka M. Corea^429^,

Elisio Costa^431^,

Carol Coupland^432^,

Stephanie Y. Crawford^433^,

Aparecido D. Cruz^434^,

Huijuan Cui^435^,

Qiang Cui^436^,

David C. Culver^437^,

Amedeo D’Angiulli^438^,

Tanya E. S. Dahms^439^,

France Daigle^440^,

Raymond Dalgleish^441^,

Håvard E. Danielsen^157^,

Sébastien Darras^443^,

Sean M. Davidson^444^,

David A. Day^445^,

Volkan Degirmenci^446^,

Luc Demaison^72^,

Koenraad Devriendt^447^,

Jiandong Ding^448^,

Yunus Dogan^449^,

X. C. Dong^450^,

Claudio F. Donner^451^,

Walter Dressick^452^,

Christian A. Drevon^453^,

Huiling Duan^454^,

Christian Ducho^455^,

Nicolas Dumaz^456^,

Bilikere S. Dwarakanath^457^,

Mark H. Ebell^458^,

Steffen Eisenhardt^459^,

Naser Elkum^460^,

Nadja Engel^461^,

Timothy B. Erickson^462^,

Michael Fairhead^47^,

Marty J. Faville^463^,

Marlena S. Fejzo^464^,

Fernanda Festa^465^,

Antonio Feteira^466^,

Patrick Flood-Page^467^,

John Forsayeth^468^,

Simon A. Fox^469^,

Steven J. Franks^470^,

Francesca D. Frentiu^135^,

Mikko J. Frilander^471^,

Xinmiao Fu^472^,

Satoshi Fujita^473^,

Ian Galea^474^,

Luca Galluzzi^475^,

Federica Gani^476^,

Arvind P. Ganpule^477^,

Antonio García-Alix^478^,

Kristene Gedye^479^,

Maurizio Giordano^480^,

Cecilia Giunta^481^,

Paul A. Gleeson^482^,

Cyrille Goarant^483^,

Haipeng Gong^484^,

Diop Gora^485^,

Michael J. Gough^486^,

Ravinder Goyal^487^,

Kathryn E. Graham^488^,

Ana Grande-Pérez^489^,

Patricia M. Graves^490^,

Harm Greidanus^491^,

Darren Grice^2^,

Christoph Grunau^492^,

Yosephine Gumulya^493^,

Yabin Guo^494^,

Vsevolod V. Gurevich^495^,

Oleg Gusev^496^,

Elke Hacker^298^,

Steffen R. Hage^497^,

Guy Hagen^498^,

Steven Hahn^499^,

Dagmar M. Haller^500^,

Sven Hammerschmidt^501^,

Jianwei Han^502^,

Renzhi Han^503^,

Martin Handfield^504^,

Hapuarachchige C. Hapuarachchi^505^,

Timm Harder^506^,

Jennifer E. Hardingham^507^,

Michelle Heck^508^,

Marcel Heers^509^,

Khe F. Hew^510^,

Yohei Higuchi^511^,

Cynthia St. Hilaire^512^,

Rachel Hilton^513^,

Enisa Hodzic^514^,

Andrew Hone^515^,

Yuichi Hongoh^516^,

Guoku Hu^398^,

Heinz P. Huber^517^,

Luis E. Hueso^518^,

Judith Huirne^519^,

Lisa Hurt^520^,

Helena Idborg^521^,

Kazuho Ikeo^522^,

Evan Ingley^523^,

Philip M. Jakeman^524^,

Arne Jensen^525^,

Hong Jia^526^,

Husen Jia^2^,

Shuqin Jia^527^,

Jianping Jiang^528^,

Xingyu Jiang^529^,

Yi Jin^530^,

Daehyun Jo^531^,

Andrew M. Johnson^532^,

Marie Johnston^533^,

Karen R. Jonscher^534^,

Philippe G. Jorens^535^,

Jens O. L. Jorgensen^536^,

Johan W. Joubert^537^,

Sin-Ho Jung^538^,

Antonio M. Junior^539^,

Thomas Kahan^540^,

Sunjeev K. Kamboj^541^,

Yong-Kook Kang^542^,

Yannis Karamanos^543^,

Natasha A. Karp^544^,

Ryan Kelly^545^,

Ralph Kenna^546^,

Jonathan Kennedy^547^,

Birgit Kersten^548^,

Roy A. Khalaf^549^,

Javaria M. Khalid^550^,

T. Khatlani^551^,

Tarig Khider^552^,

Gregor S. Kijanka^553^,

Sarah R. B. King^554^,

Tomasz Kluz^555^,

Paul Knox^556^,

Tatsuya Kobayashi^557^,

Karl-Wilhelm Koch^558^,

Maija R. J. Kohonen-Corish^559^,

Xiangpeng Kong^560^,

Deborah Konkle-Parker^561^,

Kalevi M. Korpela^562^,

Leondios G. Kostrikis^563^,

Peter Kraiczy^564^,

Harald Kratz^565^,

Günter Krause^566^,

Paul H. Krebsbach^567^,

Søren R. Kristensen^568^,

Prerna Kumari^569^,

Akira Kunimatsu^570^,

Hatice Kurdak^571^,

Young D. Kwon^572^,

Carl Lachat^573^,

Malgorzata Lagisz^574^,

Brenda Laky^575^,

Jan Lammerding^576^,

Matthias Lange^577^,

Mar Larrosa^578^,

Andrew L. Laslett^579^,

Elizabeth E. LeClair^581^,

Kyung-Woo Lee^582^,

Ming-Yih Lee^583^,

Moon-Soo Lee^584^,

Genyuan Li^585^,

Jiansheng Li^586^,

Klaus Lieb^587^,

Yau Y. Lim^588^,

Merry L. Lindsey^589^,

Paul-Dag Line^590^,

Dengcai Liu^591^,

Fengbin Liu^592^,

Haiyan Liu^274^,

Hongde Liu^593^,

Vett K. Lloyd^594^,

Te-Wen Lo^595^,

Emanuela Locci^596^,

Josef Loidl^597^,

Johan Lorenzen^598^,

Stefan Lorkowski^599^,

Nigel H. Lovell^600^,

Hua Lu^601^,

Wei Lu^602^,

Zhiyong Lu^603^,

Gustavo S. Luengo^604^,

Lars-Gunnar Lundh^605^,

Philippe A. Lysy^606^,

Angela Mabb^607^,

Heather G. Mack^608^,

David A. Mackey^609^,

S. R. Mahdavi^610^,

Pamela Maher^611^,

Toby Maher^612^,

Sankar N. Maity^613^,

Brigitte Malgrange^614^,

Charalampos Mamoulakis^615^,

Arduino A. Mangoni^616^,

Thomas Manke^617^,

Antony S. R. Manstead^618^,

Athanasios Mantalaris^619^,

Jan Marsal^620^,

Hanns-Ulrich Marschall^621^,

Francis L. Martin^622^,

Jose Martinez-Raga^623^,

Encarnacion Martinez-Salas^624^,

Daniel Mathieu^625^,

Yoichi Matsui^626^,

Elie Maza^627^,

James E. McCutcheon^628^,

Gareth J. McKay^629^,

Brian McMillan^630^,

Nigel McMillan^631^,

Catherine Meads^632^,

Loreta Medina^633^,

B. Alex Merrick^634^,

Dennis W. Metzger^635^,

Frederic A. Meunier^636^,

Martin Michaelis^637^,

Olivier Micheau^638^,

Hisaaki Mihara^639^,

Eric M. Mintz^640^,

Takuo Mizukami^641^,

Yann Moalic^642^,

D. P. Mohapatra^643^,

Antonia Monteiro^644^,

Matthieu Montes^645^,

John V. Moran^646^,

Sergey Y. Morozov^647^,

Matthew Mort^427^,

Noriyuki Murai^648^,

Denis J. Murphy^649^,

Susan K. Murphy^650^,

Shauna A. Murray^651^,

Shinji Naganawa^652^,

Srinivas Nammi^653^,

Grigorios Nasios^654^,

Roman M. Natoli^655^,

Frederique Nguyen^656^,

Christine Nicol^657^,

Filip van Nieuwerburgh^658^,

Erlend B. Nilsen^659^,

Clarissa J. Nobile^660^,

Margaret O’Mahony^661^,

Sophie Ohlsson^662^,

Oluremi Olatunbosun^663^,

Per Olofsson^664^,

Alberto Ortiz^665^,

Kostya Ostrikov^666^,

Siegmar Otto^667^,

Tiago F. Outeiro^668^,

Songying Ouyang^472^,

Sabrina Paganoni^669^,

Andrew Page^670^,

Christoph Palm^671^,

Yin Paradies^672^,

Michael H. Parsons^470^,

Nick Parsons^673^,

Pigny Pascal^674^,

Elisabeth Paul^675^,

Michelle Peckham^676^,

Nicoletta Pedemonte^677^,

Michael A. Pellizzon^678^,

M. Petrelli^679^,

Alexander Pichugin^680^,

Carlos J. C. Pinto^681^,

John N. Plevris^682^,

Piero Pollesello^683^,

Martin Polz^684^,

Giovanna Ponti^685^,

Piero Porcelli^686^,

Martin Prince^297^,

Gwendolyn P. Quinn^687^,

Terence J. Quinn^688^,

Satu Ramula^689^,

Juri Rappsilber^690^,

Florian Rehfeldt^691^,

Jan H. Reiling^692^,

Claire Remacle^693^,

Mohsen Rezaei^694^,

Eric W. Riddick^695^,

Uwe Ritter^696^,

Neil W. Roach^697^,

David D. Roberts^698^,

Guillermo Robles^699^,

Tiago Rodrigues^700^,

Cesar Rodriguez^701^,

Jo Roislien^702^,

Monique J. Roobol^703^,

J. Alexandra Rowe^704^,

Andreas Ruepp^705^,

Jan van Ruitenbeek^706^,

Petra Rust^707^,

Sonia Saad^708^,

George H. Sack^709^,

Manuela Santos^710^,

Aurore Saudemont^711^,

Gianni Sava^712^,

Simone Schrading^713^,

Alexander Schramm^714^,

Martin Schreiber^715^,

Sidney Schuler^716^,

Joost Schymkowitz^717^,

Alexander Sczyrba^718^,

Kate L. Seib^2^,

Han-Ping Shi^719^,

Tomohiro Shimada^720^,

Jeon-Soo Shin^721^,

Colette Shortt^722^,

Patricia Silveyra^723^,

Debra Skinner^724^,

Ian Small^725^,

Paul A. M. Smeets^726^,

Po-Wah So^727^,

Francisco Solano^728^,

Daniel E. Sonenshine^729^,

Jiangning Song^730^,

Tony Southall^396^,

John R. Speakman^731^,

Mandyam V. Srinivasan^636^,

Laura P. Stabile^732^,

Andrzej Stasiak^733^,

Kathryn J. Steadman^734^,

Nils Stein^735^,

Andrew W. Stephens^736^,

Douglas I. Stewart^737^,

Keith Stine^738^,

Curt Storlazzi^739^,

Nataliya V. Stoynova^740^,

Wojciech Strzalka^741^,

Oscar M. Suarez^742^,

Taranum Sultana^743^,

Anirudha V. Sumant^744^,

Mathew J. Summers^745^,

Gang Sun^746^,

Paul Tacon^747^,

Kozo Tanaka^748^,

Haixu Tang^749^,

Yoshinori Tanino^750^,

Paul Targett-Adams^751^,

Mourad Tayebi^752^,

Reema Tayyem^753^,

Christoph C. Tebbe^754^,

Evelyn E. Telfer^755^,

Wolfram Tempel^756^,

Julita A. Teodorczyk-Injeyan^757^,

Gert Thijs^758^,

Sally Thorne^759^,

Amanda G. Thrift^760^,

Celine Tiffon^761^,

Philip Tinnefeld^762^,

Daryono H. Tjahjono^763^,

Fabrice Tolle^764^,

Ervin Toth^765^,

Andria L. del Tredici^766^,

Apostolos Tsapas^767^,

Konstantinos Tsirigotis^768^,

Ayse Turak^769^,

George Tzotzos^770^,

Edet E. Udo^771^,

Toshiaki Utsumi^772^,

Subramanian Vaidyanathan^773^,

Michel Vaillant^774^,

Armand Valsesia^775^,

Roosmarijn E. Vandenbroucke^776^,

Feliciano H. Veiga^777^,

Marc Vendrell^778^,

Peter A. Vesk^779^,

Paul Vickers^780^,

Victor M. Victor^781^,

Richard Villemur^782^,

Marie-Claude Vohl^783^,

Christian R. Voolstra^784^,

Anne Vuillemin^785^,

Steven Wakelin^786^,

Levi Waldron^787^,

Laurence J. Walsh^788^,

Amanda Y. Wang^789^,

Fuan Wang^790^,

Yun Wang^791^,

Yoichi Watanabe^792^,

Andreas Weigert^793^,

Jet-Chau Wen^794^,

Carol Wham^795^,

Ethan P. White^796^,

Jan Wiener^797^,

Gottfried Wilharm^798^,

Simon Wilkinson^799^,

Raffaella Willmann^800^,

Coralie Wilson^801^,

Brunhilde Wirth^802^,

Timothy R. Wojan^803^,

Mathieu Wolff^804^,

Bryan M. Wong^805^,

Tzu-Wei Wu^806^,

Hanno Wuerbel^807^,

Xiangshu Xiao^808^,

Dong Xu^416^,

J. W. Xu^809^,

Jianping Xu^810^,

Bin Xue^811^,

Suayib Yalcin^812^,

Hong Yan^813^,

En-Cheng Yang^814^,

Shiqi Yang^815^,

Wei Yang^816^,

Yuzhen Ye^749^,

Zhi-Qiang Ye^817^,

Jari Yli-Kauhaluoma^818^,

Hiroshi Yoneyama^819^,

Ying Yu^820^,

Guo-Cheng Yuan^821^,

Chiou-Hwa Yuh^822^,

Manuela Zaccolo^823^,

Chen Zeng^824^,

Branko Zevnik^310^,

Chi Zhang^825^,

Li Zhang^826^,

Li Zhang^827^,

Yingkai Zhang^828^,

Yusen Zhang^829^,

Zhiyong Zhang^830^,

Zhong-Yin Zhang^831^,

Yuan Zhao^832^,

Min Zhou^833^,

Torsten Zuberbier^834^,

Carmen M. Aanei^835^,

Rafi Ahmad^836^,

Manar Al-Lawama^837^,

Alexandre Alanio^838^,

Judith Allardyce^839^,

David Alonso-Caneiro^362^,

John M. Atack^2^,

Dirk Baier^840^,

Abhisheka Bansal^841^,

Yannick Benezeth^842^,

Colette Berbesque^843^,

Frederik Berrevoet^844^,

Peter H. W. Biedermann^845^,

Erik Bijleveld^846^,

Florian Bittner^847^,

Fabian Blombach^848^,

Wouter van den Bos^849^,

Shellie A. Boudreau^850^,

Adam D. Bramoweth^851^,

Oliver Braubach^852^,

Yufeng Cai^853^,

Matthew Campbell^2^,

Zanxia Cao^854^,

Thibault Catry^855^,

Xin Chen^856^,

Shuiqin Cheng^857^,

Hee-Jung Chung^858^,

Miguel A. Chávez-Fumagalli^859^,

Aaron Conway^298^,

Bruno M. Costa^860^,

Normand Cyr^861^,

Lorraine T. Dean^862^,

Martin S. Denzel^863^,

S. V. Dlamini^864^,

Kevin J. Dudley^865^,

Maeva Dufies^866^,

Thorsten Ecke^867^,

Denitsa Eckweiler^868^,

Elisenda Eixarch^869^,

Hosny El-Adawy^870^,

Julius V. Emmrich^871^,

Alex J. Eustace^872^,

Christine M. Falter-Wagner^19^,

Johannes Fuss^875^,

Jianzhao Gao^876^,

Martin R. Gill^877^,

Liz Gloyn^878^,

Robert Goggs^879^,

Usha Govinden^880^,

Garrett Greene^881^,

Victor Greiff^882^,

D. S. Grundle^883^,

Patrick Grüneberg^884^,

Nicksy Gumede^885^,

Gbaguidi Haore^886^,

Pille Harrison^887^,

Xavier Hoenner^888^,

Diego Hojsgaard^889^,

Hikaru Hori^890^,

Maria P. Ikonomopoulou^891^,

Patrick Jeurissen^892^,

Daniel M. Johnson^893^,

Dhiraj Kabra^894^,

Koji Kamagata^895^,

Chandan Karmakar^896^,

Olga Kasian^897^,

Linda K. Kaye^898^,

Murad M. Khan^899^,

Yong-Min Kim^900^,

J. K. Kish^901^,

Sebastian Kobold^902^,

Gary Kohanbash^903^,

Gregor Kohls^904^,

Jan-Michael Kugler^905^,

Gyanendra Kumar^906^,

Jon Lacy-Colson^907^,

Asam Latif^908^,

Volker M. Lauschke^909^,

Bingling Li^910^,

Chinten J. Lim^911^,

Fang Liu^912^,

Xiaodong Liu^913^,

Jin-Jian Lu^914^,

Qiang Lu^915^,

Poornima Mahavadi^916^,

Ugo Marzocchi^917^,

Christine A. McGarrigle^918^,

Tom van Meerten^919^,

Rogier Min^920^,

Iain Moal^921^,

Massimiliano Molari^922^,

Lucas Molleman^923^,

Saiful R. Mondal^924^,

Thea van de Mortel^925^,

W. N. Moss^926^,

Othonas A. Moultos^927^,

Maheswari Mukherjee^928^,

Kazuhiko Nakayama^929^,

Edward Narayan^442^,

Navaratnarajah^930^,

Philipp-Alexander Neumann^931^,

Jiyun Nie^932^,

Yingjiu Nie^933^,

Frank Niemeyer^934^,

Fiona Nolan^935^,

Ogueri Nwaiwu^936^,

Wendy H. Oldenmenger^937^,

Emmanuel Olumayede^938^,

Jianhong Ou^939^,

Menuka Pallebage-Gamarallage^940^,

Simon P. Pearce^941^,

Tuula Pelkonen^942^,

Maria C. Pelleri^132^,

Joana L. Pereira^943^,

Mpho Pheko^944^,

Karina A. Pinto^945^,

Allison Piovesan^132^,

Michael Pluess^946^,

Illya M. Podolsky^947^,

Julie Prescott^948^,

Dongchen Qi^949^,

Xingshun Qi^950^,

Vaia D. Raikou^951^,

Andreas Ranft^952^,

Johanna Rhodes^953^,

Jean-Yves Rotge^954^,

Anna D. Rowe^955^,

Manish Saggar^956^,

Robert A. Schuon^957^,

Shaouli Shahid^958^,

Vahid Shalchyan^959^,

Prasad Shirvalkar^960^,

Oleg Shiryayev^961^,

Jugpreet Singh^962^,

Michael J. Smout^963^,

António Soares^964^,

Chunjiao Song^965^,

Kshitij Srivastava^966^,

Rupesh K. Srivastava^967^,

Jim Sun^968^,

Attila Szabo^969^,

Wiktor Szymanski^970^,

Chan N. P. Tai^971^,

Hisashi Takeuchi^972^,

S. Tanadini-Lang^973^,

Fei Tang^974^,

Wanyin Tao^274^,

G. Theron^975^,

Chang F. Tian^976^,

Yu-Shi Tian^977^,

Lisa M. Tuttle^978^,

Anna Valenti^979^,

Pierre Verlot^980^,

Mirella Walker^981^,

Jun Wang^982^,

Danielle Welter^983^,

Matthew Winslade^984^,

Dalei Wu^985^,

Yi-Rui Wu^986^,

Han Xiao^987^,

Beisi Xu^988^,

Juan Xu^989^,

Ziyue Xu^990^,

Dongdong Yang^991^,

Mingjun Yang^992^,

Patricio Yankilevich^993^,

Yuyi You^994^,

Chenglong Yu^995^,

Jian Zhan^2^,

Gong Zhang^996^,

Kai Zhang^997^,

Tuo Zhang^998^,

Yi Zhang^999^,

Guoyan Zhao^1000^,

Jing Zhao^1001^,

Xiaofan Zhou^1002^,

Zhenxing Zhu^1003^,

Penelope A. Ajani^651^,

Udunna C. Anazodo^1004^,

Saeed A. Bagloee^1005^,

Kasia Bail^1006^,

Ido Bar^394^,

Joe Bathelt^1007^,

David Benkeser^1008^,

Meghan L. Bernier^1009^,

Adam M. Blanchard^1010^,

Dominic W. Boakye^1011^,

Vasileios Bonatsos^1012^,

Michele H. Boon^1013^,

George Bouboulis^1014^,

Elizabeth Bromfield^1015^,

Joshua Brown^285^,

Kim C. M. Bul^1016^,

Kathryn J. Burton^1017^,

Eugene G. Butkowski^1018^,

Grace Carroll^1019^,

Fengqing Chao^1020^,

Elisabeth E. Charrier^1021^,

Xiaoyin Chen^1022^,

Yu-Chih Chen^1023^,

Chenguang^484^,

Jane R. Choi^1024^,

Tore Christoffersen^1025^,

João C. Comel^1026^,

Cyril Cosse^1027^,

Yanru Cui^1028^,

Pieter van Dessel^1029^,

Dhaval^1030^,

Daria Diodato^1031^,

Maelle Duffey^1032^,

Avik Dutt^1033^,

Luis G. Egea^1034^,

Mohammed El-Said^1035^,

Martin Faye^1036^,

Beatriz Fernandez-Fernandez^665^,

Kieran G. Foley^1037^,

Luria L. Founou^1038^,

Fan Fu^1039^,

Rabea A. Gadelkareem^1040^,

Evgeny Galimov^1041^,

Gulcan Garip^1042^,

Alison Gemmill^1043^,

Quentin Gouil^1044^,

James Grey^1045^,

Zoya Gridneva^391^,

Michel J. Grothe^1046^,

Théophile Grébert^1047^,

Fabricio Guerrero^1048^,

Léo Guignard^1049^,

Marco J. Haenssgen^1050^,

David Hasler^1051^,

Joan Y. Holgate^1052^,

Ancheng Huang^1053^,

Amanda M. Hulse-Kemp^1054^,

Claire Jean-Quartier^1055^,

Sang-Min Jeon^1056^,

Yangyang Jia^1057^,

Catherine Jutzeler^1058^,

Panagiotis Kalatzis^1059^,

Masud Karim^208^,

Kathrin Karsay^1060^,

Anne Keitel^1061^,

Andreas Kempe^1062^,

Jeremy R. Keown^1063^,

Chin M. Khoo^1064^,

Nyil Khwaja^1065^,

Rogier A. Kievit^1007^,

Aleksandra Kosanic^784^,

Dimitrios A. Koutoukidis^1067^,

Paul Kramer^1068^,

Dilip Kumar^1069^,

Nükhet Kırağ^1070^,

Giuseppe Lanza^1071^,

Thuc D. Le^208^,

Jung W. Leem^1072^,

Daniel Leightley^1073^,

Andreia Leite^1074^,

Lukas Lercher^1075^,

Ying Li^1076^,

Renly Lim^1077^,

Luiz R. A. Lima^1078^,

Li Lin^1079^,

Tong Ling^1080^,

Yuchen Liu^1081^,

Zhonghua Liu^1082^,

Yao Lu^1083^,

Fok M. Lum^1084^,

Hang Luo^1085^,

Jatin Machhi^398^,

Angus Macleod^1086^,

Isaac Macwan^1087^,

Hanumantha R. Madala^1088^,

Nima Madani^1089^,

Nicola de Maio^921^,

Kalina Makowiecki^1090^,

Daniel J. Mallinson^1091^,

Ruta Margelyte^1092^,

Maria Caracausi^132^,

Y. Markonis^1093^,

Luca Marsili^1094^,

Suzanne Mavoa^1095^,

Lorna McWilliams^1096^,

Moa Megersa^1097^,

Caetano S. M. Mendes^1098^,

Julia Menichetti^1099^,

Rebecca Mercieca-Bebber^1100^,

Jack J. Miller^1101^,

David-Paul M. Minde^1102^,

Alexander Minges^1103^,

Eleanor Mishra^1104^,

Virendra R. Mishra^1105^,

Carly Moores^1106^,

Nicola Morrice^1107^,

Alexander E. Moskalensky^1108^,

Nicolò Navarin^1109^,

Edessa Negera^1110^,

Philippe Nolet^1111^,

Ana Nordberg^1112^,

Rickard Nordén^1113^,

Jessica P. Nowicki^1114^,

Nelly Olova^799^,

Paweł Olszewski^1115^,

Robert Onzima^1116^,

Chih-Long Pan^1117^,

Charny Park^1118^,

Dong Ik Park^1119^,

Seyoung Park^1120^,

Chandrashekhar D. Patil^1121^,

Sansoa A. Pedro^1122^,

Samuel R. Perry^2^,

Jessica Peter^1123^,

Brent M. Peterson^1124^,

Andrea Pezzuolo^40^,

Ilya Pozdnyakov^1126^,

Siyu Qian^1127^,

Lei Qin^1128^,

Ali Rafe^1129^,

Ishier Raote^1130^,

Ali Raza^1131^,

Henrike Rebl^1132^,

Osama Refai^383^,

Tim Regan^1133^,

Tambi Richa^1134^,

Mark F. Richardson^1135^,

K. R. Robinson^1136^,

Luca Rossoni^1137^,

Romain Rouet^1138^,

Soroush Safaei^1139^,

Pierre H. H. Schneeberger^1140^,

Daniela Schwotzer^1141^,

Agata Sebastian^1142^,

Jennifer Selinski^375^,

Stefanie Seltmann^1143^,

Feng Sha^1144^,

Nir Shalev^1145^,

Jin-Long Shang^1146^,

Josef Singer^1147^,

Mandeep Singh^1148^,

Taylor Smith^1149^,

Emma Solomon-Moore^1150^,

Lijuan Song^1151^,

Samuele Soraggi^1152^,

Ryan Stanley^1153^,

Nico Steckhan^1154^,

Frederic Strobl^1155^,

Lorenzo Subissi^1156^,

Irwan Supriyanto^1157^,

Chinmay R. Surve^1158^,

Tomo Suzuki^1159^,

Caitlin Syme^1160^,

Karl Sörelius^1161^,

Young Tang^1162^,

Marwa Tantawy^1163^,

Sumudu Tennakoon^1164^,

Serafino Teseo^1165^,

Christine Toelzer^1166^,

Nikola Tomov^1167^,

Miguel Tovar^1168^,

Linh Tran^1169^,

Sushil Tripathi^1170^,

Anil M. Tuladhar^207^,

Azubuike C. Ukubuiwe^1171^,

Carolina O. L. Ung^1172^,

Kaspar Valgepea^1173^,

Hamid Vatanparast^1174^,

Arnau Vidal^1175^,

Fang Wang^1176^,

Qing Wang^1177^,

Ricky Watari^1178^,

Rebecca Webster^1179^,

Ruth Webster^1180^,

Junnian Wei^1181^,

David Wibowo^1182^,

Tanja S. H. Wingenbach^1183^,

Rose M. Xavier^1184^,

Shumin Xiao^1185^,

Peng Xiong^1^,

Shicai Xu^1186^,

Shilin Xu^1187^,

Ruifeng Yao^484^,

Wen Yao^1188^,

Qinan Yin^1189^,

Yongbo Yu^1190^,

Masayoshi Zaitsu^1191^,

Zian Zeineb^1192^,

Xiao-Yong Zhan^1193^,

Jilei Zhang^1194^,

Rongqiang Zhang^1195^,

Wei Zhang^1196^,

Xianglilan Zhang^1197^,

Shan Zheng^1198^,

Bailing Zhou^1199^,

Xiaoyan Zhou^1200^,

Haroon Ahmad^2^,

Sayo A. Akinwumi^1201^,

Gregory F. Albery^1202^,

Ahmed Alhowimel^1203^,

Junaid Ali^1204^,

Mansour Alshehri^1205^,

Mohammed Alsuhaibani^1206^,

Andrey Anikin^1207^,

Samuel O. Azubuike^1208^,

Anders Bach-Mortensen^1209^,

Lior Baltiansky^1210^,

Martin Bartas^1211^,

Kiflemariam Y. Belachew^1212^,

Vivek Bhardwaj^1213^,

Karin Binder^1214^,

Nicholas S. Bland^636^,

Michael Boah^1215^,

Benjamin Bullen^1216^,

Giovanna E. Calabrò^1217^,

Tiffany J. Callahan^1218^,

Bing Cao^1219^,

Kelsey Chalmers^1220^,

Wei Chang^1221^,

Zhengping Che^1222^,

Andrew T. Y. Chen^1223^,

Haimin Chen^1224^,

Huaming Chen^233^,

Youning Chen^1225^,

Zhao Chen^1226^,

YoungRok Choi^1227^,

Mohiuddin A. K. Chowdhury^1228^,

Martin R. Christensen^1229^,

Robert S. C. Cooke^1230^,

Marzia Cottini^1231^,

Natalie V. Covington^1232^,

Catriona Cunningham^1233^,

Julien Delarocque^1234^,

Lucie Devos^1235^,

Aurup R. Dhar^1236^,

Ke-Feng Ding^1237^,

Kexian Dong^1238^,

Zheng Dong^1239^,

Niklas Dreyer^1240^,

Chelsea Ekstrand^1241^,

Tanguy Fardet^1242^,

Berhanu E. Feleke^1243^,

Thomas Feurer^1039^,

Angela Freitas^1244^,

Tian Gao^1245^,

N.G. Asefa^1246^,

Francesco Giganti^1247^,

Piotr Grabowski^690^,

José R. Guerra-Mora^1248^,

Chengying Guo^1249^,

Xinyi Guo^1250^,

Himanshu Gupta^1251^,

Shuonan He^1252^,

Marloes Heijne^1253^,

Stephanie Heinemann^1254^,

Alexander Hogrebe^1255^,

Zhengping Huang^1256^,

Sophinese Iskander-Rizk^1257^,

Lavanya M. Iyer^1258^,

Yasmin Jahan^1259^,

Ameh S. James^1260^,

Emmanuel Joel^1201^,

Bastian Joffroy^1261^,

Clara Jégousse^1262^,

George Kambondo^1263^,

Priyanka Karnati^1264^,

Cihan Kaya^1265^,

An Ke^1266^,

Daniel Kelly^1267^,

Rob Kickert^1268^,

Peter E. Kidibule^1269^,

Jennifer P. Kieselmann^1270^,

Hyeon J. Kim^1271^,

Takeshi Kitazawa^1272^,

Aniek Lamberts^1273^,

You Li^1274^,

Huakang Liang^1275^,

Sabrina N. Linn^1276^,

Thomas Litfin^1^,

Wang Liusuo^1277^,

Vasiliki Lygirou^1278^,

Ajay K. Mahato^1279^,

Zhi-Ming Mai^1280^,

Rupert W. Major^1281^,

Samira Mali^1282^,

Panagiotis Mallis^1283^,

Wenzhi Mao^484^,

Wenzhi Mao^1284^,

Katie Marvin-Dowle^1285^,

Leanda D. Mason^211^,

Ben Merideth^1286^,

Maria J. Merino-Plaza^1287^,

Britt Merlaen^1288^,

Rossella Messina^1289^,

Anand K. Mishra^1290^,

Junaid Muhammad^1291^,

Conrad Musinguzi^1292^,

Afroditi Nanou^1293^,

Amreen Naqash^1294^,

Joe T. Nguyen^1295^,

Thi T. H. Nguyen^1296^,

Duan Ni^1297^,

Nida^1298^,

Shirli Notcovich^1299^,

Barnabas Ohst^1300^,

Quinn R. Ollivier^1301^,

Daniël F. Osses^703^,

Xiangda Peng^1302^,

Arnoud Plantinga^1303^,

Michael Pulia^1304^,

Muhammad Rafiq^1305^,

Ayush Raman^1306^,

Delphine Raucher-Chéné^1307^,

Rafał Rawski^1308^,

Asit Ray^1309^,

Lubna A. Razak^1310^,

Kevin Rudolf^1311^,

Peter Rusch^1312^,

Margaux L. Sadoine^1313^,

Axel Schmidt^1314^,

Roey Schurr^1315^,

Stephen Searles^1316^,

Saurab Sharma^1317^,

Barry Sheehan^1318^,

Chunhu Shi^1319^,

Belal Shohayeb^1320^,

Andrew Sommerlad^1321^,

Jan Strehlow^1322^,

Xianbao Sun^1323^,

Raghav Sundar^1324^,

Ghazaleh Taherzadeh^1^,

Nur D. M. Tahir^1325^,

Jun Tang^1326^,

Jean Testa^1327^,

Zhiqi Tian^139^,

Qian Tingting^1328^,

Geert P. Verheijen^846^,

Casey Vickstrom^1329^,

Teng Wang^1330^,

Xiaomin Wang^1331^,

Zhenxing Wang^1332^,

Pan Wei^1333^,

Alex Wilson^1145^,

Wyart^1334^,

Abdul-Amir Yassine^1335^,

Abbas Yousefzadeh^1336^,

Asma Zare^1337^,

Zhen Zeng^1338^,

Chengrong Zhang^1339^,

Haowen Zhang^1340^,

Linxing Zhang^1341^,

Tongchuan Zhang^2^,

Weijia Zhang^208^,

Zhe Zhang^2^,

Jianyu Zhou^1342^,

Dongjie Zhu^1343^,

Vincenzo Adamo^1344^,

Adebolajo A. Adeyemo^1345^,

Maria Aggelidou^1346^,

Adi M. Al-Owaifeer^1347^,

Arwa Z. Al-Riyami^1348^,

Saeed K. Alzghari^1349^,

Vibeke Andersen^1350^,

Kathryn Angus^1351^,

Muhammad Asaduzzaman^1352^,

Hadi Asady^1353^,

Dai Ato^1354^,

Xiaoyong Bai^1355^,

Rebecca L. Baines^1356^,

Maghan Ballantyne^1357^,

Bo Ban^1358^,

Jill Beck^1359^,

Walid Ben-Nafa^426^,

Emma Black^1360^,

Antoine Blancher^1361^,

Ron Blankstein^1362^,

Neil Bodagh^1363^,

Paulo A. V. Borges^1364^,

Anastasia Brooks^1320^,

Josue Brox-Ponce^1365^,

Arturo Brunetti^1366^,

Colin D. Canham^1367^,

Piero Carninci^1368^,

Richard Carvajal^1369^,

Shun C. Chang^1370^,

Jie Chao^1371^,

Pranab Chatterjee^1372^,

He Chen^1373^,

Yi-Chun Chen^1375^,

Adnan K. Chhatriwalla^1376^,

Ibrahim Chikowe^1377^,

Trees-Juen Chuang^1378^,

Rosane G. Collevatti^1379^,

Diego A. Valera-Cornejo^1380^,

Ana Cuenda^1381^,

Myriam Dao^1382^,

Delphine Dauga^1383^,

Zaian Deng^1384^,

Kiran Devkota^1385^,

Lisa V. Doan^1386^,

Yaser H. A. Elewa^1387^,

Dongsheng Fan^1388^,

Mohammed Faruk^1389^,

Shi Feifei^1390^,

Trevor S. Ferguson^1391^,

Francesco Fleres^1392^,

Emma J. Foster^1393^,

C. Stephen Foster^1394^,

Tzvi Furer^1395^,

Yibo Gao^1396^,

Enid J. Garcia-Rivera^1397^,

Adi Gazdar^1398^,

Ronald B. George^1399^,

Sayantan Ghosh^1400^,

Elena Gianchecchi^1401^,

Joshua M. Gleason^1402^,

Allan Hackshaw^1403^,

Adam Hall^1404^,

Richard Hall^1405^,

Paul Harper^1406^,

William E. Hogg^1407^,

Guangqun Huang^1408^,

Kylie E. Hunter^1409^,

Adriaan P. IJzerman^1410^,

Carlos Jesus^1411^,

Gao Jian^1412^,

James S. Lewis Jr.^1413^,

Souha S. Kanj^1414^,

Harsheen Kaur^1415^,

Shona Kelly^1416^,

Fayez Kheir^1417^,

V. S. Kichatova^1418^,

Musa Kiyani^1419^,

Reinhild Klein^1420^,

Tom Kovesi^1421^,

Jennifer L. Kraschnewski^1422^,

Addanki P. Kumar^1423^,

Dmitry Labutin^1424^,

Alejandro Lazo-Langner^1425^,

Guy Leclercq^1426^,

Maoteng Li^1427^,

Qingchun Li^1428^,

Tangliang Li^1429^,

Yongzhe Li^1430^,

Wei-Ting Liao^1431^,

Zheng-yin Liao^1432^,

Jessica Lin^1433^,

J. Lizer^1434^,

Giambattista Lobreglio^1435^,

Cher Lowies^1436^,

Cheng Lu^1437^,

Haroon Majeed^1438^,

Adam Martin^1439^,

Luis Martinez-Sobrido^1440^,

Edwin Meresh^1441^,

Marianne Middelveen^1442^,

Alireza Mohebbi^1443^,

Jorge Mota^1444^,

Zahra Mozaheb^1445^,

Ley Muyaya^1446^,

Amar Nandhakumar^1447^,

Sheryl H. X. Ng^1448^,

Monther Obeidat^1449^,

Deog-Hwan Oh^1450^,

Mohammed Owais^1451^,

Pia Pace-Asciak^1452^,

Ajay Panwar^1453^,

Caroline Park^341^,

Chris Patterson^1013^,

Felipe Penagos-Tabaree^1454^,

Paolo T. Pianosi^1455^,

Valentina Pinzi^1456^,

Clare Pridans^778^,

Anna Psaroulaki^1458^,

Ravi Kumar Pujala^1459^,

Leonardo Pulido-Arjona^1460^,

Peng-Fei Qi^591^,

Proton Rahman^1461^,

Nayanjot K. Rai^1462^,

Tienush Rassaf^1463^,

Julie Refardt^1464^,

Walter Ricciardi^1217^,

Olaf Riess^1465^,

Alexandros Rovas^1466^,

Frank M. Sacks^1467^,

Sherif Saleh^1468^,

Christopher Sampson^1469^,

Axel Schmutz^1470^,

Robert Sepanski^1471^,

Neeraj Sharma^1472^,

Manisha Singh^1473^,

Paul Spearman^1474^,

Mehala Subramaniapillai^1475^,

Ritu Swali^1476^,

Cher M. Tan^1477^,

Juan I. Tellechea^1478^,

Lisa-Marie Thomas^1479^,

Xin Tong^1408^,

Demetrios G. Vavvas^1480^,

Ralf Veys^1481^,

Veronica Vitriol^1482^,

Horng-Dar Wang^1483^,

Jinhui Wang^1484^,

Jiucun Wang^1239^,

Jason Waugh^1485^,

S. A. Webb^1486^,

Brendan A. Williams^1487^,

Alan D. Workman^1488^,

Tingxiu Xiang^1489^,

Li-Xin Xie^1490^,

Jun Xu^1491^,

Taosheng Xu^1492^,

Chongjun Yang^1493^,

Jihoon G. Yoon^1494^,

Christina M. Yuan^1495^,

Arno Zaritsky^1496^,

Yao Zhang^1497^,

Haochen Zhao^1498^,

Hannah Zuckerman^1499^,

Ran Lyu^1^,

Wayne Pullan^1^

3. Department of Medicine / Medical Oncology, University of Colorado Anschutz Medical Campus, CO, USA;

4. Botany Department, Faculty of Science, Tanta University, Tanta, Egypt;

5. Institute of Human Genetics, Jena University Hospital, Friedrich Schiller University, Jena, Germany;

6. Department of Radiation Oncology, University Medical Center Schleswig-Holstein, Campus Kiel, Germany;

7. Agricultural Research Service, U.S. Department of Agriculture, Plum Island Animal Disease Center, Greenport, NY 11944, USA;

8. Department of Biological and Environmental Science, University of Jyvaskyla, Finland;

9. Institute of Environmental Biotechnology, Graz University of Technology, Graz, Austria;

10. Department of Macromolecular Structures, National Center for Biotechnology (CNB-CSIC), Madrid, Spain;

11.Oral and Maxillofacial Radiology, Applied Oral Sciences and Community Dental Care, Faculty of Dentistry, The University of Hong Kong, Hong Kong, China;

12. Biodiversity Institute, Division of Entomology, University of Kansas, KS, USA;

13. The RNA Institute, University at Albany, State University of New York, Albany, NY, USA;

14. Department for Methods Development and Research Infrastructure, Robert Koch Institute, Berlin, Germany;

15. Department of Surgical and Conservative Pediatrics and Adolescent Medicine, Martin Luther University of Halle-Wittenberg, Halle, Germany;

16. Department of BioHealth Informatics, IU School of Informatics and Computing, Indiana University-Purdue University, IN, USA;

17. School of Pharmacy, Stem Cell Biology and Regenerative Medicine, University of Reading, UK;

18. School of Engineering, Dali University, Dali City, Yunnan Province, China;

19. Department of Psychiatry and Psychotherapy, University Hospital Munich (LMU), Munich, Germany;

20. Faculty of Life and Environmental Sciences, University of Tsukuba, Ibaraki, Japan;

21. Aghyle, UniLaSalle, Beauvais, France;

22. Department of Immunology, Henry Ford Health System, Michigan, US;

23. Department of Emergency Medicine, Sir Run Run Shaw Hospital, Zhejiang University School of Medicine, Hangzhou 310016, China;

24. Anesthesia and Pain Medicine, Istituto Nazionale Tumori. Fondazione Pascale. IRCCS, Napoli, Italy;

25. Department of Transfusion Medicine, National Institutes of Health, Bethesda, MD, USA;

26. Institute of Infection, Immunity & Inflammation, University of Glasgow, UK;

27. Institute of Human Genetics, Polish Academy of Sciences, Poznan, Poland;

28. Department of Surgery, Oncology and Gastroenterology (DISCOG), University of Padua, Padova, Italy;

29. Biomedical Engineering, Eindhoven University of Technology, Eindhoven, Netherlands;

30. Institut de Biologie Moléculaire et Cellulaire (IBMC), University of Strasbourg, France;

31. Department of General, Visceral and Transplantation Surgery, University of Heidelberg, Germany;

32. Psychology, University of Notre Dame, Notre Dame, IN, USA;

33. Radiology and Pathology, Massachusetts General Hospital, Harvard Medical School, Boston, MA, USA;

34. Department of Biological Sciences, Auburn University, AL, USA;

35. KK Research Centre, KK Women’s And Children’s Hospital, Singapore;

36. Educational Psychology, University of Alabama, Tuscaloosa, AL, USA;

37. Wellcome/EPSRC Centre for Interventional & Surgical Sciences, University College London, UK;

38. Department of Nutrition and Movement Sciences, Maastricht University, Maastricht, Netherlands;

39. Department of Radiation Oncology, University of Arkansas for Medical Sciences, Little Rock, AR, USA;

40. Department of Land, Environment, Agriculture and Forestry, University of Padua, Padova, Italy;

41. Department of Biological and Environmental Sciences, University of Gothenburg, Sweden;

42. Priority Research Centre for Reproductive Science, The University of Newcastle, Callaghan, NSW, Australia;

43. Medical School, University of Cyprus, Nicosia, Cyprus;

44. Agricultural Big-Data Research Center, College of Plant Protection, Shandong Agricultural University, Taian, Shandong, China;

45. Laboratory of Neuropsychology, National Institute of Mental Health, Bethesda, MD, USA;

46. Wisconsin Institute for Discovery, University of Wisconsin, Madison, WI, USA;

47. Structural Genomics Consortium, University of Oxford, UK;

48. Bioinformatics, TUM Campus Straubing for Biotechnology and Sustainability, Weihenstephan-Triesdorf University of Applied Sciences, Straubing, Germany;

49. Cardiovascular Research, University of Michigan, MI, USA;

50. Pharmaceutical Chemistry, Bombay College of Pharmacy, Mumbai, India;

51. Developmental Neurobiology, Instituto de Investigaciones Biologicas Clemente Estable, Uruguay;

52. Surgical Gastroenterology and HPB Surgery, Gem Hospital & Research Centre, Coimbatore, India;

53. Department of Zoology, Faculty of Science, Kafr El Sheikh University, Kafr El Sheikh, Egypt;

54. Deparment of Internal Medicine IV, Medical University of Innsbruck, Austria;

55. Division of Biomedical Science and Biochemistry, The Australian National University, Canberra, ACT, Australia;

56. Applied Technology for Neuro-Psychology Lab., Catholic University of the Sacred Heart, Milan, Italy;

57. Medical Entomology, Shandong Institute of Parasitic Diseases, Shandong, China;

58. School of Pharmacy, Kumamoto University, Kumamoto, Japan;

59. Department of Biological Chemistry, Graduate School of Pharmaceutical Sciences, Nagoya City University, Nagoya, Japan;

60. Jamil-ur-Rahman Center for Genome Research, PCMD, ICCBS, University of Karachi, 75270-Karachi, Pakistan;

61. labormedizinisches zentrum Dr Risch, Vaduz, Liechtenstein;

62. Medicine, University of Otago, Dunedin, New Zealand;

63. Maternal Fetal Medicine, KK Women’s And Children’s Hospital, Singapore;

64. G.E.R.N. Tissue Replacement, Regeneration & Neogenesis, Department of Orthopedics and Trauma Surgery, Medical Center - Albert-Ludwigs-University of Freiburg, Faculty of Medicine, Albert-Ludwigs-University of Freiburg, Germany;

65. Institute of Biochemistry & Molecular Biology, Heinrich Heine University, Duesseldorf, Germany;

66. Surgery, National University of Ireland Galway, Ireland;

67. Institute of Agriculture, The University of Western Australia, Perth, WA, Australia;

68. Nuffield Department of Medicine, University of Oxford, UK;

69. Punggol Polyclinic, SingHealth Polyclinics, Singapore;

70. Division of Biological Chemistry, Biocenter, Medical University of Innsbruck, Austria;

71. Biochemistry and Genetics, La Trobe Institute for Molecular Sciences, La Trobe University, Melbourne, VIC, Australia;

72. INRA, University of Bordeaux, France;

73. Agriculture and Agri-Food Canada, Lethbridge Research and Development Centre, Lethbridge, Canada;

74. Trauma and Orthopaedics, St George’s Hospital, London, UK;

75. Department of Protein Evolution, Max Planck Institute for Developmental Biology, Tübingen, Germany;

76. 2nd Department of Gynaecology, Medical University of Lublin, Poland;

77. ICSI Analytics, National Research and Development Institute for Cryogenics and Isotopic Technologies – I.C.I.T. Ramnicu Valcea, VL, Romania;

78. Occupational Therapy, University of KwaZulu-Natal, Westville Campus, Durban, South Africa;

79. Joint Department of Physics, The Institute of Cancer Research, London, UK;

80. Department of Molecular Medicine and Surgery, Karolinska Institute, Solna, Sweden;

81. Orthopaedics, The Second Affiliated Hospital of Wenzhou Medical University, Wenzhou, Zhejiang, China;

82. Laboratorio de Virología Molecular, Centro de Investigaciones Nucleares, Facultad de Ciencias, Universidad de la República, Uruguay;

83. Department of Medical Biochemistry & Cell Biology, University of Gothenburg, Sweden;

84. Pediatric Cardiology, Charité Medical University of Berlin, Germany;

85. Neurorehabilitation Unit, Department of Neurosciences and Neurorehabilitation, Bambino Gesù Children's Hospital, Rome, Italy;

86. Microbiology and Immunology, Indiana University School of Medicine-Northwest, Gary, IN, USA;

87. Department of Behavioural and Molecular Neurobiology, University of Regensburg, Germany;

88. The University of Queensland Diamantina Institute, The University of Queensland & Translational Research Institute, Brisbane, QLD, Australia;

89. School of Biomedical Sciences, The University of Hong Kong, Hong Kong, China;

90. Pharmaceutical Sciences Department, St. Jude Children’s Research Hospital, Memphis, TN, USA;

91. Music, Durham University, Durham, UK;

92. Department of Hygiene and Epidemiology, University of Ioannina Medical School, Ioannina, Greece;

93. School of Life Sciences Weihenstephan, Technical University München, Maximus-von-Imhof-Forum 3, D-85354, Freising, Germany;

94. Institute for Transfusion Medicine, University Hospital Essen, Germany;

95. Computer Science, Virginia Commonwealth University, Richmond, VA, USA;

96. Institute for Social Science Research, The University of Queensland, Brisbane, QLD, Australia;

97. Centre International de Recherche en Infectiologie, Université de Lyon, France;

98. School of Allied Health Sciences, Griffith University, Gold Coast, QLD, Australia;

99. Department of Biochemistry and Molecular Biology, University of Melbourne, Parkville, VIC, Australia;

100. Department of Biohealth Informatics, School of Informatics and Computing, Indiana University-Purdue University, IN, USA;

101. Institute for Lung Research, Philipps-University Marburg, Germany;

102. Department of Biology, Indiana University, Bloomington, IN, USA;

103. Oral Biology, University of Florida, Gainesville, FL, USA;

104. Department of Biochemistry, University of Colorado, Boulder, CO, USA;

105. Center for the Promotion of Interdisciplinary Education and Research, Kyoto University, Kyoto, Japan;

106. Institute for Virus Diagnostics, Friedrich Loeffler Institute, Insel Riems, Greifswald, Germany;

107. Department of Zoology, University of Oxford, UK;

108. Department of Bioinformatics, Technical University of Munich, München, Germany;

109. School of Animal and Veterinary Sciences, University of Adelaide, SA, Australia;

110. Cancer Biology, Stanford University, Palo Alto, CA, USA;

111. Department of Electrical and Information Engineering, Covenant University, Ota, Nigeria;

112. Institute for Stem Cell Research and Regenerative Medicine, Heinrich Heine University, Düsseldorf, Germany;

113. Faculty of Pharmaceutical Sciences, The University of British Columbia, Vancouver, BC, Canada;

114. Psychiatry, Albert Einstein College of Medicine, New York City, NY, USA;

115. Medical Biology and Genetics, Akdeniz University, Antalya, Turkey;

116. National Institute of Mental Health, National Institutes of Health, Bethesda, MD, USA;

117. Internal Medicine I, Medical University Hospital, Tübingen, Germany;

118. Netelabs, NET ESolutions, McLean, VA, USA;

119. Institute of Public Health, University of Southern Denmark, Odense, Denmark;

120. Molecular Microbiology, John Innes Centre, Norwich, UK;

121. Australian Research Council Centre of Excellence in Plant Energy Biology, Waite Research Precinct, University of Adelaide, SA, Australia;

122. Cambridge Institute of Public Health, University of Cambridge, UK;

123. Institute for Biochemistry, University of Muenster, Germany;

124. National Centre for Epidemiology and Population Health (RSPH), The Australian National University, Canberra, ACT, Australia;

125. Neurobiology and Bioengineering, Stanford University, Palo Alto, CA, USA;

126. Geography, Earth and Environmental Sciences, University of Birmingham, UK;

127. School of Veterinary & Life Sciences, Murdoch University, Perth, WA, Australia;

128. Center for Climate Change Communication, George Mason University, Fairfax, VA, USA;

129. School of Agriculture, Food and Wine, University of Adelaide, SA, Australia;

130. School of Pharmaceutical Sciences, Southern Medical University, Guangzhou, China;

131. Pathology, Stanford University, Palo Alto, CA, USA;

132. Department of Experimental, Diagnostic and Specialty Medicine, University of Bologna, Italy;

133. Department of General Psychology, University of Padua, Padova, Italy;

134. Department of Bioinformatics & Systems Biology, Key Laboratory of Molecular Biophysics of Ministry of Education, College of Life Science and Technology, and the Collaborative Innovation Center for Biomedical Engineering, Huazhong University of Science and Technology, Wuhan, Hubei, China;

135. School of Biomedical Sciences, Queensland University of Technology, Brisbane, QLD, Australia;

136. Institut de Biologie Physico-Chimique, French National Center for Scientific Research (CNRS), Paris, France;

137. Department of Sociology, University of Warwick, Coventry, UK;

138. Structural and Computational Biology, European Molecular Biology Laboratory, Heidelberg, Germany;

139. Cancer Biology, University of Cincinnati College of Medicine, Cincinnati, OH, USA;

140. Biology, University of Waterloo, ON, Canada;

141. Sorbonne Université, CNRS, Integrative Biology of Marine Models (LBI2M), Station Biologique de Roscoff (SBR), Roscoff, France;

142. Biochemistry and Medical Genetics, University of Manitoba, Winnipeg, MB, Canada;

143. Ultrafast Solid State Spectroscopy, Max Planck Institute for Solid State Research, Stuttgart, Germany;

144. Department of Infectious Disease Epidemiology, School of Public Health, Imperial College London, UK;

145. Biochemistry, Faculty of Veterinary Medicine, Mansoura University, Mansoura, Egypt;

146. Molecular Physiology and Biological Physics, University of Virginia, Charlottesville, VA, USA;

147. State Key Laboratory of Biogeology and Environmental Geology, China University of Geosciences, Wuhan, China;

148. Child Psychiatry, Clinic for Neurology and Psychiatry for Children and Youth, Belgrade, Serbia, Belgrade;

149. Medical Physics, Ahvaz Jundishapur University of Medical Sciences, Ahvaz, Iran;

150. Division of Biostatistics and Computational Biology of College of Dentistry, Departments of Preventive & Community Dentistry, Biomedical Engineering, and Biostatistics, University of Iowa City, IA, USA;

151. Institute of Medical Psychology and Behavioural Neurobiology, University Tübingen, Germany;

152. Department of Zoology, University of Ibadan, Nigeria;

153. College of Veterinary Medicine, Kansas State University, Manhattan, KS, USA;

154. Discipline of ICT, School of Technology, Environments and Design, University of Tasmania, Hobart, Australia;

155. Radiology, Children’s Mercy Hospital, Kansas City, MO, USA;

156. Department of Physics, Stockholm University, Stockholm, Sweden;

157. Institute for Cancer Genetics and Informatics, Oslo University Hospital, Oslo, Norway;

158. CSIRO Manufacturing, CSIRO, Pullenvale, QLD, Australia;

159. Urology, Johns Hopkins School of Medicine, Baltimore, MD, USA;

160. Life and Consumer Sciences, University of South Africa, Johannesburg, South Africa;

161. Mechanisms of Induced Plasticity of the Brain, German Center for Neurodegenerative Diseases, Bonn, Germany;

162. Developmental Biology and Cancer, Institute of Child Health, University College London, UK;

163. Biomedical Physiology and Kinesiology, Simon Fraser University, Burnaby, BC, Canada;

164. Centre for Health Informatics, University of Manchester, UK;

165. School of Human Movement and Nutrition Sciences, The University of Queensland, Brisbane, QLD, Australia;

166. Department of Medicine, Albert Einstein College of Medicine, New York City, NY, USA;

167. Kennedy Institute of Ethics, Georgetown University, Washington, DC, USA;

168. Dermatology, Skin and Cancer Foundation, Carlton, VIC, Australia;

169. Divisions of Hematology/Oncology & Infectious Disease, Medical College of Wisconsin, Milwaukee, WI, USA;

170. Office of Biotechnology Products, U.S. Food and Drug Administration, Washington, DC, USA;

171. Biomedical Engineering, Worcester Polytechnic Institute, Worcester, MA, USA;

172. Centre for Orthopaedic and Trauma Research, University of Adelaide, SA, Australia;

173. National Infection Service, Public Health England, Bristol, UK;

174. Faculty of Science - Chemistry, Utrecht University, Netherlands;

175. Pathology, National University of Ireland Galway, Ireland;

176. National Heart and Lung Institute (NHLI), Imperial College London, UK;

177. Neurodegeneration, Danube Private University, Krems/Donau, Austria;

178. Department of Chemistry, Imperial College London, UK;

179. Finnish Museum of Natural History, University of Helsinki, Finland;

180. School of Life and Environmental Sciences, University of Sydney, NSW, Australia;

181. Nottingham Digestive Diseases Centre, University of Nottingham, UK;

182. ITAV USR3505 CNRS, University of Toulouse, France;

183. University of Queensland Diamantina Institute, The University of Queensland, Brisbane, QLD, Australia;

184. CZ-Openscreen, Institute of Molecular Genetics of the ASCR, Prague, Czech Republic;

185. Institute of Biomedicine, University of Turku, Finland;

186. School of Kinesiology and Health Science, York University, Toronto, ON, Canada;

187. Ophthalmology, Stein Eye Institute, University of California Los Angeles, CA, USA;

188. Agriculture and Agri-Food Canada, Ottawa Research and Development Center, Ottawa, ON, Canada;

189. Material Design and Characterization, Helmholtz-Zentrum Geesthacht, Germany;

190. Internal Medicine, Unversity of Genova, Italy;

191. Intelligent Catheter – INKS, Otto-von-Guericke-University, Magdeburg, Germany;

192. Cancer Biology, University of Toledo Health Science Campus, OH, USA;

193. Structural Biology, CRUK Beatson Institute, Glasgow, UK;

194. Medical RNA Biology, Leibniz Institute for Primate Research, Göttingen, Germany;

195. PEIRENE EA 7500, University of Limoges, France;

196. Veterinary Medicine, Hainan University, Haikou, Hainan, China;

197. Pharmacognosy and Phytotherapy, School of Pharmacy, University College London, UK;

198. Ecology and Genetics Research Unit, University of Oulu, Finland;

199. Institute of Biochemistry and Molecular Immunology, RWTH Aachen University, Aachen, Germany;

200. Institute of Biochemistry and Technical Biochemistry, University Stuttgart, Germany;

201. School of Chemical Biology and Biotechnology, Peking University Shenzhen Graduate School, Shenzhen, China;

202. Medical Oncology, GIGA-Research Institute & CHULiège, Liège, Belgium;

203. MPBA, Fondazione Bruno Kessler, Trento, Italy;

204. Department of Neurobiology, Hokkaido University Graduate School of Medicine, Sapporo, Japan;

205. Oncology, University of Leuven, Belgium;

206. Department of Mathematical Sciences, Chalmers University of Technology, Göteborg, Sweden;

207. Department of Neurology, Radboud University Medical Center, Nijmegen, Netherlands;

208. School of Information Technology and Mathematical Sciences, University of South Australia, Adelaide, SA, Australia;

209. Department of Computational Biology, Bio-Thera Solutions Ltd., Guangzhou, China;

210. Otolaryngology, Instituto de Investigación Biosanitario de Granada IBS.Granada, Spain;

211. School of Molecular and Life Sciences, Curtin University, Perth, WA, Australia;

212. Institute of Cellular Medicine, Newcastle University, Newcastle upon Tyne, UK;

213. Institute of Pharm. Biology/DCAL, Goethe University, Frankfurt, Germany;

214. UMR 5246 Institut de Chimie et Biochimie Moléculaires et Supramoléculaires (ICBMS), Université de Lyon, France;

215. Department of Genomes and Genetics, Institut Pasteur, Paris, France;

216. Pharmacology, University of Valencia, Spain;

217. Research Centre for Olive, Citrus and Tree Fruit, Council for Agricultural Research and Economics, Caserta, Italy;

218. Preclinical Pharmacology and In Vitro Toxicology, Fraunhofer Institute for Toxicology and Experimental Medicine (ITEM), Hannover, Germany;

219. School of Medicine, University of Tasmania, Hobart, TAS, Australia;

220. Oncology Lifecycle Management Department, Chugai Pharmaceutical Co. Ltd, Tokyo, Japan;

221. Institute of Neurobiology, University of Lübeck, Germany;

222. Neurology, New York University School of Medicine, New York, NY, USA;

223. Department of Plant Pathology, University Putra Malaysia, Seri Kembangan, Malaysia;

224. Biology, University of North Carolina at Chapel Hill, NC, USA;

225. Faculty of Health Sciences, University of Southampton, UK;

226. Genetics and Immunology Research Group, University of the Highlands and Islands, Inverness, UK;

227. Institute of Pathology, University of Heidelberg, Germany;

228. Department of Computational Biology, Indraprastha Institute of Information Technology, New Delhi, India;

229. School of Health and Life Sciences, Glasgow Caledonian University, Glasgow, UK;

230. Pharmacy and Biomolecular Sciences, Liverpool John Moores University, Liverpool, UK;

231. Basic Research, Parkinson’s Institute and Clinical Center, Sunnyvale, CA, USA;

232. Environmental Research and Innovation, Luxembourg Institute of Science and Technology, Luxembourg;

233. School of Computing and Information Technology, University of Wollongong, Wollongong, NSW, Australia;

234. Department of Biomedical Sciences and Human Oncology, Università degli Studi di Bari Aldo Moro, Bari-Italy;

235. College of Nursing and Health Sciences, Flinders University, Adelaide, SA, Australia;

236. Molecular Medicine, University of South Florida, Tampa, FL, USA;

237. Behavioral & Brain Sciences, The University of Texas at Dallas, Richardson, TX, USA;

238. Zoology & Entomology, Rhodes University, Grahamstown, South Africa;

239. Instituto de Higiene e Medicina Tropical, Universidade NOVA de Lisboa, Portugal;

240. Institute of Microbiology, ETH Zurich, Switzerland;

241. Immunohaematology, German Red Cross Blood Service, Institute Ulm, Germany;

242. Institute of Advanced Biomedical Engineering and Science, Tokyo Women’s Medical University, Tokyo, Japan;

243. Biological Sciences, University of Limerick, Ireland;

244. Department of Animal Science, McGill University, Montreal, QC, Canada;

245. StatSol, Lübeck, Germany and School of Mathematics, Statistics and Computer Science, University of KwaZulu-Natal, Pietermaritzburg, South Africa;

246. Institute of Systems Biology (INBIOSIS), Universiti Kebangsaan Malaysia (UKM);

247. National Center for Complementary and Integrative Health, National Institutes of Health, Bethesda, MD, USA;

248. Family Medicine Research Division, University of Kansas Medical Center, Kansas City, KS, USA;

249. Multimodal Molecular Biology, Institute of Molecular and Cell Biology, Agency for Science, Technology and Research (A*STAR), Singapore;

250. Department of Chronic Diseases, Metabolism and Ageing, University of Leuven, Belgium;

251. International Iberian Nanotechnology Laboratory (INL), Braga, Portugal;

252. Computing and Technology, Anglia Ruskin, Cambridge, UK;

253. Structural Cell Biology, Max Planck Institute of Biochemistry, Planegg, Germany;

254. Department of Computer Architecture and Technology, Universidad de Sevilla, Spain;

255. EBIO, University of Colorado, Boulder, CO, USA;

256. Cancer Sciences, University of Manchester, UK;

257. Research School of Population Health, The Australian National University, Canberra, ACT, Australia;

258. Bio-systems, Singapore-MIT Alliance for Research and Technology, Singapore;

259. Musculoskeletal Health and Ageing Research Program, Mary MacKillop Institute for Health Research, Australian Catholic University, Melbourne, VIC, Australia;

260. General Surgery, St. Josef Hospital - Ruhr University Bochum, Germany;

261. College of Life Sciences, Northwest A&F University, Shaanxi, China;

262. Division of Plant Science, Research School of Biology, The Australian National University, Canberra, ACT, Australia;

263. Clinical and Nonclinical Research, Battelle, Columbus, OH, USA;

264. Biological Sciences, Southern Methodist University, Dallas, TX, USA;

265. Institute of Medical Mycology, Teikyo University, Tokyo, Japan;

266. Bioinformatics, Tempus Labs, Chicago, IL, USA;

267. College of Biology, Hunan University, Changsha, China;

269. FutureNeuro Research Centre, Physiology & Medical Physics, Royal College of Surgeons in Ireland, Dublin, Ireland;

270. Gerontology Research Center, University of Jyvaskyla, Finland;

271. Department of Anesthesiology, University of Heidelberg, Germany;

272. Biology, Pennsylvania State University, PA, USA;

273. State Key Laboratory of Microbial Resources, Institute of Microbiology, Chinese Academy of Sciences, Beijing, China;

274. School of Life Sciences, University of Science and Technology of China, Anhui, China;

275. Animal Science, Michigan State University, East Lansing, MI, USA;

276. Buffalo Neuroimaging Analysis Center, Department of Neurology, Jacobs School of Medicine and Biomedical Sciences, University at Buffalo, State University of New York, NY, USA;

277. Institute for Biological Sciences, University of Rostock, Germany;

278. Environmental Research Institute, School of Environment, South China Normal University, Guangzhou, China;

279. Department of Veterinary Medicine, University of Bari, Italy;

280. Primary and Community Care, Radboud University Medical Center, Nijmegen, Netherlands;

281. EcoHealth Alliance, New York, NY, USA;

282. Cardiovascular Department, Mayo Clinic, Rochester, MN, USA;

283. Department of Epidemiology, School of Medicine in Katowice, Medical University of Silesia, Katowice, Poland;

284. Animal Science, University of Lleida, Spain;

285. Pharmaceutical Outcomes & Policy, University of Florida College of Pharmacy, Gainesville, FL, USA;

286. Earth and Biological Science Directorate, Pacific Northwest National Laboratory, Richland, WA, USA;

287. Centre for Discovery Brain Sciences, University of Edinburgh, UK;

288. Department of Biomedical Informatics, Harvard Medical School, Boston, MA, USA;

289. Radiology, University of Pennsylvania, Philadelphia, PA, USA;

290. Biomedical Sciences - Personalized Medicine, Asthma and Allergy unit, Humanitas University and Research Hospital, Rozzano, Italy;

291. Department of Quantum Science, The Australian National University, Canberra, ACT, Australia;

292. Ecological Genomics, University of Oldenburg, Germany;

293. Paediatric Dentistry & Orthodontics, International Medical University, Kuala Lumpur, Malaysia;

294. School of Nursing, University of Ottawa, ON, Canada;

295. Department of Education Support, Maastricht University, Maastricht, Netherlands;

296. Mathematics, University of Manchester, UK;

297. School of Population Health Sciences, Faculty of Life Sciences and Medicine, King’s College London, UK;

298. Institute of Health and Biomedical Innovation, Queensland University of Technology, Brisbane, QLD, Australia;

299. Epidemiology, Public Health School, Harbin Medical University, Heilongjiang, China;

300. Department of Chemistry, UiT - The Arctic University of Norway, Tromsø, Norway;

301. Biological Statistics and Computational Biology, Cornell University, Ithaca, NY, USA;

302. Shanghai Key Lab for Urban Ecological Process and Eco-restoration, School of Ecological and Environmental Sciences, East China Normal University, Shanghai, China;

303. Faculty of Biology, Johannes Gutenberg University, Mainz, Germany;

304. Food Science, University of Massachusetts Amherst, MA, USA;

305. Complex Adaptive Traits Research Group, Max Planck Institute for Terrestrial Microbiology, Marburg, Germany;

306. Centre for Exercise and Rehabilitation Science, NIHR Biomedical Research Centre - Respiratory, Leicester, UK;

307. Section of Endocrinology, Department of Internal Medicine, Yale University, New Haven, CT, USA;

308. Department of Biosciences, Sardar Patel University, Gujarat, India;

309. Department of Applied Physics, The University of Tokyo, Japan;

310. Cologne Excellence Cluster for Cellular Stress Responses in Aging-Associated Diseases (CECAD), in vivo Research Facility (ivRF), University of Cologne, Germany;

311. Department of Public Health Science, National Open University of Nigeria, Lagos, Nigeria;

312. Department of Radiology, University Hospital Regensburg, Germany;

313. Geography, National University of Singapore;

314. College of Medicine, Alfaisal University, Riyadh, Saudi Arabia;

315. Biomedical Genomics and Oncogenetics Research Laboratory, Faculty of Sciences and Techniques of Tangier, Abdelmalek Essaadi University, Morocco;

316. Exercise Biology Group, Faculty of Sport and Health Sciences, Technical University of Munich, Germany;

317. Departamento de Psicoloxía Social, Básica e Metodoloxía, Universidade de Santiago de Compostela, Galiza, Spain;

318. Signal Processing Laboratory, Griffith University, Brisbane, QLD, Australia;

319. Department of Neurology, Ghent University and Ghent University Hospital, Belgium;

320. Faculty of Veterinary Medicine, Ghent University, Merelbeke, Belgium;

321. Institute for Clinical and Translational Research, Albert Einstein College of Medicine, New York City, NY, USA;

322. Faculty of Medical Science, Anglia Ruskin University, Cambridge, UK;

323. College of Chemistry and Molecular Engineering, Peking University, Beijing, China;

324. Department of Dermatology and Allergy, Herlev and Gentofte Hospital, Hellerup, Denmark;

325. Medical Imaging and Nuclear Medicine, Lady Cilento Children’s Hospital, Brisbane, QLD, Australia;

326. Nursing and Reproductive Health, School of Medicine and Allied Health Sciences, University of The Gambia, Brikama, Gambia;

327. Woolcock Institute of Medical Research, University of Sydney, NSW, Australia;

328. Computer Science, Wayne State University, Detroit, MI, USA;

329. Otolaryngology, Aberdeen Royal Infirmary, Aberdeen, Scotland;

330. Laboratory Medicine and Pathobiology, University of Toronto, ON, Canada;

331. Institute of Ageing, The Chinese University of Hong Kong, Hong Kong, China;

332. Emergency Medicine, University of Nebraska Medical Center, Omaha, NE, USA;

333. Pathology, University of Florida College of Medicine, Gainesville, FL, USA;

334. Radiation Oncology, MD Anderson Cancer Center, Houston, TX, USA;

335. Translational Research NTMS, University of Pisa, Italy;

336. Clinical and Experimental Medicine, Magna Grecia University, Catanzaro, Italy;

337. Pediatrics, Larner College of Medicine University of Vermont, Burlington, VT, USA;

338. Diagnostic and Interventional Ultrasound, Sun Yat-sen University Cancer Center, Guangzhou, China;

339. Institutes of Brain Science, Fudan University, Shanghai, China;

340. College of Agronomy, Shanxi Agricultural University, Shanxi, China;

341. Institute of Medical Science, University of Toronto, ON, Canada;

342. ARCPOH, University of Adelaide, SA, Australia;

343. Cardiology and Vascular Medicine, Faculty of Medicine, Pelita Harapan University, Tangerang, Indonesia;

344. Research Division, Institute of Mental Health, Singapore;

345. MRC Clinical Trials Unit, University College London, UK;

346. Department of Philosophy, Sociology, Education and Applied Psychology (FISPPA), University of Padua, Padova, Italy;

347. Department of Psychiatry and Psychotherapy, University of Lübeck, Lübeck, Germany;

348. Center for Molecular Medicine, Dalian University of Technology, Liaoning, China;

349. Reproductive Medicine, Tianjin United Family Healthcare, Tianjin, China;

350. Departament d’Enginyeria Química Biològica i Ambiental, Universitat Autònoma de Barcelona, Spain;

351. Department of Animal Science, Sao Paulo State University (UNESP), Sao Paulo, Brazil;

353. Department of Cardiovascular Medicine, The University of Tokyo, Japan;

354. Veterinary Clinical, Sao Paulo State University, Paulo, Brazil;

355. Institute of Biotechnology, Zhejiang University, Hangzhou, Zhejiang, China;

356. Department of Molecular Biology and Genetics, Aarhus University, Denmark;

357. Maternal and Fetal Health, St. Mary’s Hospital, University of Manchester, UK;

358. Internal Medicine, University of New Mexico, Albuquerque, NM, USA;

359. Division of Biomedical Statistics and Informatics, Mayo Clinic, Jacksonville, FL, USA;

360. Plant Production, King Saud University, Riyadh, Saudi Arabia;

361. Department of Molecular Biology, Institute of Genetics and Animal Breeding of the Polish Academy of Sciences;

362. Optometry and Vision Science, Queensland University of Technology, Brisbane, QLD, Australia;

363. School of Life Sciences, University of Nottingham, UK;

364. Cancer Research Centre, Cancer Council Queensland, Brisbane, QLD, Australia;

365. Department of Chemistry, Umeå University, Umeå Sweden;

366. Centre for Radiation, Chemical & Environmental Hazards, Public Health England, Bristol, UK;

367. DISTABIF, Università della Campania ”L. Vanvitelli”, Caserta, Italy;

368. Obstetrics and Gyanecology, Bartshealth, London, UK;

369. GReD Laboratory, Université Clermont Auvergne, Clermont-Ferrand, France;

370. INRA, Abeilles et Environnement, Avignon, France;

371. School of Public Health and Social Work, Queensland University of Technology, Brisbane, QLD, Australia;

372. Psychiatry, University of Pennsylvania, Philadelphia, PA, USA;

373. Research Institute of Physiology and Basic Medicine, Novosibirsk State University, Russia;

374. Department of Chemistry, University College London, UK;

375. Animal, Plant and Soil Sciences, La Trobe University, Melbourne, VIC, Australia;

376. Epidemiology and Biostatistics, University of Arizona, Tucson, AZ, USA;

377. ERIBA, University Medical Center Groningen, University of Groningen, Netherlands;

378. Department of Radiation Physics, Institution of Clinical Science, University of Gothenburg, Sweden;

379. Biochemistry and Molecular Biology, SUNY Upstate Medical University, Syracuse, NY, USA;

380. Centro de Pesquisas Goncalo Moniz, Fundacao Oswaldo Cruz, Bahia, Brazil;

381. Food, Water and Environmental Microbiology, Institute of Environmental Science & Research (ESR), Christchurch, New Zealand;

382. Food Science, University of Otago, Dunedin, New Zealand;

383. Biomedical Science, Florida Atlantic University, Boca Raton, FL, USA;

384. Institute for Molecular Bioscience, The University of Queensland, Brisbane, QLD, Australia;

385. Institute of Clinical Neurobiology, University Hospital Würzburg, Germany;

386. Public Health, Institute of Tropical Medicine, Antwerpen, Belgium;

387. Department of Rheumatology and Clinical Immunology, Faculty of Medicine, School of Health Sciences, University of Thessaly, Larissa, Greece;

388. Department of Orthodontics and Pediatric Dentistry, UZB University Center for Dental Medicine, University of Basel, Switzerland;

389. Institut Systématique, Evolution, Biodiversité (ISYEB), UMR 7205 MNHN-CNRS-Sorbonne Université-EPHE, Museum National d’Histoire Naturelle, Paris, France;

390. Institute for Advanced Bioscience, CNRS, France;

391. School of Molecular Sciences, The University of Western Australia, Perth, WA, Australia;

392. Biochemistry and Microbiology, University of Fort Hare, South Africa;

393. Physics, University Tübingen, Germany;

394. School of Environment and Science, Griffith University, Brisbane, QLD, Australia;

395. Statistics and Standardised Methods, Philosophisch-Theologische Hochschule Vallendar, Germany;

396. Life Sciences, Imperial College London, UK;

397. Adelaide Medical School, University of Adelaide, SA, Australia;

398. Department of Pharmacology and Experimental Neuroscience, University of Nebraska Medical Center, Omaha, NE, USA;

399. FOCAS Research Institute, Technological University Dublin, Ireland;

400. Cancer Research Center of Toulouse, National Institute for Health and Medical Research (INSERM), Paris, France;

401. Grupo BRAINSHARK, Departamento de Bioloxía Funcional, Universidade de Santiago de Compostela, Galiza, Spain;

402. Biology, University of Nebraska at Kearney, NE, USA;

403. Departament de Genètica i Microbiologia, Universitat Autònoma de Barcelona, Spain;

404. Chemistry and Industrial Chemistry, University of Pisa, Italy;

405. Wellcome Trust Centre for Cell-Matrix Research, University of Manchester, UK;

406. Institute of Human Genetics, CNRS, University of Montpellier, France;

407. Institute of Organic Chemistry and Biochemistry, Czech Academy of Sciences, Prague, Czech Republic;

408. Computational Systems Biology Group, National Center for Biotechnology (CNB-CSIC), Madrid, Spain;

409. Department of Biochemistry and Molecular Biology, National Cheng Kung University, Tainan, Taiwan;

410. Ophthalmology, Schepens Eye Research Institute, Harvard Medical School, Boston, MA, USA;

411. Department of General Surgery, Lanzhou University Second Hospital, Lanzhou, China;

412. School of Life Sciences, University of Technology Sydney, NSW, Australia;

413. Gynecology, JT Chen Clinic, Tokyo, Japan;

414. Institute of Agriculture, and School of Agriculture and Environment, The University of Western Australia, Perth, WA, Australia;

415. Faculty of Geographical Science, Beijing Normal University, Beijing, China;

416. Electrical Engineering and Computer Science, University of Missouri, Columbia, MO, USA;

417. Department of Public and Occupational Health, Amsterdam Public Health Research Institute, VU University Medical Center, Amsterdam, Netherlands;

418. Medical Biochemistry, Semmelweis University, Budapest, Hungary;

419. Department of Clinical Oncology, Queen Elizabeth Hospital, Hong Kong, China;

420. Chemistry, University of Pittsburgh, PA, USA;

421. Neurology, G B Pant Institute of Post Graduate Medical Education and Research, New Delhi, India;

422. Veterinary and Animal Sciences, University of Copenhagen, Denmark;

423. Section of Endocrinology, Biomedical Department of Internal and Specialist Medicine (DIBIMIS), University of Palermo, Italy;

424. Neuroscience, Physiology, and Pharmacology (NPP), University College London, UK;

425. Microbiology and Cell Science, University of Florida, Gainesville, FL, USA;

426. School of Health Sciences, University of Salford, Manchester, UK;

427. Institute of Medical Genetics, Cardiff University, UK;

428. ICO Cancer Center, Inserm, Angers, France;

429. Microbiology, Faculty of Medicine, University of Colombo, Sri Lanka;

431. Faculty of Pharmacy, University of Porto, Portugal;

432. Division of Primary Care, University of Nottingham, UK;

433. Pharmacy Systems, Outcomes and Policy, University of Illinois at Chicago, IL, USA;

434. Escola de Ciências Agrárias e Biológicas, Pontifícia Universidade Católica de Goiás, Brazil;

435. Department of Oncology, China-Japan Friendship Hospital, Beijing, China;

436. Chemistry, Boston University, MA, USA;

437. Environmental Science, American University, Washington, DC, USA;

438. Neuroscience, Carleton University, Ottawa, ON, Canada;

439. Chemistry and Biochemistry, University of Regina, SK, Canada;

440. Microbiolgy, Universite de Montreal, Montreal, QC, Canada;

441. Department of Genetics and Genome Biology, University of Leicester, UK;

442. School of Agriculture and Food Sciences, The University of Queensland, Gatton, QLD, Australia;

443. BIOM, CNRS, France;

444. The Hatter Cardiovascular Institute, University College London, UK;

445. Science and Engineering, Flinders University, Adelaide, SA, Australia;

446. Engineering, University of Warwick, Coventry, UK;

447. Center for Human Genetics, KU Leuven, Belgium;

448. Department of Macromolecular Science, Fudan University, Shanghai, China;

449. Biology Education, Dokuz Eylul University, Izmir, Turkey;

450. Department of Biochemistry and Molecular Biology, Indiana University School of Medicine, Indianapolis, IN, USA;

451. Pulmonary Disease, Mondo Medico, Borgomanero, Italy;

452. U.S. Naval Research Laboratory, Center for Bio/Molecular Science & Engineering, Washington, DC, USA;

453. Department of Nutrition, Institute of Basic Medical Sciences, University of Oslo, Norway;

454. Department of Mechanics and Engineering Science, Peking University, Beijing, China;

455. Department of Pharmacy, Pharmaceutical and Medicinal Chemistry, Saarland University, Saarbrücken, Germany;

456. Skin Research Institute, National Institute for Health and Medical Research (INSERM), Paris, France;

457. Research & Development, Shanghai Proton and Heavy Ion Center, Shanghai, China;

458. Epidemiology, University of Georgia, Athens, GA, USA;

459. Department of Plastic and Hand Surgery, Medical Center, University of Freiburg, Germany;

460. Research, Sidra Medicine, Doha, Qatar;

461. Department of Oral, Maxillofacial and Plastic Surgery, Rostock University Medical Center, Germany;

462. Emergency Medicine, Brigham & Women’s Hospital / Harvard Medical School, Boston, MA, USA;

463. Forage Science, AgResearch, Palmerston North, New Zealand;

464. Medicine, University of California Los Angeles, CA, USA;

465. Virginia G. Piper Center for Personalized Diagnostics, Biodesign Institute, Arizona State University, Tempe, AZ, USA;

466. Materials and Engineering Research Institute, Sheffield Hallam University, Sheffield, UK;

467. respiratory medicine, Aneurin Bevan University Healthboard, Newport, UK;

468. Neurological Surgery, University of California San Francisco, CA, USA;

469. UWA Dental School, The University of Western Australia, Perth, WA, Australia;

470. Biological Sciences, Fordham University, Bronx, NY, USA;

471. Institute of Biotechnology, University of Helsinki, Finland;

472. College of Life Sciences, Fujian Normal University, Fujian, China;

473. Department of Frontier Fiber Technology and Science, University of Fukui, Japan;

474. Clinical & Experimental Sciences, Faculty of Medicine, University of Southampton, UK;

475. Department of Biomolecular Sciences, University of Urbino, Italy;

476. Allergology Unit, Ospedale San Luigi, Torino, Italy;

477. Department of Urology, Muljibhai Patel Urological Hospital, Gujarat, India;

478. Stratigraphy and Paleontology, University of Granada, Spain;

479. School of Veterinary Sciences, Massey University, Auckland, New Zealand;

480. High Performance Computing and Networking Institute, National Research Council, Naples, Italy;

481. Division of Metabolism and Children’s Research Center, University Children’s Hospital Zürich, Switzerland;

482. Biochemistry and Molecular Biology, University of Melbourne, Parkville, VIC, Australia;

483. Leptospirosis Research and Expertise Unit, Institut Pasteur, Noumea, New Caledonia;

484. School of Life Sciences, Tsinghua University, Beijing, China;

485. Biology Animal Department, Sciences Faculty, Cheikh Anta Diop University (UCAD), Dakar, Senegal;

486. Earle A. Chiles Research Institute, Providence Portland Medical Center, Portland, OR, USA;

487. Agriculture and Agri-Food Canada, Lacombe Research and Development Centre, Lacombe, Canada;

488. Performance Management and Evaluation, Alberta Innovates, Edmonton, AB, Canada;

489. Instituto de Hortofruticultura Subtropical y Mediterránea ”La Mayora” (IHSM-UMA-CSIC), Universidad de Málaga-Consejo Superior de Investigaciones Científicas, Spain;

490. College of Public Health, Medical and Veterinary Sciences, James Cook University, Cairns, QLD, Australia;

491. Joint Research Centre, European Commission, Ispra, Italy;

492. University of Montpellier, France;

493. Commonwealth Scientific and Industrial Research Organisation (CSIRO), Floreat, WA, Australia;

494. Sun Yat-sen Memorial Hospital, Sun Yat-sen University, Guangzhou, Guangdong, China;

495. Pharmacology, Vanderbilt University, Nashville, TN, USA;

496. KFU-RIKEN Translational Genomics Unit, RIKEN, Yokohama, Japan;

497. Neurobiology of Vocal Communication, University Tübingen, Germany;

498. UCCS center for the Biofrontiers Institute, University of Colorado at Colorado Springs, CO, USA;

499. Division of Basic Sciences, Fred Hutchinson Cancer Center, Seattle, WA, USA;

500. Primary Care Unit, Faculty of Medicine, University of Geneva, Switzerland;

501. Department of Molecular Genetics and Infection Biology, Universität Greifswald, Germany;

502. Department of Fine Chemistry, East China University of Science and Technology, Shanghai, China;

503. Surgery, The Ohio State University, Columbus, OH, USA;

504. R&D, Oragenics, Tampa, FL, USA;

505. Environmental Health Institute, National Environment Agency, Singapore;

506. Institute of Diagnostic Virology, Friedrich Loeffler Institute, Insel Riems, Greifswald, Germany;

507. Oncology, The Queen Elizabeth Hosptal, Woodville, SA, Australia;

508. Emerging Pests and Pathogens Research Unit, USDA, NY, USA;

509. Epilepsy Center, Department of Neurosurgery, Medical Center, University of Freiburg, Germany;

510. Information and Technology Studies, Faculty of Education, The University of Hong Kong, Hong Kong, China;

511. Agricultural and Environmental Biology, Graduate School of Agricultural and life Sciences, The University of Tokyo, Japan;

512. Department of Medicine, Pittsburgh Heart, Lung, and Blood Vascular Medicine Institute, University of Pittsburgh, PA, USA;

513. Directorate of Transplant, Renal and Urology, Guy’s & St Thomas’ NHS Foundation Trust, London, UK;

514. Clinic for Heart, Blood Vessel and Rheumatic Diseases, Clinical Center, University of Sarajevo, Sarajevo;

515. School of Mathematics, Statistics and Actuarial Science, University of Kent, UK;

516. Department of Life Science and Technology, Tokyo Institute of Technology, Japan;

517. Laser Center Department of Applied Sciences and Mechatronics, University of Applied Sciences Munich, Germany;

518. Nanodevices, CIC nanoGUNE, San Sebastian, Spain;

519. Gynaecology, VU University Medical Center, Amsterdam, Netherlands;

520. Division of Population Medicine, Cardiff University Medical School, UK;

521. Department of Medicine, Karolinska Institute, Solna, Sweden;

522. Center for Information Biology, National Institute of Genetics, Mishima, Japan;

523. Harry Perkins Institute of Medical Research, Murdoch University, Perth, WA, Australia;

524. Physical Education & Sport Sciences, University of Limerick, Ireland;

525. Campus Clinic Gynecology, Ruhr-University Bochum Universitätsstr, Bochum, Germany;

526. Epidemiology and Biostatistics, School of Public Health, Southwest Medical University, Sichuan, China;

527. Center for Molecular Diagnostics, Key laboratory of Carcinogenesis and Translational Research (Ministry of Education/Beijing), Beijing Cancer Hospital, Beijing, China;

528. Herpetological Department, Chengdu Institute of Biology, Chinese Academy of Sciences, Chengdu, China;

529. Key Laboratory of Nano Biological Effects and Safety, Beijing, China;

530. PK Sciences, Novartis Institutes for Biomedical Research (NIBR), Basel, Switzerland;

531. Pain Center, Daejeon St. Mary’s Hospital, Daejeon, Korea;

532. School of Health Studies, The University of Western Ontario, London, ON, Canada;

533. Health Psychology Group, University of Aberdeen, UK;

534. Anesthesiology, University of Colorado Anschutz Medical Campus, CO, USA;

535. Critical Care Medicine, UZA (Antwerp University Hospital), University of Antwerp, Edegem, Belgium;

536. Endocrinology, Aarhus University Hospital, Denmark;

537. Industrial and Systems Engineering, Centre for Transport Development, University of Pretoria, South Africa;

538. Department of Biostatistics and Bioinformatics, Duke University, NC, USA;

539. Medical, Pharmaceutical and Biomedical Sciences School, Pontifícia Universidade Católica de Goiás, Brazil;

540. Division of Cardiovascular Medicine, Department of Clinical Sciences, Danderyd Hospital, Karolinska Institutet, Stockholm, Sweden;

541. Clinical, Educational and Health Psychology, University College London, UK;

542. Development and Differentiation Research Center, Korea Research Institute of Bioscience and Biotechnology (KRIBB);

543. Laboratoire de la Barrière Hémato-encéphalique, Université d’Artois, Arras, France;

544. Quantitative Biology, Discovery Sciences, IMED Biotech Unit, AstraZeneca, Cambridge, UK;

545. Environmental Molecular Sciences Laboratory, Pacific Northwest National Laboratory, Richland, WA, USA;

546. Statistical Physics Group, Applied Maths Research Centre, Coventry University, UK;

547. Invista Performance Technologies, The Wilton Centre, Cleveland, UK;

548. Genome Research, Thünen Institute of Forest Genetics, Großhansdorf, Germany;

549. Natural Sciences, Lebanese American University, Byblos, Lebanon;

550. Evidence and Value Generation, Takeda, Osaka, Japan;

551. Stem Cell and Regenerative Medicine, King Abdullah International Medical Research Center, Riyadh, Saudi Arabia;

552. Industries - Pulp and Paper, University of Bahri, Khartoum, Sudan;

553. Mater Research Institute-The University of Queensland, Mater Medical Research Institute, Brisbane, QLD, Australia;

554. NREL, Colorado State University, Fort Collins, CO, USA;

555. Ob/Gyn Department, Rzeszow University Hospital, Rzeszow, Poland;

556. Faculty of Biological Sciences, University of Leeds, UK;

557. Endocrine Unit, Massachusetts General Hospital, Boston, MA, USA;

558. Neuroscience, University of Oldenburg, Germany;

559. Microbiome Research Centre, St George & Sutherland Clinical School, UNSW Sydney, NSW,z Australia;

560. Departments of Biochemistry & Molecular Pharmacology, New York University School of Medicine, NY, USA;

561. Medicine/Infectious Diseases, University of Mississippi Medical Center, Jackson, MS, USA;

562. Faculty of Social Sciences / Psychology, Tampere University, Finland;

563. Department of Biological Sciences, University of Cyprus, Nicosia, Cyprus;

564. Institute of Medical Microbiology and Infection Control, Goethe University, Frankfurt, Germany;

565. Institute for Radiology, Charité Medical University of Berlin, Germany;

566. Internal Medicine I, University Hospital Cologne, University of Cologne, Germany;

567. Section of Periodontics, School of Dentistry, University of California Los Angeles, CA, USA;

568. Department of Clinical Biochemistry, Aalborg University Hospital, Denmark;

569. Pharmacy Practice, Manipal Academy of Higher Education, India;

570. Department of Radiology, Institute of Medical Science, The University of Tokyo, Japan;

571. Family Medicine, Faculty of Medicine, Çukurova University, Adana, Turkey;

572. Department of Humanities and Social Medicine, College of Medicine, The Catholic University of Korea;

573. Food Technology, Safety and Health, Ghent University, Belgium;

574. School of Biological, Earth and Environmental Sciences (BEES), UNSW Sydney, NSW, Australia;

575. Research Unit, St. Vincent Shoulder & Sports Clinic Vienna, Austria;

576. Biomedical Engineering, Cornell University, Ithaca, NY, USA;

577. Research Group Bioinformatics And Information Technology, Leibniz Institute of Plant Genetics and Crop Plant Research (IPK) Gatersleben, Germany;

578. School of Doctoral Studies, Universidad Europea de Madrid, Spain;

579. Biomedical Manufacturing, CSIRO Manufacturing, Melbourne, VIC, Australia;

581. Biological Sciences, DePaul University, Chicago, IL, USA;

582. Department of Animal Science and Technology, Konkuk University, Seoul, Korea;

583. Graduate Institute of Medical Mechatronics, Chang Gung University, Taoyuan, Taiwan;

584. Psychiatry, College of Medicine, Korea University, Seoul, Korea;

585. Chemistry, Princeton University, NJ, USA;

586. Henan Key Laboratory of Chinese Medicine for Respiratory Disease, Henan University of Chinese Medicine, Henan, China;

587. Department of Psychiatry and Psychotherapy, University Medical Center Mainz, Germany;

588. School of Science, Monash University Malaysia, Selangor, Malaysia;

589. Physiology and Biophysics, University of Mississippi Medical Center, Jackson, MS, USA;

590. Department of Transplantation Medicine, University of Oslo, Norway;

591. Triticeae Research Institute, Sichuan Agricultural University, Sichuan, China;

592. Gastroenterology, Guangzhou University of Chinese Medicine, Guangzhou, China;

593. School of Biological Science & Medical Engineering, Southeast University, Jiangsu, China;

594. Biology, Mount Allison University, Sackville, NB, Canada;

595. Biology, Ithaca College, Ithaca, NY, USA;

596. Department of medical Sciences and Public Health, University of Cagliari, Monserrato, Italy;

597. Chromosome Biology, University of Vienna, Austria;

598. Nephrology, University of Zürich, Switzerland;

599. Institute of Nutritional Sciences, Friedrich Schiller University, Jena, Germany;

600. Graduate School of Biomedical Engineering, UNSW Sydney, NSW, Australia;

601. Biochemistry and Molecular Biology, Tulane University School of Medicine, New Orleans, LA, USA;

602. National Institute of Neurological Disorders and Stroke, National Institutes of Health, Bethesda, MD, USA;

603. NCBI, NLM, National Institutes of Health, Bethesda, MD, USA;

604. L'Oréal Research and Innovation, Aulnay sous Bois, France;

605. Department of Psychology, Lund University, Malmö, Sweden;

606. Institut de Recherche Expérimentale et Clinique, Université Catholique de Louvain, Brussels, Belgium;

607. Neuroscience Institute, Georgia State University, Atlanta, GA, USA;

608. Surgery, Ophthalmology, University of Melbourne, Parkville, VIC, Australia;

609. Centre for Ophthalmology and Visual Science, The University of Western Australia, Perth, WA, Australia;

610. Medical Physics, Faculty of Medicine, Iran University of Medical Sciences, Tehran, Iran;

611. Cellular Neurobiology, Salk Institute, La Jolla, CA, USA;

612. Fibrosis Research Group, Imperial College London, UK;

613. Genitourinary Medical Oncology, University of Texas MD Andersson Cancer Center, Houston, TX, USA;

614. GIGA-Neurosciences, University of Liege, Belgium;

615. Urology, School of Medicine, University of Crete, Greece;

616. Department of Clinical Pharmacology, Flinders Medical Centre, Flinders University, Adelaide, SA, Australia;

617. Bioinformatics, Max Planck Institute of Immunobiology and Epigenetics, Breisgau, Germany;

618. School of Psychology, Cardiff University, UK;

619. Chemical Engineering, Imperial College London, UK;

620. Clinical Sciences, Skane University Hospital, Lund University, Malmö, Sweden;

621. Department of Molecular and Clinical Medicine, Sahlgrenska Academy, Institution of Clinical Science;

622. School of Pharmacy and Biomedical Sciences, University of Central Lancashire, Preston, UK;

623. Psychiatry & Clinical Psychology, Hospital Universitario Doctor Peset, Valencia, Spain;

624. Genome Dynamics and Function, Centro de Biologia Molecular Severo Ochoa, Madrid, Spain;

625. Department of Intensive Care, Unviversity Hospital of Lille, France;

626. Surgery, Kansai Medical University, Osaka, Japan;

627. Laboratory of Genomics and Biotechnology of Fruit, École Nationale Supérieure Agronomique de Toulouse, Institut National Polytechnique de Toulouse, Université de Toulouse, France;

628. Institute for Psychology, UiT - The Arctic University of Norway, Tromstø, Norway;

629. Centre for Public Health, Queen’s University Belfast, UK;

630. Centre for Primary Care and Health Services Research, University of Manchester, UK;

631. Menzies Health Institute, Griffith University, Gold Coast, QLD, Australia;

632. FHSCE, Anglia Ruskin University, Cambridge, UK;

633. Department of Experimental Medicine, Universitat de Lleida, Spain;

634. Biomolecular Screening Branch, Division National Toxicology Program, National Institute of Environmental Health Sciences, NC, USA;

635. Immunology and Microbial Disease, Albany Medical College, NY, USA;

636. Queensland Brain Institute, The University of Queensland, Brisbane, QLD, Australia;

637. School of Biosciences, University of Kent, UK;

638. LNC, UMR 1231, Inserm, Université Bourgogne Franche-Comté, Cedex, France;

639. Department of Biotechnology, College of Life Sciences, Ritsumeikan University, Shiga, Japan;

640. Biological Sciences, Kent State University, OH, USA;

641. Department of Safety Research on Blood and Biological Product, National Institute of Infectious Diseases, Tokyo, Japan;

642. Laboratory of Microbiology of Extreme Environments, European Institute for Marine Studies, Plouzané, France;

643. Department of Pharmacology, Roy J. and Lucille A. Carver College of Medicine, University of Iowa, Iowa City, IA, USA;

644. Biological Sciences, National University of Singapore;

645. Laboratoire GBA, EA4627, Conservatoire National des Arts et Métiers, Paris, France;

646. Department of Human Genetics, University of Michigan, MI, USA;

647. Belozersky Institute of Physico-Chemical Biology, Lomonosov Moscow State University, Russia;

648. Department of Molecular Biology, The Jikei University School of Medicine, Tokyo, Japan;

649. Genomics & Computational Biology, University of South Wales, Treforest, UK;

650. Obstetrics and Gynecology, Duke University Medical Center, Durham, NC, USA;

651. Climate Change Cluster, University of Technology Sydney, NSW, Australia;

652. Department of Radiology, Nagoya University Graduate School of Medicine, Aichi Prefecture, Japan;

653. School of Science and Health, Western Sydney University, NSW, Australia;

654. Department of Speech and Language Therapy, TEI of Epirus, Ioannina, Greece;

655. Orthopaedic Surgery, Indiana University-Purdue University, IN, USA;

656. Veterinary Pathology, Oniris, Nantes, France;

657. Pathobiology and Population Sciences, Royal Veterinary College, Hertfordshire, UK;

658. Lab of Pharmaceutical Biotechnology, Ghent University, Belgium;

659. Terrestrial Ecology, Norwegian Institute for Nature Research, Trondheim, Norway;

660. Molecular and Cell Biology, University of California Merced, CA, USA;

661. Centre for Transport Research, School of Engineering, Trinity College Dublin, The University of Dublin, Ireland;

662. Nephrology, Institution of Clinical Sciences, Lund University, Malmö, Sweden;

663. Mechanical Engineering, University of Birmingham, UK;

664. OB/GYN, Institution of Clinical Sciences, Lund University, Lund, Sweden;

665. Nephrology and Hypertension, Fundación Jiménez Diaz Hospital, Madrid, Spain;

666. School of Chemistry, Physics and Mechanical Engineering, Queensland University of Technology, Brisbane, QLD, Australia;

667. Psychology, Otto-von-Guericke-University, Magdeburg, Germany;

668. Department of Experimental Neurodegeneration, University Medical Center Göttingen, Germany;

669. Physical Medicine and Rehabilitation, Harvard Medical School, Spaulding Rehabilitation Hospital, Boston, MA, USA;

670. Scientific Operations, Quadram Institute Bioscience, Norwich, UK;

671. Regensburg Medical Image Computing (ReMIC), Ostbayerische Technische Hochschule Regensburg (OTH Regensburg), Germany;

672. Faculty of Arts and Education, Deakin University, Melbourne, VIC, Australia;

673. Warwick Medical School, University of Warwick, Coventry, UK;

674. Biochemistry & Molecular Biology, National Institute for Health and Medical Research (INSERM), Paris, France;

675. Tax Institute, Université de Liège, Belgium;

676. School of Molecular and Cellular Biology, University of Leeds, UK;

677. UOC Genetica Medica, IRCCS Istituto Giannina Gaslini, Genova, Italy;

678. Science, Research Diets, Inc., New Brunswick, NJ, USA;

679. Department of Physics and Geology, University of Perugia, Italy;

680. Cellular Immunology, Walter Reed National Military Medical Center, Bethesda, MD, USA;

681. Microbiology, Immunology and Parasitology Departament, Biological Science Center, Federal University of Santa Catarina, Brazil;

682. Centre for Liver and Digestive Disorders, The Royal Infirmary, University of Edinburgh, UK;

683. Critical Care Proprietary Products Division, Orion Pharma, Espoo, Finland;

684. Department of Civil and Environmental Engineering, Massachusetts Institute of Technology, Cambridge, MA, USA;

685. Department of Veterinary Sciences, University of Turin, Italy;

686. Dept. Psychological, Health and Territorial Sciences, University of Chieti, Italy;

687. OB-GYN, New York University School of Medicine, New York, NY, USA;

688. Institute of Cardiovascular and Medical Sciences, University of Glasgow, UK;

689. Department of Biology, University of Turku, Finland;

690. Bioanalytics, Technische Universitaet Berlin, Germany;

691. University of Goettingen, Third Institute of Physics - Biophysics, Germany;

692. Translational Research to Advance Therapeutics and Innovation in Oncology (TRACTION), Institute for Applied Cancer Science, The University of Texas MD Anderson Cancer Center, Houston, TX, USA;

693. Life Sciences, University of Liège, Belgium;

694. Department of Toxicology, Faculty of Medical Sciences, Tarbiat Modares University, Tehran, Iran;

695. Agricultural Research Service, USDA, Stoneville, MS, USA;

696. RCI Regensburg Center of Interventional Immunology, University of Regensburg, Germany;

697. School of Psychology, University of Nottingham, UK;

698. Laboratory of Pathology, National Institutes of Health, Bethesda, MD, USA;

699. Electrical Engineering, Universidad Carlos III de Madrid, Spain;

700. Chemical Biology, Instituto de Medicina Molecular, Lisbon, Portugal;

701. CIET, University of Costa Rica;

702. Faculty of Health Sciences, University of Stavanger, Norway;

703. Urology, Erasmus University Medical Center, Rotterdam, Netherlands;

704. School of Biological Sciences, University of Edinburgh, UK;

705. Institute for Bioinformatics and Systems Biology (IBIS), Helmholtz Zentrum München-German Research Center for Environmental Health (GmbH), Ingolstädter Landstraße 1, D-85764 Neuherberg, Germany;

706. Huygens-Kamerlingh Onnes Laboratory, Leiden University, Netherlands;

707. Nutritional Sciences, University of Vienna, Austria;

708. Medicine, Kolling Institute of Medical Research, St Leonards, NSW, Australia;

709. Biological Chemistry, Johns Hopkins School of Medicine, Baltimore, MD, USA;

710. Medicine / Nutrition and Microbiome Laboratory, Universite de Montreal, Montreal, QC, Canada;

711. Cell and Gene Therapy, GlaxoSmithKline, Stevenage, UK;

712. Life Sciences, University of Trieste, Italy;

713. Department of Radiology, RWTH Aachen University, Aachen, Germany;

714. Department of Medical Oncology, West German Cancer Center, University Hospital Essen, University of Duisburg-Essen, Germany;

715. Obstetrics & Gynecology, Medical University of Vienna, Austria;

716. Social and Behavioral Health Sciences Division, FHI 360, Washington, DC, USA;

717. VIB Switch Laboratory, VIB-KU Leuven, Belgium;

718. Faculty of Technology, Bielefeld University, Germany;

719. Department of Gastrointestinal Surgery, Department of Clinical Nutrition, Beijing Shijitan Hospital, Capital Medical University, Beijing, China;

720. Department of Agricultural Chemistry, Meiji University, Kawasakishi, Japan;

721. Microbiology, Yonsei University College of Medicine, Seoul, Korea;

722. Johnson & Johnson EAME, Maidenhead, UK;

723. Pediatrics, Penn State College of Medicine, PA, USA;

724. Department of Social Medicine, University of North Carolina at Chapel Hill, NC, USA;

725. ARC CoE Plant Energy Biology, The University of Western Australia, Perth, WA, Australia;

726. Division of Human Nutrition and Health, Wageningen University, Wageningen, Netherlands;

727. Department of Neuroimaging, King’s College London, UK;

728. Biochemistry & Molecular Biology, University of Murcia, Spain;

729. Department of Biological Sciences, Old Dominion University, Norfolk, VA, USA;

730. Biochemistry and Molecular Biology, Monash Univerity, Melbourne, VIC, Australia;

731. Institute of Genetics and Developmental Biology, Chinese Academy of Sciences, Beijing, China;

732. Pharmacology & Chemical Biology, University of Pittsburgh, PA, USA;

733. Center for Integrative Genomics, University of Lausanne, Switzerland;

734. School of Pharmacy, The University of Queensland, Brisbane, QLD, Australia;

735. Genebank, Leibniz Institute of Plant Genetics and Crop Plant Research (IPK) Gatersleben, Germany;

736. Research and Development, Piramal Imaging, Berlin, Germany;

737. Civil Engineering, University of Leeds, UK;

738. Chemistry and Biochemistry, University of Missouri St. Louis, MO, USA;

739. Coastal and Marine Geology Program, US Geological Survey, USGS Pacific Coastal and Marine Science Center, Santa Cruz, CA, USA;

740. Ajinomoto-Genetika Research Institute, Moscow, Russia;

741. Department of Plant Biotechnology, Faculty of Biochemistry, Biophysics and Biotechnology, Jagiellonian University, Kraków, Poland;

742. Engineering Science and Materials, University of Puerto Rico, Mayagüez, PR, USA;

743. Department of Chemistry and Biochemistry, University of Regina, SK, Canada;

744. Center for Nanoscale Materiaals, Argonne National Laboratory, IL, USA;

745. Sunshine Coast Mind & Neuroscience - Thompson Institute, University of the Sunshine Coast, QLD, Australia;

746. Department of Gastroenterology and Hepatology, The Chinese PLA General Hospital, Beijing, China;

747. Griffith Centre for Social and Cultural Research, Griffith University, Gold Coast, QLD, Australia;

748. Department of Molecular Oncology, Inst Dev Aging Cancer, Tohoku University, Sendai, Japan;

749. Computer Science, Indiana University, Bloomington, IN, USA;

750. Department of Pulmonary Medicine, Fukushima Medical University School of Medicine, Fukushima, Japan;

751. Biology, MEDIVIR AB, Huddinge, Sweden;

752. Neuroimmunology, Western Sydney University, NSW, Australia;

753. Nutrition and Food Technology, The University of Jordan;

754. Institute of Biodiversity, Federal Research Center for Rura Areas, Forestry and Fisheries, Thünen Institute of Forest Genetics, Großhansdorf, Germany;

755. Institute of Cell Biology and the Centre for Integrative Physiology, University of Edinburgh, UK;

756. Structural Genomics Consortium, University of Toronto, ON, Canada;

757. Graduate Education and Research, Canadian Memorial Chiropractic College, Toronto, ON, Canada;

758. R&D, Agilent Technologies, Leuven, Belgium;

759. School of Nursing, The University of British Columbia, Vancouver, BC, Canada;

760. Stroke and Ageing Research, Department of Medicine, School of Clinical Sciences at Monash Health, Monash Univerity, Melbourne, VIC, Australia;

761. Biology, Transfer, Innovations, French National Cancer Institute, Boulogne-Billancourt, France;

762. NanoBioSciences, Physical Chemistry, Ludwig Maximilians-Universität München, Germany;

763. Medicinal Chemistry, School of Pharmacy, Bandung Institute of Technology, Indonesia;

764. Life Sciences Research Unit, University of Luxembourg;

765. Department of Gastroenterology, Skane University Hospital, Lund University, Malmö, Sweden;

766. Translational Genetics, Millennium Health, San Diego, CA, USA;

767. Clinical Research and Evidence-Based Medicine Unit, Second Medical Department, Aristotle University Thessaloniki, Greece;

768. Department of Psychology, The Jan Kochanowski University in Kielce, Piotrków Trybunalski Branch, Poland;

769. Engineering Physics, McMaster University, Hamilton, ON, Canada;

770. Department of Agriculture, Food and Environmental Sciences, Marche Polytechnic University, Ancona, Italy;

771. Microbiology, Faculty of Medicine, Kuwait University;

772. Department of Breast Surgery, Fujita Health University, Aich, Japan;

773. Spinal Injuries Centre, North West Regional Spinal Injuries Centre, Merseyside, UK;

774. Competence Centre in Methodology and Statistics, Luxembourg Institute of Health;

775. Metabolic Health, Nestle Institute of Health Sciences SA, Écublens, Vaud, Switzerland;

776. VIB Center for Inflammation Research, Ghent, Belgium and Department of Biomedical Molecular Biology, Ghent University, Ghent, Belgium;

777. Currículo, formaÇão de professores e tecnologia, Instituto de EducaÇão da Universidade de Lisboa, Portugal;

778. Centre for Inflammation Research, University of Edinburgh, UK;

779. School of BioSciences, University of Melbourne, Parkville, VIC, Australia;

780. Computer and Information Sciences, Northumbria University, Newcastle upon Tyne, UK;

781. Endocrinology, University of Valencia, Spain;

782. INRS-Institut Armand-Frappier, INRS, Laval, QC, Canada;

783. School of Nutrition, INAF, Université Laval, Québec City, QC, Canada;

784. Department of Biology, University of Konstanz, Konstanz, Germany;

785. Université Côte d’Azur, LAMHESS, France;

786. Systems Ecology, Scion, Christchurch, New Zealand;

787. Epidemiology and Biostatistics, CUNY Graduate School of Public Health and Health Policy, NY, USA;

788. School of Dentistry, The University of Queensland, Brisbane, QLD, Australia;

789. The Renal and Metabolic Division, The George Institute for Global Health, Sydney, NSW, Australia;

790. College of Chemistry & Molecular Sciences, Wuhan University, Hubei, China;

791. School of Environment and Science, Griffith University, Gold Coast, QLD, Australia;

792. Radiation Oncology, University of Minnesota, Minneapolis, MN, USA;

793. Faculty of Medicine, Goethe University, Frankfurt, Germany;

794. Department and Graduate School of Safety and Environment Engineering, National Yunlin University of Science & Technology, Taiwan;

795. School of Sport, Exercise and Nutrition, Massey University, Auckland, New Zealand;

796. Wildlife Ecology and Conservation, University of Florida, Gainesville, FL, USA;

797. Department of Psychology, Bournemouth University, Poole, UK;

798. Project Group P2, Robert Koch Institute, Berlin, Germany;

799. MRC Institute of Genetics and Molecular Medicine, University of Edinburgh, UK;

800. Biozentrum, University of Basel, Switzerland;

801. School of Medicine, University of Wollongong, Wollongong, NSW, Australia;

802. Institute of Human Genetics, University of Cologne, Germany;

803. Rural Economics Branch, Economic Reserach Service, Washington, DC, USA;

804. Institut de Neurosciences Cognitives et Intégratives d’Aquitaine, CNRS, Uiversity of Bordeaux, France;

805. Department of Chemical & Environmental Engineering and Materials Science & Engineering Program, University of California Riverside, CA, USA;

806. Department of Medicine, Mackay Medical College, New Taipei City, Taiwan;

807. Division of Animal Welfare, University of Bern, Switzerland;

808. Department of Physiology and Pharmacology, Oregon Health & Science University, Portland, OR, USA;

809. Institute of Interdisciplinary Medical Science, Shanghai University of Traditional Chinese Medicine, Shanghai, China;

810. Biology, McMaster University, Hamilton, ON, Canada;

811. Department of Cell Biology, Microbiology and Molecular Biology, University of South Florida, Tampa, FL, USA;

812. Medical Oncology, Hacettepe University Institute of Cancer, Ankara, Turkey;

813. Department of Electronic Engineering, City University of Hong Kong, Hong Kong, China;

814. Department of Entomology, National Taiwan University, Taipei, Taiwan;

815. Ecological Security, Institute of Environment and Sustainable Development in Agriculture, Chinese Academy of Agricultural Sciences, Beijing, China;

816. Chemistry and Biochemistry, Institute of Molecular Biophysics, Florida State University, Tallahasse, FL, USA;

817. Lab of Computational Chemistry and Drug Design, State Key Laboratory of Chemical Oncogenomics, Peking University Shenzhen Graduate School, Shenzhen, China;

818. Drug Research Program, Division of Pharmaceutical Chemistry and Technology, Faculty of Pharmacy, University of Helsinki, Finland;

819. Microbial Biotechnology, Tohoku University, Sendai, Japan;

820. Department of Pharmacology, School of Basical Medical Sciences, Tianjin Medical University, Tianjin, China;

821. Biostatistics and Computational Biology, Dana Farber Cancer Institute, Boston, MA, USA;

822. Institute of Molecular and Genomic Medicine, National Health Research Institutes, Taiwan;

823. Physiology Anatomy and Genetics, University of Oxford, UK;

824. Physics, George Washington University, Washington, DC, USA;

825. School of Biological Sciences, University of Nebraska-Lincoln, NE, USA;

826. Department of Laboratory Medicine and Pathobiology, Toronto General Hospital Research Institute, ON, Canada;

827. Biological Sciences, The University of Texas at Dallas, Richardson, TX, USA;

828. Department of Chemistry, New York University, NY, USA;

829. School of Mathematics and Statistics, Shandong University, Shandong, China;

830. Hefei National Laboratory for Physical Science at Microscale and School of Life Sciences, University of Science and Technology of China, Anhui, China;

831. Medicinal Chemistry and Molecular Pharmacology, Purdue University, West Lafayette, IN, USA;

832. Advanced Therapies, National Institute for Biological Standards and Control (NIBSC), Herts, UK;

833. Department of Pulmonary & Critical Care Medicine, Ruijin Hospital Affiliated to Shanghai Jiao Tong University, Shanghai, China;

834. Dermatology and Allergy, Charité

Medical University of Berlin, Germany;

835. Hematology, University Hospital of Saint-Etienne, France;

836. Institute of Biotechnology, Inland Norway University of Applied Sciences;

837. Pediatrics, The University of Jordan;

838. Molecular mycology unit, Institut Pasteur, Paris, France;

839. Medical Research Council Centre for Neuropsychiatric Genetics and Genomics, Institute of Psychological Medicine and Clinical Neurosciences, School of Medicine, Cardiff University, UK;

840. Social Work, Zurich University of Applied Sciences, Switzerland;

841. School of Life Sciences, Jawaharlal Nehru University, New Delhi, India;

842. LE2I, University of Burgundy - Franche-Comté, Dijon, France;

843. Life Sciences, University of Roehampton, London, England;

844. General and HPB Surgery, Ghent University Hospital, Belgium;

845. Insect-Fungus Symbiosis lab, University of Wuerzburg, Germany;

846. Behavioural Science Institute, Radboud University, Nijmegen, Netherlands;

847. Application center HOFZET, Fraunhofer WKI, Hannover, Germany;

848. Structural and Molecular Biology, University College London, UK;

849. Developmental Psychology, University of Amsterdam, Netherlands;

850. CNAP, SMI, Health Science and Technology, Aalborg University, Denmark;

851. Center for Health Equity Research and Promotion, VA Pittsburgh Healthcare System, Pittsburgh, PA, USA;

852. Neurosurgery, Cedars Sinai Medical Center, Los Angeles, CA, USA;

853. School of Data and Computer Science, Sun Yat-sen University, Guangzhou, Guangdong, China;

854. Shandong Provincial Key Laboratory of Biophysics, Dezhou University, Shandong, China;

855. UMR Espace-DEv, Institut de Recherche pour le Développement, Maison de la Télédétection, Montpellier, France;

856. School of Life Sciences, Xiamen University, Fujian, China;

857. Department of Medical Imaging, National Clinical Research Center of Kidney Diseases, Jinling Hospital, Nanjing University School of Medicine, Nanjing, China;

858. School of Social Policy, Sociology and Social Research, University of Kent, UK;

859. Infectious Diseases and Tropical Medicine, Federal University of Minas Gerais, Brazil;

860. Life and Health Sciences Research Institute (ICVS) - School of Medicine, University of Minho, Braga, Portugal;

861. Biochimie et Médecine Moléculaire, Universite de Montreal, Montreal, QC, Canada;

862. Epidemiology, Johns Hopkins Bloomberg School of Public Health, Baltimore, MD, USA;

863. Metabolic and Genetic Regulation of Ageing, Max Planck Institute for Biology of Ageing, Cologne, Germany;

864. Health Sciences, University of Swaziland;

865. Institute for Future Environments, Queensland University of Technology, Brisbane, QLD, Australia;

866. Département de Biologie Médicale, Centre Scientifique de Monaco;

867. Urology, HELIOS Hospital, Bad Saarow, Germany;

868. Institute of Microbiology, Technische Universität Braunschweig, Germany;

869. Fetal i+D Fetal Medicine Research Center, IDIBAPS BCNatal — Barcelona Center for Maternal Fetal and Neonatal Medicine, Hospital Clínic and Hospital Sant Joan de Déu, Universitat de Barcelona, Spain;

870. Institute of Bacterial Infections and Zoonoses, Friedrich Loeffler Institut, Jena, Germany;

871. Neurology, Charité

Medical University of Berlin, Germany;

872. Molecular Therapeutics for Cancer in Ireland, National Institute for Cellular Biotechnology, Dublin City University, Ireland;

875. Institute for Sex Research and Forensic Psychiatry, University Medical Center Hamburg Eppendorf, Germany;

876. School of Mathematical Sciences and LPMC, Nankai University, Tianjin, China;

877. Oncology, University of Oxford, UK;

878. Classics, Royal Holloway, Egham, UK;

879. Clinical Sciences, Cornell University, Ithaca, NY, USA;

880. Pharmaceutical Chemistry, University of KwaZulu-Natal, Westville Campus, Durban, South Africa;

881. Medicine, Royal College of Surgeons in Ireland, Dublin, Ireland;

882. Department of Immunology, University of Oslo, Norway;

883. Marine Nitrogen Cycling Lab, Bermuda Institute of Ocean Sciences, St. George’s, Bermuda;

884. Institute of Liberal Arts and Sciences, Kanazawa University, Japan;

885. World Health Organization Regional Office for Africa, Brazzaville, Congo;

886. Infection Control Department, University Hospital of BesanÇon, France;

887. Clinical Development, Galapagos NV, Mechelen, Belgium;

888. Integrated Marine Observing System, University of Tasmania, Hobart, TAS, Australia;

889. Department of Systematics, Biodiversity and Evolution of Plants, Albrecht-von-Haller Institute for Plant Sciences, Georg August University of Göttingen, Germany;

890. Department of Psychiatry, University of Occupational and Environmental Health, Fukuoka, Japan;

891. Program of Precision Nutrition and Aging, IMDEA-Food, Madrid, Spain;

892. IQHealthcare, Radboud University Medical School, Netherlands;

893. Department of Cardiovascular Surgery, Maastricht University, Maastricht, Netherlands;

894. Institute for Clinical Biochemistry and Pathobiochemistry, German Diabetes Center, Düsseldorf, Germany;

895. Department of Radiology, Juntendo University Graduate School of Medicine, Tokyo, Japan;

896. School of Information Technology, Deakin University, Melbourne, VIC, Australia;

897. Department of Interface Chemistry and Surface Science, Max-Planck-Institut fur Eusenforschung, Dusseldorf, Germany;

898. Department of Psychology, Edge Hill University, Ormskirk, UK;

899. Psychiatry, Aga Khan University, Karachi, Pakistan;

900. Korean Bioinformation Center, Korea Research Institute of Bioscience and Biotechnology (KRIBB);

901. Health Economics and Outcomes Research, Cardinal Health Specialty Solutions, Dallas, TX, USA;

902. Division of Clinical Pharmacology, Klinikum der Universität München, Germany;

903. Neurological Surgery, University of Pittsburgh, PA, USA;

904. Department of Child and Adolescent Psychiatry, Psychosomatics, and Psychotherapy, RWTH Aachen University, Aachen, Germany;

905. Institute of Molecular and Cellular Biology, University of Copenhagen, Denmark;

906. Structural Biology, St. Jude Children’s Research Hospital, Memphis, TN, USA;

907. Colorectal Surgery, Royal Shrewsbury Hospital, UK;

908. Faculty of Medicine & Health Sciences, University of Nottingham, UK;

909. Department of Physiology and Pharmacology, Karolinska Institutet, Solna, Sweden;

910. State Key Lab of Electroanalytical Chemistry, Changchun Institute of Applied Chemistry, Chinese Academy of Sciences, Jilin, China;

911. Pediatrics, The University of British Columbia, Vancouver, BC, Canada;

912. Cotton Germplasm Resources, Research Base in Anyang Institute of Technology, State Key Laboratory of Cotton Biology/Institute of Cotton Research, Chinese Academy of Agricultural Sciences, Beijing, China;

913. Anaesthesia and Intensive care, The Chinese University of Hong Kong, Hong Kong, China;

914. ICMS, University of Macau, Guangdong, China;

915. School of Renewable Energy, North China Electric Power University, Beijing, China;

916. Department of Internal Medicine, Justus-Liegbig University, Giessen, Germany;

917. Bioscience, Aarhus University, Denmark;

918. The Irish Longitudinal Study on Ageing (TILDA), Trinity College Dublin, The University of Dublin, Ireland;

919. Hematology, University Medical Center Groningen, University of Groningen, Netherlands;

920. Child Neurology, VU University Medical Center, Amsterdam, Netherlands;

921. European Molecular Biology Laboratory, European Bioinformatics Institute (EMBL-EBI), Cambridge, UK;

922. HGF MPG Joint Research Group for Deep-Sea Ecology and Technology, Max Planck Institute for Marine Microbiology, Bremen, Germany;

923. Center for Adaptive Rationality, Max Planck Institute for Human Development, Berlin, Germany;

924. Mathematics, King Faisal University, Hofuf, Saudi Arabia;

925. School of Nursing & Midwifery, Griffith University, Gold Coast, QLD, Australia;

926. The Roy J. Carver Department of Biochemsitry, Biophysics and Molecular Biology, Iowa State University, Ames, IA, USA;

927. Engineering Thermodynamics, Process & Energy Department, Faculty of Mechanical, Maritime and Materials Engineering, Delft University of Technology, Leeghwaterstraat 39, 2628CB, Delft, The Netherlands;

928. Cytotechnology Education, College of Allied Health Professions, University of Nebraska Medical Center, Omaha, Nebraska, USA;

929. Department of Cardiovascular Medicine, Shinko Memorial Hospital, Kobe, Japan;

930. Materials, Imperial College London, UK;

931. Department of Surgery, Technical University of Munich, München, Germany;

932. Laboratory of Quality and Safety Risk Assessment for Fruit (Xingcheng), Ministry of Agriculture, Research Institute of Pomology, Chinese Academy of Agricultural Sciences, Liaoning, China;

933. Communication Sciences and Disorders, James Madison University, Harrisonburg, VA, USA;

934. Institute for Orthopaedic Research and Biomechanics, University Hospital Ulm, Germany;

935. School of Health and Social Care, University of Essex, UK;

936. Research Services, Alpha-Altis, Nottingham, UK;

937. Medical Oncology, Erasmus University Medical Center, Rotterdam, Netherlands;

938. Department of Industrial Chemistry, Federal University Oye, Ekiti, Nigeria;

939. Cell Biology, Duke University Medical Center, Durham, NC, USA;

940. Nuffield Department of Clinical Neurosciences, University of Oxford, UK;

941. Cancer Research UK Manchester Institute, University of Manchester, UK;

942. Children’s Hospital, Helsinki University Hospital, Finland;

943. Department of Biology, CESAM - Centre for Environmental and Marine Studies, University of Aveiro, Portugal;

944. Psychology, University of Botswana, Gaborone, Botswana;

945. Nursing School, Federal University of Bahia, Canela, Salvador-Bahia;

946. Biological and Experimental Psychology, Queen Mary University of London, UK;

947. Medicinal Chemistry Department, National University of Pharmacy, Kharkiv, Ukraine;

948. Department of Education and Psychology, University of Bolton, UK;

949. Department of Chemistry and Physics, La Trobe University, Melbourne, VIC, Australia;

950. Department of Gastroenterology, General Hospital of Northern Theater Command, Liaoning, China;

951. Department of Nephrology, Doctors Hospital Athens, Greece;

952. Pediatrics III, University Hospital Essen, Germany;

953. Infectious Disease Epidemiology, Imperial College London, UK;

954. Department of Psychiatry, Sorbonne Universite, Paris France;

955. Education, UNSW Sydney, NSW, Australia;

956. Department of Psychiatry & Behavioral Sciences, Stanford University, Palo Alto, CA, USA;

957. Clinic for Laryngology, Rhinology and Otology, Hannover Medical School, Germany;

958. Centre for Aboriginal Studies, Curtin University, Perth, WA, Australia;

959. Biomedical Engineering Department, Iran University of Science and Technology, Tehran, Iran;

960. Anesthesiology, University of California San Francisco, CA, USA;

961. Mechanical Engineering, Khalifa University of Science and Technology, Abu Dhabi, UAE;

962. Horticultural Sciences, University of Florida, Gainesville, FL, USA;

963. Centre for Biodiscovery and Molecular Development of Therapeutics, Australian Institute of Tropical Health and Medicine, James Cook University, Cairns, QLD, Australia;

964. CINTESIS, Faculty of Medicine, University of Porto, Portugal;

965. Medical Research Center, Shaoxing People’s Hospital, Zhejiang, China;

966. Department Transfusion Medicine, National Institutes of Health, Bethesda, MD, USA;

967. Department of Biotechnology, All India Institute of Medical Sciences (AIIMS), New Delhi, India;

968. Biochemistry, Microbiology and Immunology, University of Ottawa, ON, Canada;

969. Division of Mental Health and Addiction, Institute of Clinical Medicine, University of Oslo, Norway;

970. Department of Radiology, University Medical Center Groningen, University of Groningen, Netherlands;

971. School of Nursing, The University of Hong Kong, Hong Kong, China;

972. Urology, Tokyo Medical University Ibaraki Medical Center, Japan;

973. Department of Radiation Oncology, University Hospital Zürich, Switzerland;

974. Division of Immunotherapy, Institute of Human Virology and Department of Surgery, University of Maryland, Baltimore, MD, USA;

975. Division of Molecular Biology and Human Genetics, Faculty of Medicine and Health Sciences, Stellenbosch University, South Africa;

976. College of Biological Sciences, China Agricultural University, Beijing, China;

977. Graduate School of Pharmaceutical Sciences, Osaka University, Japan;

978. Biochemistry, University of Washington, Seattle, WA, USA;

979. Institute of Bioscience and BioResources, National Research Council of Italy, Naples, Italy;

980. Physics, Université de Lyon, France;

981. Center for Social Psychology, Faculty of Psychology, University of Basel, Switzerland;

982. Centre for Molecular Oncology, Barts Cancer Institute, Queen Mary University of London, London, UK;

983. Samples, Phenotypes & Ontologies Team, European Bioinformatics Institute (EMBL-EBI), Cambridge, UK;

984. Faculty of Arts and Education, Charles Sturt University, Bathurst, NSW, Australia;

985. Helmholtz Institute of Biotechnology, State Key Laboratory of Microbial Technology, School of Life Sciences, Shandong University, Shandong, China;

986. Department of Biology, Shantou University, Guangdong, China;

987. Institute of Biomedical Sciences, Shanxi University, Shanxi, China;

988. Computational Biology, St. Jude Children’s Research Hospital, Memphis, TN, USA;

989. College of Bioinformatics Science and Technology, Harbin Medical University, Heilongjiang, China;

990. Radiology and Imaging Sciences, National Institutes of Health, Bethesda, MD, USA;

991. Department of Biological Sciences, Georgia Institute of Technology, Atlanta, GA, USA;

992. XtalPi Inc., Cambridge, MA, USA;

993. Bioinformatics, Instituto de Investigación en Biomedicina de Buenos Aires (IBioBA) - CONICET - Partner Institute of the Max Planck Society, Buenos Aires, Argentina;

994. Save Sight Institute, University of Sydney, NSW, Australia;

995. Australian Centre for Precision Health, University of South Australia Cancer Research Institute, University of South Australia, Adelaide, SA, Australia;

996. Key Laboratory of Functional Protein Research of Guangdong Higher Education Institutes, Institute of Life and Health Engineering, Jinan University, Guangdong, China;

997. Epidemiology, Human Genetics and Environmental Sciences, The University of Texas Health Science Center at Houston, TX, USA;

998. Department of Microbiology and Immunology, Weill Cornell Medicine, NY, USA;

999. Biotechnology Laborary, Guangdong Institute of Applied Biological Resources, Guangdong, China;

1000. College of Life Science, Shandong Normal University, Jinan, China;

1001. Life Sciences Department, Shandong University, Shandong, China;

1002. Integrative Microbiology Research Centre, South China Agriculture University, Guangzhou, Guangdong, China;

1003. Crop Molecular Improving Laboratory, Liaoning Academy of Agricultural Sciences, Liaoning, China;

1004. Medical Biophysics, Lawson Health Research Institute, London, ON, Canada;

1005. Infrastructure Engineering, University of Melbourne, Parkville, VIC, Australia;

1006. Faculty of Health, University of Canberra, ACT, Australia;

1007. MRC Cognition & Brain Sciences Unit, University of Cambridge, UK;

1008. Biostatistics and Bioinformatics, Emory University, Atlanta, GA, USA;

1009. Anesthesiology and Critical Care Medicine, Johns Hopkins School of Medicine, Baltimore, MD, USA;

1010. The School of Animal Rural and Environmental Sciences, Nottingham Trent University, Nottingham, UK;

1011. Biosciences, University of Exeter, UK;

1012. Hillingdon Hospitals NHS Foundation Trust, London, UK;

1013. MRC/CSO Social and Public Health Sciences Unit, University of Glasgow, UK;

1014. ICU Evaggelismos Athens Hospital, National and Kapodistrian University of Athens, Greece;

1015. Biological Sciences, The University of Newcastle, Callaghan, NSW, Australia;

1016. Centre for Innovative Research Across the Life Course, Faculty of Health and Life Sciences, Coventry University, UK;

1017. Service of Endocrinology, Diabetes and Metabolism, Lausanne University Hospital, Switzerland;

1018. School of Community Health, Charles Sturt University, Bathurst, NSW, Australia;

1019. Institute for Global Food Security, Queen’s University Belfast, UK;

1020. Institute of Policy Studies, National University of Singapore;

1021. Intitute doe Medicine and Engineering, University of Pennsylvania, Philadelphia, PA, USA;

1022. Cold Spring Harbor Laboratory, NY, USA;

1023. EECS, University of Michigan, MI, USA;

1024. Centre for Blood Research, The University of British Columbia, Vancouver, BC, Canada;

1025. Faculty of Health Sciences, Department of Health and Care Sciences, UiT - The Arctic University of Norway, Tromsø, Norway;

1026. Physiotherapy, Hospital of Clinics of Porto Alegre, Brazil;

1027. Medical school, University Paris Descartes, Paris, France;

1028. Institute of Crop Sciences, National Key Facility for Crop Gene Resources and Genetic Improvement, Chinese Academy of Agricultural Sciences, Beijing, China;

1029. Experimental-Clinical and Health Psychology, Ghent University, Belgium;

1030. Bioinformatics & Structural Biology, Indian Institute of Advanced Research, Gujart, India;

1031. Laboratory of Molecular Medicine, Bambino Ges. Children’s Research Hospital, Rome, Italy;

1032. Center of Infectious Disease - Parasitology, University of Heidelberg, Germany;

1033. Electrical Engineering, Stanford University, Palo Alto, CA, USA;

1034. Biology, University of Cádiz, Andalusia, Spain;

1035. general surgery, Mansoura University Hospital, Egypt;

1036. Virology pole, Institut Pasteur de Dakar, Senegal;

1037. Division of Cancer & Genetics, Cardiff University, UK;

1038. Food Safety and Environmental Microbiology, Centre of Expertise and Biological Diagnostic of Cameroon, Yaoundé, Cameroon;

1039. Laboratory of Thin Films and Photovoltaics, Swiss Federal Laboratories for Materials Science and Technology, Dübendorf, Switzerland;

1040. Assiut Urology and Nephrology Hospital, Faculty of Medicine, Assiut University, Assiut, Egypt;

1041. GEE and IHA, University College London, UK;

1042. University of Derby Online Learning, University of Derby, UK;

1043. Family, Population and Preventive Medicine, Stony Brook University, NY, USA;

1044. Molecular Medicine Division, Walter and Eliza Hall Institute, Melbourne, VIC, Australia;

1045. Northern Institute for Cancer Research, Newcastle University, Newcastle upon Tyne, UK;

1046. Clinical Dementia Research, German Center for Neurodegenerative Diseases, Bonn, Germany;

1047. UMR 7144, Station Biologique, Sorbonne Université, CNRS, France;

1048. Odontoestomatologia, University of Barcelona, Spain;

1049. Computer Science, Janelia Research Campus, Ashburn, VA, USA;

1050. Centre for Tropical Medicine and Global Health, University of Oxford, UK;

1051. ARTORG Center for Biomedical Engineering Research, University of Bern, Switzerland;

1052. Eccles Institute of Neuroscience, John Curtain School of Medical Research, The Australian National University, Canberra, ACT, Australia;

1053. Metabolic Biology, John Innes Centre, Norwich, UK;

1054. Genomics and Bioinformatics Research Unit, USDA Agricultural Research Service, Raleigh, NC, USA;

1055. Holzinger Group, Institute for Medical Informatics, Statistics and Documentation, Medical University Graz, Austria;

1056. Pharmacy, Ajou University, Suwon, Korea;

1057. School of Energy and Environment, City University of Hong Kong, Hong Kong, China;

1058. School of Kinseiology, The University of British Columbia, Vancouver, BC, Canada;

1059. Department of Biology, Marine Biology Section / University of Copenhagen, Denmark;

1060. Department of Communication, University of Vienna, Austria;

1061. School of Social Sciences, University of Dundee, UK;

1062. Institute of Botany, Technische Universitat Dresden, Germany;

1063. Division of Structural Biology, University of Oxford, UK;

1064. Medicine, National University Health System, Singapore;

1065. School of Biological Sciences, University of Canterbury, Christchurch, New Zealand;

1067. Primary Care Health Sciences, University of Oxford, UK;

1068. National Institute of Neurological Disorders and Stroke (NINDS), National Institutes of Health, Bethesda, MD, USA;

1069. Verna and Marrs McLean Department of Biochemistry and Molecular Biology, Baylor College of Medicine, Houston, TX, USA;

1070. Department of Public Health Nursing, Faculty of Nursing, Adnan Menderes University Aydin, Turkey;

1071. Department of Neurology IC, Oasi Research Institute - IRCCS, Troina, Italy;

1072. Weldon School of Biomedical Engineering, Purdue University, West Lafayette, IN, USA;

1073. King’s Centre for Military Health Research, King’s College London, UK;

1074. Department of Infectious Diseases Epidemiology, LSHTM, London, UK;

1075. BMWZ - Organic chemistry, University Hannover, Germany;

1076. Department of Physiology and Pathophysiology, School of Basic Medical Sciences, Xi’an Jiaotong University, Shaanxi, China;

1077. School of Pharmacy and Medical Sciences, University of South Australia, Adelaide, SA, Australia;

1078. Department of Physical Education, Federal University of Santa Catarina, Florianópolis, Brazil;

1079. Department of Oncology, Nanfang Hospital, Southern Medical University, Guangzhou, China;

1080. Hansen Experimental Physics Laboratory, Stanford University, Palo Alto, CA, USA;

1081. Institute of Translational Medicine, Shenzhen Second People’s Hospital, The First Affiliated Hospital of Shenzhen University, Guangdong, China;

1082. Department of Statistics and Actuarial Science, The University of Hong Kong, Hong Kong, China;

1083. Department of Mechanical Engineering, University College London, UK;

1084. Laboratory of Microbial Immunity, Singapore Immunology Network, Agency for Science, Technology and Research (A*STAR), Singapore;

1085. State Key Laboratory of Powder Metallurgy, Central South University, Hunan, China;

1086. Institute of Applied Health Sciences, University of Aberdeen, UK;

1087. Biomedical Engineering, University of Bridgeport, CT, USA;

1088. Pharmaceutical Sciences, Texas Tech University Health Sciences Center, Lubbock, TX, USA;

1089. Ecosystem and Conservation Sciences, University of Montana, Missoula, MT, USA;

1090. Department of Systems Neuroscience, University of Goettingen, Germany;

1091. School of Public Affairs, Penn State Harrisburg, PA, USA;

1092. Bristol Medical School, University of Bristol, UK;

1093. Water Resources and Environmental Modeling, Czech University of Life Sciences, Prague, Czech Republic;

1094. Neurology, Gardner Family Center for Parkinson’s Disease and Movement Disorders, University of Cincinnati, OH, USA;

1095. Melbourne School of Population and Global Health, University of Melbourne, Parkville, VIC, Australia;

1096. Christie Patient Centred Research, The Christie NHS Foundation Trust, Manchester, UK;

1097. Environmental Health Science and Engineering, Jimma University;

1098. Laboratory of Systems Genetics, National Heart, Lung, and Blood Institute, Bethesda, MD, USA;

1099. Department of Medicine, University of Oslo, Norway;

1100. Medicine, University of Sydney, NSW, Australia;

1101. Clarendon Laboratory, Department of Physics, University of Oxford, UK;

1102. Biochemistry, University of Cambridge, UK;

1103. Biochemical Plant Physiology, Heinrich Heine University, Duesseldorf, Germany;

1104. Department of Respiratory Medicine, Norfolk and Norwich University Hospital NHS Foundation Trust, UK;

1105. Imaging, Cleveland Clinic Lou Ruvo Center for Brain Health, Las Vegas, NV, USA;

1106. Nutrition and Dietetics, College of Nursing and Health Sciences, Flinders University, Adelaide, SA, Australia;

1107. School of Medicine, University of Dundee, UK;

1108. Laboratory of Optics and Dynamics of Biological Systems, Novosibirsk State University, Russia;

1109. Department of Mathematics, University of Padova, Italy;

1110. Clinical Research, London School of Tropical Medicine, UK;

1111. Sciences naturelles, UQO, Ripon, QC, Canada;

1112. Faculty of Law, Lund University, Lund, Sweden;

1113. Department of Microbiology and Immunology, University of Gothenburg, Sweden;

1114. Biology Department, Stanford University, Palo Alto, CA, USA;

1115. Department of Genetics and Biosystematics, University of Gdansk, Poland;

1116. Kachwekano Zardi, National Agricultural Research Organization (NARO), Entebbe, Uganda;

1117. Bachelor Program in Interdisciplinary Studies, National Yunlin University of Science &

Technology, Yunlin County, Taiwan;

1118. Clinical Genome Analysis Branch, National Cancer Center, Goyang, Korea;

1119. Danish Research Institute of Translational Neuroscience (DANDRITE), Department of Biomedicine, Aarhus University, Aarhus, Denmark;

1120. Biostatistics, Yale University, New Haven, CT, USA;

1121. Centre de Recherches Insulaires et Observatoire de l’Environnement (CRIOBE), PSL University Paris: EPHE-UPVD-CNRS USR 3278 CRIOBE, University of Perpignan via Domitia, France;

1122. Mathematics and Computer Sciences, Eduardo Mondlane University, Maputo, Mozambique;

1123. Department of Old Age Psychiatry and Psychotherapy, University of Bern, Switzerland;

1124. Kinesiology and Health Science, Biola University, La Mirada, CA, USA;

1126. Laboratory of Cytology of Unicellular Organisms, Institute of Cytology RAS, Saint Petersburg, Russia;

1127. Faculty of Engineering and Information Technology, University of Wollongong, Wollongong, NSW, Australia;

1128. School of Chemistry and Chemical Engineering, Beijing Institute of Technology, Beijing, China;

1129. Food Processing, Research Institute of Food Science and Technology (RIFST), Mashhad, Iran;

1130. Cell and Developmental Biology, Centre for Genomic Regulation, Barcelona, Spain;

1131. Queensland Alliance for Agriculture and Food Innovation, The University of Queensland, Brisbane, QLD, Australia;

1132. Cell Biology, Rostock University Medical Center, Germany;

1133. Genetics and Genomics, The Roslin Institute, University of Edinburgh, UK;

1134. Biochemistry, College of Medicine, Mohammed Bin Rashid University of Medicine and Health Sciences, Dubai, UAE;

1135. Genomics Centre & Centre for Integrative Ecology, Deakin University, Geelong, VIC, Australia;

1136. Division of Rehabilitation and Agein, University of Nottingham, UK;

1137. Engineering, University of Nottingham, UK;

1138. Immunology, Garvan Institute, Sydney, NSW, Australia;

1139. Auckland Bioengineering Institute, University of Auckland, New Zealand;

1140. Department of Medical Parasitology and Infection Biology, Swiss Tropical and Public Health Institute, University of Basel, Switzerland;

1141. Inhalation Toxicology, Fraunhofer Institute for Toxicology and Experimental Medicine (ITEM), Hannover, Germany;

1142. Department of Rheumatology and Internal Medicine, Wroclaw Medical University, Poland;

1143. BCRT, Charité Medical University of Berlin, Germany;

1144. Public Health, The University of Hong Kong, Hong Kong, China;

1145. Experimental Psychology, University of Oxford, UK;

1146. School of Life Sciences, Hubei Key Laboratory of Genetic Regulation and Integrative Biology, Central China Normal University, Hubei, China;

1147. Department for Internal Medicine II, University Clinic Krems, Karl Landsteiner University for Health Sciences;

1148. Anesthesia and Pain Medicine, University Health Network and University of Toronto, ON, Canada;

1149. Institute of Earth and Environmental Science, University of Potsdam, Germany;

1150. Department for Health, University of Bath, UK;

1151. Institute of Organic Chemistry, Justus-Liegbig University, Giessen, Germany;

1152. Computational and RNA Biology, University of Copenhagen, Denmark;

1153. Bedford Institute of Oceanography, Fisheries and Oceans Canada, Dartmouth, NS, Canada;

1154. Integrative Medicine & Clinical Naturopathy, Institute for Social Medicine, Epidemiology and Health Economics, Charité Medical University of Berlin, Germany;

1155. Physical Biology, BMLS, CEF-MC, Goethe Universität, Frankfurt am Main, Germany;

1156. Viral Diseases, Sciensano, Ixelles, Belgium;

1157. Department of Psychiatry, Faculty of Medicine, Public Health and Nursing, Gadjah Mada University, Yogyakarta, Indonesia;

1158. Anatomy and Structural Biology, Albert Einstein College of Medicine, New York City, NY, USA;

1159. Nephrology and Hypertension, St. Marianna University, Kanagawa, Japan;

1160. Office of the Queensland Chief Scientist, Department of Environment and Science, Queensland Government, Brisbane, QLD, Australia;

1161. Department of Surgical Sciences, Section of Vascular Surgery, Uppsala University, Uppsala, Sweden;

1162. Animal Science, University of Connecticut, Storrs, CT, USA;

1163. Research, Children’s Cancer Hospital, Cairo, Egypt;

1164. Department of Earth, Ocean, and Atmospheric Science, Florida State University, Tallahasse, FL, USA;

1165. Unité Biologie Fonctionnelle et Adaptative (BFA) CNRS UMR8251, Université Paris Diderot, Paris, France;

1166. School of Biochemistry, University of Bristol, UK;

1167. Department of Anatomy, Faculty of Medicine, Trakia University, Stara Zagora, Bulgaria;

1168. Bio Pilot Plant, Leibniz Institute for Natural Product Research and Infection Biology, Jena, Germany;

1169. Institute of Research and Development, Duy Tan University, Da Nang, Vietnam;

1170. Helsinki Institute of Life Science (HiLIFE), University of Helsinki, Finland;

1171. Department of Animal Biology, Federal University of Technology Minna, Nigeria;

1172. Institute of Chinese Medical Sciences, University of Macau, Guangdong, China;

1173. Australian Institute for Bioengineering and Nanotechnology, The University of Queensland, Brisbane, QLD, Australia;

1174. Research Institute of Petroleum Industry (RIPI), NIOC, Tehran, Iran;

1175. Bioanalysis, Ghent University, Belgium;

1176. Department of Dermatology, The First Affiliated Hospital, Sun Yat-sen University, Guangzhou, Guangdong, China;

1177. School of Business, Dalian University of Technology, Liaoning, China;

1178. Faculty of Kinesiology, University of Calgary, AB, Canada;

1179. Department of Psychological Medicine, Weston Education Centre, King’s College London, UK;

1180. Chief Scientist, The George Institute for Global Health, Sydney, NSW, Australia;

1181. Radiology and Chemistry, UCSF, San Francisco, CA, USA;

1182. Griffith Institute for Drug Discovery, Griffith University, Brisbane, QLD, Australia;

1183. Centre of Biology and Health Sciences, Mackenzie Presbyterian University, São Paulo, Brazil;

1184. Neuropsychiatry, University of Pennsylvania, Philadelphia, PA, USA;

1185. School of Environmental and Municipal Engineering, Tianjin Chengjian University, Tianjin, China;

1186. Shandong Provincial Key Laboratory of Biophysics, College of Physics and Electronic Information, Dezhou University, Dezhou, China;

1187. Int Med-Hematology/Oncology, University of Michigan, MI, USA;

1188. College of Life Sciences, Henan Agricultural University, Zhengzhou, China;

1189. Department of Transufusion Medicine, National Institutes of Health, Bethesda, MD, USA;

1190. Beijing Children’s Hospital, Capital Medical University, Beijing, China;

1191. Public health, The University of Tokyo, Japan;

1192. Biomedical Genomics and Oncogenetics Research Laboratory, Faculty of Sciences and Techniques of Tangier, Abdelmalek Essaadi University, Morocco;

1193. The First Affiliated Hospital, Sun Yat-sen University, Guangzhou, Guangdong, China;

1194. Department of Medicine, University of Illinois at Chicago, IL, USA;

1195. Department of Occupational Health, School of Public Health, Shaanxi University of Chinese Medcine, Shaanxi, China;

1196. Department of Critical Care Medicine, Affiliated Hospital of Zunyi Medical University, Guizhou, China;

1197. State Key Laboratory of Pathogen and Biosecurity, Beijing Institute of Microbiology and Epidemiology, Beijing, China;

1198. Key Laboratory of Neonatal Disease, Ministry of Health, Shanghai, China;

1199. Institute of Biophysics, Dezhou University, Shandong, China;

1200. Pathology, Fudan University Shanghai Cancer Center, Shanghai, China;

1201. Department of Physics, Covenant University, Ota, Nigeria;

1202. Institute of Evolutionary Biology, University of Edinburgh, UK;

1203. Department of Physical Therapy and Health Rehabilitation, Prince Sattam Bin Abdulaziz University, Al Kharj, Saudi Arabia;

1204. Chemical Engineering, Loughborough University, UK;

1205. Physiotherapy, Umm Al-Qura University, Mecca, Saudi Arabia;

1206. Department of Computer Science, University of Liverpool, UK;

1207. Cognitive Science, Lund University, Malmö, Sweden;

1208. Department of Public and Environmental Health, National Open University of Nigeria, Abuja, Nigeria;

1209. Department of Social Policy and Intervention, University of Oxford, UK;

1210. Physics of Complex Systems, Weizmann Institute of Science, Rehovot, Israel;

1211. Department of Biology and Ecology, Faculty of Science, University of Ostrava, Czech Republic;

1212. Agricultural Sciences, University of Helsinki, Finland;

1213. Bioinformatics Unit, Max Planck Institute of Immunobiology and Epigenetics, Freiburg, Germany;

1214. Mathematics Education, University of Regensburg, Germany;

1215. Public Health, Ghana Health Services;

1216. The Wales Centre for Podiatric Studies, Cardiff Metropolitan University, Cardiff, UK;

1217. Institute of Public Health - Section of Hygiene, Catholic University of the Sacred Heart, Rome, Italy;

1218. Computational Bioscience Program, University of Colorado Anschutz Medical Campus, CO, USA;

1219. Department of Laboratorial Science and Technology, School of Public Health, Peking University, Beijing, China;

1220. Menzies Centre for Health Policy, School of Public Health, University of Sydney, NSW, Australia;

1221. College of Life Sciences, Heilongjiang University, Harbin, China;

1222. Department of Computer Science, University of Southern California, Los Angeles, CA, USA;

1223. Department of Electrical and Computer Engineering, University of Auckland, New Zealand;

1224. College of Life Sciences, Zhejiang Sci-Tech University, Zhejiang, China;

1225. College of Chemistry and Chemical Engineering, Xianyang Normal College, Shannxi, China;

1226. Research Center of TCM Information Engineering, Beijing University of Chinese Medicine, Beijing, China;

1227. Department of Surgery, Seoul National University Bundang Hospital, Seoul National University College of Medicine, Seongnam, Korea;

1228. Maternal and Child Health Division, International Centre for Diarrhoeal Diseases Research, Bangladesh;

1229. Department of Forensic Medicine, University of Copenhagen, Denmark;

1230. Biological Sciences, University of Southampton, UK;

1231. Cardiac surgery, Niguarda Hospital, Milan, Italy;

1232. Hearing and Speech Sciences, Vanderbilt University, Nashville, TN, USA;

1233. Faculty of Biology, Medicine and Health, University of Manchester, UK;

1234. Clinic for Horses, University of Veterinary Medicine Hannover, Germany;

1235. NeuroPSI - UMR9197, CNRS, France;

1236. Agricultural Economics, Bangladesh Agricultural University;

1237. Department of Surgical Oncology, The Second Affiliated Hospital of Zhejiang University, Zhejiang University, Hangzhou, Zhejiang, China;

1238. Laboratory of Medical Genetics, Harbin Medical University, Heilongjiang, China;

1239. State Key Laboratory of Genetic Engineering, Collaborative Innovation Center for Genetics and Development, School of Life Sciences, Fudan University, Shanghai, China;

1240. Biology, Natural History Museum of Denmark;

1241. Department of Psychology, University of Saskatchewan, Saskatoon, SK, Canada;

1242. Laboratoire Matière et Systèmes Complexes, Université Paris Diderot, France;

1243. Epidemiology and Biostatistics, Bahir Dar University, Ethiopia;

1244. Centre of Studies in Geography and Spatial Planning (CEGOT), University of Coimbra, Portugal;

1245. Computer Science and Engineering, University of Nebraska-Lincoln, NE, USA;

1246. Epidemiology, University Medical Center Groningen, University of Groningen, Netherlands;

1247. Department of Radiology, University College London Hospital NHS Foundation Trust, and Division of Surgery & Interventional Science, University College London, London, UK;

1248. Department of Neurology and Neurosurgery, South Central High Specialty Hospital, Pemex, Mexico;

1249. College of Information Science and Engineering, Shandong Agricultural University, Taian, Shandong, China;

1250. College of Life Sciences, Sichuan University, Sichuan, China;

1251. National drug Research Institute, Curtin University, Perth, WA, Australia;

1252. Gibson Lab, Stowers Institute for Medical Research, Kansas City, MO, USA;

1253. Bacteriology and Epidemiology, Wageningen Bioveterinary Research, Lelystad, Netherlands;

1254. General Practice, University Medical Center Göttingen, Germany;

1255. Novo Nordisk Foundation Center for Protein Research, Proteomics Program, Faculty of Health and Medical Sciences, University of Copenhagen, Denmark;

1256. Department of Rheumatology and Immunology, Guangdong Second Provincial General Hospital, Guangzhou, China;

1257. Biomedical Rngineering, Erasmus University Medical Center, Rotterdam, Netherlands;

1258. Pharmacology and Toxicology, University Medical Center Goettingen, Goettingen, Germany;

1259. Graduate School of Biomedical & Health Sciences, Hiroshima University, Japan;

1260. Science and Engineering, University of the Sunshine Coast, QLD, Australia;

1261. Center for Advancing Electronics Dresden, Technische Universitat Dresden, Germany;

1262. School of Health Sciences, University of Iceland, Reykjavík, Iceland;

1263. Health Promotion, Ministry of Health and Child Care, Zimbabwe;

1264. Material Science and Engineering, The Ohio State University, Columbus, OH, USA;

1265. Computational and Systems Biology, University of Pittsburgh, PA, USA;

1266. School of Chemical Biology & Biotechnology, Peking University Shenzhen Graduate School, Shenzhen, China;

1267. School of Materials, University of Manchester, UK;

1268. DPECS, Erasmus University Rotterdam, Netherlands;

1269. Molecular Biology, Centro de Biologia Molecular Severo Ochoa, Madrid, Spain;

1270. Radiotherapy and Imaging, The Institute of Cancer Research, London, UK;

1271. Neurology, Ewha Womans University School of Medicine and Ewha Medical Research Institute, Seoul, Korea;

1272. Plastic and Reconstructive Surgery, Matsunami General Hospital, Kasamatsu, Japan;

1273. Department of Dermatology, University Medical Center Groningen, University of Groningen, Netherlands;

1274. Centre for Global Health Research, University of Edinburgh, UK;

1275. Department of Construction Management, Harbin Institute of Technology, Heilongjiang, China;

1276. Institute of Zoology, University of Veterinary Medicine Hannover, Germany;

1277. National Laboratory of Solid State Microstructures, Department of Physics, and Collaborative Innovation Center of Advanced Microstructures, Nanjing University, Nanjing, China;

1278. Proteomics, Biomedical Research Foundation, Academy of Athens, Greece;

1279. Genomics, ICAR-National Research Centre on Plant Biotechnology, New Delhi, India;

1280. School of Public Health, The University of Hong Kong, Hong Kong, China;

1281. Health Sciences, George Davies Centre for Medicine, University of Leicester, UK;

1282. Bioengineering, University of Illinois at Chicago, IL, USA;

1283. Hellenic Cord Blood Bank, Biomedical Research Foundation Academy of Athens, Greece;

1284. Beijing Advanced Innovation Center for Structural Biology, Tsinghua University, Beijing, China;

1285. Faculty of Health and Wellbeing, Sheffield Hallam University, Sheffield, UK;

1286. Bioinformatics Institute, University of Missouri, Columbia, MO, USA;

1287. Quality Functional Unit, Hospital Doctor Moliner, Valencia, Spain;

1288. Plants and Crops, Ghent University, Belgium;

1289. Department of Biomedical and Neuromotor Sciences, University of Bologna, Italy;

1290. Center for Micro-Biorobotics, Istituto Italiano di Tecnologia, Genoa, Italy;

1291. Department of Bioinformtaics and Biostatistics, Shanghai Jiao Tong University, Shanghai, China;

1292. Health System Strengthening, Baylor College of Medicine Children’s Foundation - Uganda;

1293. Medical Cell BioPhysics, University of Twente, Enschede, Netherlands;

1294. Department of Pharmaceutical Sciences, University of Kashmir;

1295. Biologic and Materials Sciences, University of Michigan, MI, USA;

1296. Faculty of Health, Queensland University of Technology, Brisbane, QLD, Australia;

1297. School of Medicine, Shanghai Jiao Tong University, Shanghai, China;

1298. Surgery, Aga Khan University, Karachi, Pakistan;

1299. School of Veterinary Science, Massey University, Auckland, New Zealand;

1300. Department of Clinical Psychology and Psychotherapy, Institute of Psychology, University of Freiburg, Germany;

1301. Centre for Integrative Ecology, Deakin University, Melbourne, VIC, Australia;

1302. State Key Laboratory Of Molecular Reaction Dynamics, Dalian Institute of Chemical Physics, Chinese Academy of Sciences, Liaoning, China;

1303. Social Psychology, Tilburg University, Netherlands;

1304. Emergency Medicine, University of Wisconsin-Madison, WI, USA;

1305. Learning Informatics Management and Ethics (LIME), Karolinska Institute, Solna, Sweden;

1306. Graduate Program in Quantitative and Computational Biosciences, Baylor College of Medicine, Houston, TX, USA;

1307. Department of Psychiatry, University Hospital of Reims, France;

1308. Department of Inorganic and Analytical Chemistry, Rzeszow University of Technology, Poland;

1309. Centre for Biotechnology, Siksha O Anusandhan Universityr, Odisha, India;

1310. School of Medicine and Public Health, The University of Newcastle, Callaghan, NSW, Australia;

1311. Institute of Health Promotion and Clinical Movement Science, German Sport University Cologne, Germany;

1312. Department for Gynecology and Obstetrics, University Hospital Essen, Germany;

1313. Médecine Sociale et Préventive, Universite de Montreal, Montreal, QC, Canada;

1314. Intitute for Separation and Process Technology, University of Technology Clausthal, Germany;

1315. Edmond & Lily Safra Center for Brain Sciences, Hebrew University of Jerusalem, Israel;

1316. Department of Pathology, University of California San Diego, CA, USA;

1317. Department of Physiotherapy, Kathmandu University School of Medical Sciences, Dhulikhel, Nepal;

1318. Accounting and Finance, University of Limerick, Ireland;

1319. Division of Nursing, Midwifery and Social Work, School of Health Sciences, Faculty of Biology, Medicine and Health, University of Manchester, UK;

1320. School of Biomedical Sciences, The University of Queensland, Brisbane, QLD, Australia;

1321. Division of Psychiatry, University College London, UK;

1322. Image Guided Therapies, Fraunhofer MEVIS, Bremen, Germany;

1323. Polymer Science and Engineering, University of Science and Technology of China, Anhui, China;

1324. Haematology-Oncology, National University Health System, Singapore;

1325. Laboratory of Marine Biotechnology, Institute of Bioscience, Universiti Putra Malaysia, Selangor, Malaysia;

1326. Breast Oncology, Sun Yat-sen University Cancer Center, Guangzhou, China;

1327. Unit of Bacteriology-Virology, Centre de Recherche Medicale et Sanitaire, Niamey, Niger;

1328. Laboratory Animal Centre, Bengbu Medical College, Anhui, China;

1329. Pharmacology and Toxicology, Medical College of Wisconsin, Wauwatosa, WI, USA;

1330. School of Chemistry, Physics and Mechanical Engineering, Science and Engineering Faculty, Queensland University of Technology, Brisbane, QLD, Australia;

1331. Institution for Social Medicine and Family Medicine, School of Public Health, Zhejiang University, Hangzhou, Zhejiang, China;

1332. School of Computer Science and Technology, Harbin Institute of Technology, Heilongjiang, China;

1333. Electrical and Computer Engineering, Mississippi State University, Mississippi State, MS, USA;

1334. Department of Molecular Biotechnology and Health Sciences, University of Turin, Italy;

1335. Electrical and Computer Engineering, University of Toronto, ON, Canada;

1336. Physiotherapy Department, School of Rehabilitation, Tehran University of Medical Sciences, Iran;

1337. Department of Occupational Health, School of Health, Shiraz University of Medical Sciences, Iran;

1338. Department of Forest Sciences, University of Helsinki, Finland;

1339. Department of Surgery, Weifang Medical University, Weifang, China;

1340. School of Computational Science and Engineering, Georgia Institute of Technology, Atlanta, GA, USA;

1341. Key Laboratory of Chemical Genomics, Peking University Shenzhen Graduate School, Shenzhen, China;

1342. Computer Science and Technology, Tsinghua University, Beijing, China;

1343. Center for Excellence in Biomacromolecules, Institute of Biophysics, Chinese Academy of Sciences, Beijing, China;

1344. Human Pathology, University of Messina &

AO Papardo Hospital Messina, Italy;

1345. Institute of Child Health, College of Medicine, University of Ibadan, Nigeria;

1346. Paediatric Surgery, University Hospital of Alexandroupolis, Dragana, Greece;

1347. Faculty of Ophthalmology, College of Medicine, King Faisal University, Al-Hasa, Saudi Arabia;

1348. Department of Hematology. Sultan Qaboos University Hospital, Al Khod, Oman;

1349. Gulfstream Genomics, Gulfstream Diagnostics, Dallas, TX, USA;

1350. Institute of Molecular Medicine, University of Southern Denmark, Denmark;

1351. Institute for Social Marketing, University of Stirling, UK;

1352. Laboratory Sciences & Services Division, International Centre for Diarrhoeal Diseases Research, Bangladesh;

1353. Social Determinants of Health Research Center, Saveh University of Medical Sciences, Saveh, Iran;

1354. Gakujutsu Shien Co. Ltd., Tokyo, Japan;

1355. Institute of Geochemistry, Chinese Academy of Sciences, Guiyang, China;

1356. Medical School, University of Plymouth, UK;

1357. CDER/OPQ/OBP/DBRRII, U.S. Food and Drug Administration, Silver Spring, MD, USA;

1358. Department of Endocrinology, Affiliated Hospital of Jining Medical University, Shandong, China;

1359. Pediatric Hematology/Oncology, Children’s Hospital & Medical Center, Omaha, NE, USA;

1360. PWMHU, SJOG Burwood Hospital and UNSW Sydney, NSW, Australia;

1361. immunology, CHU de Toulouse, France;

1362. Medicine, Harvard Medical School, Boston, MA, USA;

1363. Cardiology, Barts Health NHS Trust, London, UK;

1364. Centre for Ecology, Evolution and Environmental Changes, Azorean Biodiversity Group and Universidade dos Açores, Azores, Portugal;

1365. Social Psychology, University of Valencia, Spain;

1366. Department of Advanced Biomedical Sciences, University of Naples ”Federico II”, Italy;

1367. Orthopaedic Surgery, Rochester General Hospital, Pittsford, NY, USA;

1368. RIKEN Center for Integrative Medical Sciences, Yokohama, Japan;

1369. Medicine, Columbia University Medical Center, New York, NY, USA;

1370. Holistic Education Center, Mackay Medical College, New Taipei City, Taiwan;

1371. Physiology, Southeast University, Jiangsu, China;

1372. Division of Epidemiology, Indian Council of Medical Research, National Institute of Cholera and Enteric Diseases, India;

1373. Department of Global Health, School of Public Health, Peking University, Beijing, China;

1375. Department of Neurology, Chang Gung Memorial Hospital Linkou Medical Center and College of Medicine, Chang-Gung University, Taoyuan, Taiwan;

1376. Cardiology, Saint Luke’s Mid America Heart Institute, Kansas City, MO, USA;

1377. Biomedical Sciences and Pharmacy Departments, College of Medicine, University of Malawi, Blantyre, Malawi;

1378. Genomics Research Center, Academia Sinica, Taipei, Taiwan;

1379. Instituto de Ciencias Biologicas, Universidade Federal de Goias, Goiânia, Brazil;

1380. Retina department, Clínica Vista, Lima, Perú

1381. Department of Immunology and Oncology, National Center for Biotechnology (CNB-CSIC), Madrid, Spain;

1382. Pathology Department, CHU Bicêtre, France;

1383. Biocuration, Bioself Communication, Marseille, France;

1384. School of Biomedical Engineering, School of Ophthalmology & Optometry and Eye Hospital, Wenzhou Medical University, Zhejiang, China;

1385. Pediatrics I, Renmin Hospital, Hubei University of Medicine, Hubei, China;

1386. Anesthesiology, Perioperative Care and Pain Medicine, New York University School of Medicine, New York, NY, USA;

1387. Histology, Zagazig University, Egypt;

1388. Department of Neurology, Peking University Third Hospital, Beijing, China;

1389. Department of Pathology, College of Health Sciences, Faculty of Basic Clinical Sciences, Ahmadu Bello University, Zaria, Nigeria;

1390. School of Computer and Communication Engineering, University of Science and Technology, Beijing, China;

1391. Caribbean Institute for Health Research, The University of the West Indies, Jamaica;

1392. Human Pathology of the Adult and Evolutive Age ”Gaetano Barresi”, Section of General Surgery, University of Messina, Italy;

1393. Ageing Clinical and Experimental Research (ACER) Team, University of Aberdeen, UK;

1394. Ophthalmology, Massachusetts Eye Research and Surgery Institution, Waltham, MA, USA;

1395. Child & Adolescent Psychiatry, Child Study Center at Hassenfeld Children’s Hospital of New York at NYU Langone, NY, USA;

1396. Department of Thoracic Surgery, National Cancer Center / Cancer Hospital, Chinese Academy of Medical Sciences, Beijing, China;

1397. Endowed Health Services Research, School of Medicine, University of Puerto Rico, Mayagüez, PR, USA;

1398. Pathology and Hamon Center for Therapeutic Oncology Research, UT Southwestern Medical Center, Dallas, TX, USA;

1399. Department of Anesthesia, Pain Management, and Perioperative Medicine, Dalhousie University, Halifax, NS, Canada;

1400. Faculty of Mathematics & Technology, Koblenz University of Applied Sciences, Germany;

1401. VisMederi srl, Siena, Italy;

1402. Medicine, Ross University School of Medicine, Miramar, FL, USA;

1403. Cancer Research UK and UCL Cancer Trials Centre, University College London, UK;

1404. Department of Critical Care, University of Alberta, Edmonton, AB, Canada;

1405. Critical Care Medicine, Dalhousie University, Halifax, NS, Canada;

1406. Clinical Haematology, Palmerston North Hospital, New Zealand;

1407. Family Medicine, University of Ottawa, ON, Canada;

1408. College of Engineering, China Agricultural University, Beijing, China;

1409. NHMRC Clinical Trials Centre, University of Sydney, NSW, Australia;

1410. Division of Drug Discovery and Safety, Leiden Academic Centre for Drug Research, Leiden University, Netherlands;

1411. Biomedical Sciences Department of ESTESC - Coimbra Health School, Polytechnic Institute of Coimbra, Portugal;

1412. Department of Interventional Radiology, The First Affiliated Hospital, Sun Yat-sen University, Guangzhou, Guangdong, China;

1413. Pathology, Microbiology, and Immunology, Vanderbilt University Medical Center, TN, USA;

1414. Infectious Diseases, American University of Beirut Medical Center, Beirut, Lebanon;

1415. Pediatrics, University of New Mexico, Albuquerque, NM, USA;

1416. Department of Social Work, Social Care and Community Studies, Sheffield Hallam University, Sheffield, UK;

1417. Pulmonary Diseases, Critical Care & Environmental Medicine, Tulane University School of Medicine, New Orleans, LA, USA;

1418. Viral Hepatitis, Mechnikov Research Institute for Vaccines and Sera, Moscow, Russia;

1419. Neurosurgery, Duke University Medical Center, Durham, NC, USA;

1420. Internal Medicine, University Tübingen, Germany;

1421. Pediatrics, University of Ottawa, ON, Canada;

1422. Medicine, Penn State College of Medicine, Hershey, PA, USA;

1423. Urology, University of Texas Health Science Center at San Antonio, TX, USA;

1424. Department for Wound Infection Treatment and Prevention, Vreden Russian Research Institute of Traumatology and Orthopaedics, St. Petersburg, Russia;

1425. Medicine, Western University, London, ON, Canada;

1426. Laboratoire Cancérologie Mammaire / Institut J. Bordet, Université Libre de Bruxelles (ULB), Brussels, Belgium;

1427. College of Life Science and Technology, Huazhong University of Science and Technology, Hubei, China;

1428. Department for TB Control and Prevention, Hangzhou Center for Disease Control and Prevention, Zhejiang, China;

1429. Institute of Aging Research, School of Medicine, Hangzhou Normal University, Zhejiang, China;

1430. Department of Rheumatology and Clinical Immunology, Key Laboratory of Rheumatology and Clinical Immunology, Ministry of Education, Peking Union Medical College Hospital, Chinese Academy of Medical Sciences and Peking Union Medical College, Beijing, China;

1431. Department of Biotechnology, Kaohsiung Medical University, Taiwan;

1432. Oncology, West China Hospital, Sichuan, China;

1433. School of Medicine, University of California Irvine, CA, USA;

1434. Diagnostics, Zoetis, Kalamazoo, MI, USA;

1435. Clinical Pathology Laboratory, Vito Fazzi General Hospital, Lecce, Italy;

1436. Head and Neck Oncology Research, Aintree University Hospital NHS Trust, Liverpool, UK;

1437. Department of Otolaryngology, Head Neck Surgery, Beijing Friendship Hospital, Capital Medical University, Beijing, China;

1438. Trauma & Orthopaedics, Wrightington Hospital, Wigan, Greater Manchester, UK;

1439. Physical and Environmental Sciences, University of Toronto, Scarborough, ON, Canada;

1440. Microbiology and Immunology, University of Rochester, NY, USA;

1441. Department of Psychiatry, Loyola University Medical Center, Maywood, IL, USA;

1442. Microbiology, Atkins Veterinary Services, Calgary, AB, Canada;

1443. Microbiology, Golestan University of Medical Sciences, Iran;

1444. CIAFEL, FADEUP, University of Porto, Portugal;

1445. Hematology-Oncology, Mashhad University of Medical Science, Iran;

1446. Global Health, Stellenbosch University, South Africa;

1447. Anaesthesiology, Kovai Medical Center and Hospital, Tamilnadu, India;

1448. Saw Swee Hock School of Public Health, National University of Singapore;

1449. Pediatric cardiology, Queen Alia Heart Institute, Royal Medical Services, Amman;

1450. Department of Food Science and Biotechnology, College of Biotechnology and Bioscience, Kangwon National University, Chuncheon, Korea;

1451. Internal Medicine, Kakatiya Medical College, Telangana, India;

1452. Otolaryngology, St. Paul’s Hospital, Vancouver, Canada;

1453. Neurology, Kakatiya Medical College and Mahatma Gandhi Memorial Hospital, Warangal, India;

1454. CIBAV Research Group, Veterinary Medicine School, University of Antioquia, Medellin, Colombia;

1455. Paediatrics, King’s College Hospital NHS Trust, London, UK;

1456. Neurosurgery, Carlo Besta Neurological Institute Milan, Italy;

1458. Department of Clinical Microbiology and Microbial Pathogenesis, School of Medicine, University of Crete, Greece;

1459. Soft and Active Matter Group, Department of Physics, Indian Institute of Science Education and Research (IISER) Tirupati, Andhra Pradesh 517507, India;

1460. Center of Studies in Physical Activity Measurements, School of Medicine and Health Sciences, Universidad del Rosario, Bogota D.C, Colombia;

1461. Medicine, Rheumatology, St. Clares Mercy Hospital, St. Johns, NL, Canada;

1462. Department of Community Dentistry and Population Health, University of Colorado Anschutz Medical Campus, CO, USA;

1463. Cardiology and Vascular Medicine, University Hospital Essen, Germany;

1464. Endocrinology, University Hospital Basel, Switzerland;

1465. Institute of Medical Genetics and Applied Genomics, University Tübingen, Germany;

1466. Department of Medicine D, Division of General Internal Medicine, Nephrology, and Rheumatolog, University Hospital Münster, Germany;

1467. Nutrition, Harvard TH Chan School of Public Health, Boston, MA, USA;

1468. Department of Nephrology, University of Kentucky, Lexington, KY, USA;

1469. Emergency Medicine, University of Missouri, Columbia, MO, USA;

1470. Department of Anaesthesiology and Critical Care, Medical Centre, University of Freiburg, Germany;

1471. Quality and Safety, Children’s Hospital of The King’s Daughters, Norfolk, VA, USA;

1472. Research, IIHMR University, Rajasthan, India;

1473. Department of Internal Medicine, University of Arkansas for Medical Sciences, Little Rock, AR, USA;

1474. Infectious Diseases, Cincinnati Children’s Hospital Medical Center, OH, USA;

1475. Mood Disorders Psychopharmacology Unit, University Health Network, Toronto, ON, Canada;

1476. Department of Medicine, University of California Irvine, Orange, CA, USA;

1477. Center for Reliability Science and Technology, Chang Gung University, Taoyuan, Taiwan;

1478. Gastroenterology, Profensa, La Pampa, Argentina;

1479. Centre for Global Development and Institute of Applied Health Sciences, University of Aberdeen, UK;

1480. Ophthalmology, Harvard Medical School, Boston, MA, USA;

1481. Department of Urology, University Hospitals Leuven, Belgium;

1482. Medical School, University of Talca, Chile;

1483. Institute of Biotechnology and Department of Life Science, National Tsing Hua University, Hsinchu, Taiwan;

1484. College of Life Sciences, Chinese Academy of Sciences, Beijing, China;

1485. Department of Obstetrics and Gynaecology, Medical and Health Sciences, University of Auckland, New Zealand;

1486. School of Clinical Medicine, University of Cambridge, UK;

1487. Orthopaedics & Rehabilitation, University of Florida, Gainesville, FL, USA;

1488. Otolaryngology, Massachusetts Eye and Ear Infirmary, Boston, MA, USA;

1489. Chongqing Key Laboratory of Molecular Oncology and Epigenetics, The First Affiliated Hospital of Chongqing Medical University, Chongqing, China;

1490. Department of Pulmonary &

Critical Care Medicine, Chinese PLA General Hospital, Beijing, China;

1491. Department of Chemical Engineering, Tsinghua University, Beijing, China;

1492. Institute of Intelligent Machines, Hefei Institutes of Physical Science, Chinese Academy of Sciences, Anhui, China;

1493. Department of Instrumental and Electrical Engineering, Xiamen University, Fujian, China;

1494. Department of Pharmacology, Yonsei University College of Medicine, Seoul, Korea;

1495. Nephrology, Walter Reed National Military Medical Center, Bethesda, MD, USA;

1496. Department of Pediatrics, Eastern Virginia Medical School, Children’s Hospital of The King’s Daughters, Norfolk, VA, USA;

1497. Department of Mathematics, College of Science, China Three Gorges University, Yichang, China;

1498. College of Information Engineering, Xiangtan University, Hunan, China;

1499. Mood Disorders and Psychopharmacology, University Health Network, Toronto, ON, Canada
