# Erratum to: Large expert-curated database for benchmarking document similarity detection in biomedical literature search

**DOI:** 10.1093/database/baz138

**Published:** 2020-07-18

**Authors:** Peter Brown, RELISH Consortium, Yaoqi Zhou

**Affiliations:** 1 School of Information and Communication Technology, Griffith University, Gold Coast, QLD, Australia; 2 Institute for Glycomics, Griffith University, Gold Coast, QLD, Australia; 3 See collaborators section

This manuscript has been updated in order to amended incorrect affiliation indices for several authors. The publisher apologies for any confusion caused.

